# Template-directed synthesis of linear porphyrin oligomers: classical, Vernier and mutual Vernier†
†Electronic supplementary information (ESI) available: Synthesis and characterization of new compounds, ladder complexes, UV-vis-NIR titrations and binding data for reference compounds and for the formation of linear oligomer complexes, calculation of effective molarities, analytical GPC calibration and molar absorption coefficients. See DOI: 10.1039/c6sc05355f



**DOI:** 10.1039/c6sc05355f

**Published:** 2017-01-20

**Authors:** Nuntaporn Kamonsutthipaijit, Harry L. Anderson

**Affiliations:** a Department of Chemistry , University of Oxford , Chemistry Research Laboratory , Oxford OX1 3TA , UK . Email: harry.anderson@chem.ox.ac.uk

## Abstract

Three different types of template-directed syntheses of linear porphyrin oligomers are presented. In the classical approach the product has the same number of binding sites as the template, whereas in Vernier reactions the product has the lowest common multiple of the numbers of binding sites in the template and the building block. Mutual Vernier templating is like Vernier templating except that both strands of the Vernier complex undergo coupling simultaneously, so that it becomes impossible to say which is the ‘template’ and which is the ‘building block’. The template-directed synthesis of monodisperse linear oligomers is more difficult than that of cyclic oligomers, because the products of linear templating have reactive ends. All three types of templating are demonstrated here, and used to prepare a nickel(ii) porphyrin dodecamer with 4-pyridyl substituents on all twelve porphyrin units. The stabilities and cooperativities of the double-strand complexes involved in these reactions were investigated by UV-vis-NIR titration. The four-rung ladder duplex has a stability constant of about 2 × 10^18^ M^–1^ in dichloromethane at 298 K.

## Abstract 2

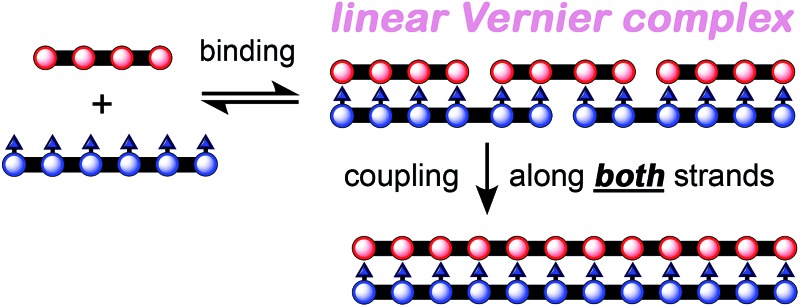
We demonstrate a variety of template-directed strategies for preparing linear monodisperse butadiyne-linked porphyrin oligomers by Glaser–Hay coupling, based on the coordination of pyridine-substituted nickel(ii) porphyrins to zinc(ii) porphyrins.

## Introduction

Linear π-conjugated porphyrin oligomers^1^ have been developed for a variety of applications in molecular electronics,^2–4^ photovoltaics^5^ and nonlinear optics,^6–8^ owing to their unusual electronic and optical properties. These molecular wires are excellent candidates for charge- and energy-transport over distances of 5–20 nm.^2–4,9^ However the synthesis of long monodisperse chains can be laborious. Here, we develop template-directed strategies for controlling the synthesis of linear porphyrin oligomers. One limitation of classical template-directed synthesis is the availability of the template, since it needs to be as long as the product. Here we show how the Vernier principle can solve this problem. The formation of supramolecular Vernier complexes was first suggested in a visionary review by Lindsey,^10^ by analogy with models for the self-assembly of collagen proteins.^11^ When there is a mismatch between the numbers of binding sites in two complementary molecules, they bind each other to form a Vernier complex in which the number of binding sites is the lowest common multiple of the numbers of sites in the two components. This principle has been applied to prepare double-strand^12^ and triple-strand^13^ non-covalent Vernier assemblies and dynamic covalent molecular ladders.^14^ Synthetic DNA nanostructures have also been created as Vernier complexes.^15,16^ Nevertheless, there have been no reports of subsequent ligation, to connect the linearly arranged components of a Vernier complex into a covalent strand. Previously, we demonstrated efficient cyclic Vernier templating, using the non-commensurate combination of a hexapyridyl template and a zinc porphyrin tetramer to synthesize a cyclic porphyrin dodecamer,^17,18^ and we extended this strategy to prepare rings of 10, 24, 30 and 40 porphyrin units.^19–21^

In template-directed macrocyclization reactions,^17–23^ a template which favors formation of a particular ring-size automatically prevents further coupling. However, in the template-directed synthesis of linear oligomers, the products typically have reactive termini, so that it becomes necessary to control the reaction time, or to add a capping reagent, to avoid uncontrolled polymerization. This lack of a “stop” signal makes the template-directed synthesis of linear oligomers more challenging than that of cyclic ones. On the other hand, linear systems present an interesting analogy to DNA replication, and they open up the exciting possibility of coupling along both strands of a Vernier complex, so that there is no longer a distinction between the ‘template’ and the ‘building block’. Here we test the scope of this strategy, which we call ‘mutual Vernier templating’, in the synthesis of linear porphyrin dodecamers.

## Approach

**Fig. 1:**
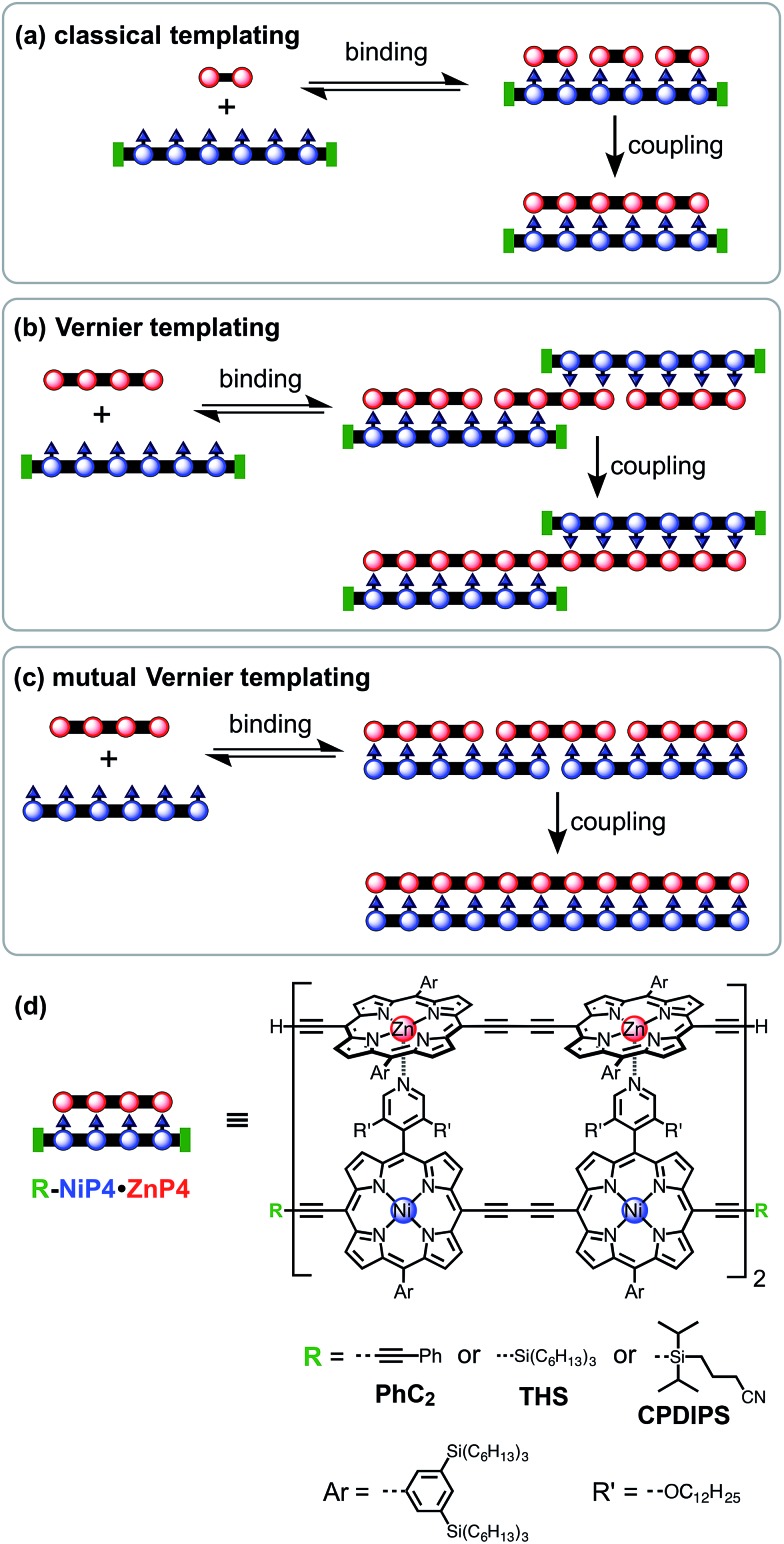
Linear template-directed syntheses of monodisperse linear porphyrin oligomers. (a) Classical template-directed synthesis. (b) Vernier templating synthesis. (c) Mutual Vernier templating: both strands couple. (d) Key to molecular design.

Three types of linear templating reactions were investigated in this study:

*(a) Classical templating*: A template binds to *n* molecules of a complementary substrate to form a double-strand 1 : *n* complex. After coupling, the product will be the same length as the template (Fig. 1a).

*(b) Vernier templating*: This strategy uses the mismatch between binding sites of substrate and template to direct the formation of the product with the lowest common multiple of the number of binding sites in the substrate and the template (Fig. 1b).

*(c) Mutual Vernier templating*: This is similar to Vernier templating, except that now both components undergo coupling, so that two product stands are formed, each controlled by the other (Fig. 1c).

### Molecular design

Most previous examples of linear template-directed oligomerization^24^ have used DNA templates,^25–27^ or have been based on the hydrogen-bonding of DNA nucleobases,^28,29^ whereas the recognition motif used in this work is the coordination of ligands to metalloporphyrins. We have investigated the Glaser–Hay oxidative homocoupling of alkyne-terminated porphyrin oligomers, as shown in Fig. 1d. Two types of porphyrins were used: (i) zinc porphyrins, which bind axial ligands such as pyridine, and (ii) porphyrins bearing *meso*-4-pyridyl substituents that can coordinate to zinc sites. Nickel(ii) was inserted into the *meso*-pyridyl porphyrins to prevent accidental metallation during coupling reactions; nickel(ii) porphyrins were chosen because they have a negligible affinity for axial ligands (except when the porphyrin has electron-withdrawing substituents^30^).^31^ The ends of the template-strand were capped with phenyl acetylene (**PhC_2_**), (3-cyanopropyl)diisopropylsilyl (**CPDIPS**)^32^ or trihexylsilyl (**THS**) groups, except in the case of mutual templating when both strands have reactive terminal alkynes. Two types of substituents were used to enhance solubility and avoid aggregation: 3,5-bis(trihexylsilyl)phenyl groups on the porphyrin *meso*-positions and dodecyloxy chains on the pyridyl groups. The synthesis of these components builds on previously published work^33^ and is presented in ESI.† Related *meso*-(4-pyridyl)porphyrin oligomers have previously been investigated for the self-assembly of box-shaped architectures,^34,35^ and double-strand porphyrin arrays have been assembled using similar strategies.^36^

### Coupling conditions

Glaser–Hay coupling is normally carried out using copper(i) chloride and tetramethylethylenediamine (TMEDA) with air or O_2_ as the oxidant, in a dry solvent, dichloromethane or acetone, with vigorous stirring at room temperature.^37,38^ However, coordination of TMEDA to zinc porphyrins can compete with binding to a template. UV-vis titrations of the zinc porphyrin monomer with TMEDA showed that the binding constant is *K*_f_(TMEDA) = (1.9 ± 0.2) × 10^4^ M^–1^, at 298 K in dichloromethane, which is almost as strong as that of pyridine under the same conditions; *K*_py_ = (2.8 ± 0.2) × 10^4^ M^–1^ (see the detail in ESI†). 2,2′-Bipyridine (2,2′-BiPy) can also act as a ligand in Glaser–Hay coupling^38^ and it has a much lower affinity for zinc porphyrins, *K*_f_(2,2′-BiPy) = 5.6 ± 0.3 M^–1^ (see the detail in ESI†). In this work, we used 2,2′-BiPy rather than TMEDA to maximize the binding to the template. CuCl/2,2′-BiPy gives a slower coupling reaction than CuCl/TMEDA, which is also beneficial in the template-directed synthesis of linear oligomers, because it is necessary to stop the reactions before they go to completion to avoid polymerization.

## Results and discussion

### Characterization of double-strand complexes

**Fig. 2:**
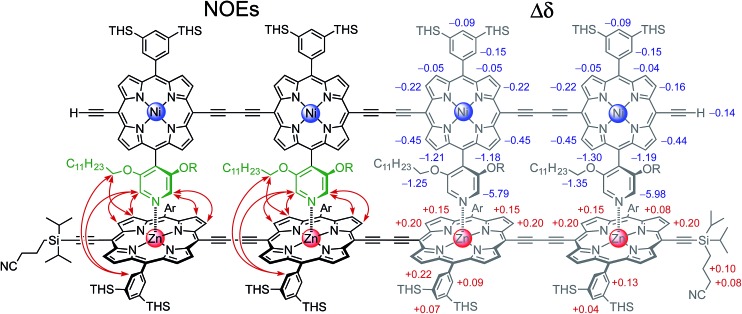
The chemical structure of the 4-rung ladder complex **NiP4·CPDIPS-ZnP4** (CDCl_3_, 500 MHz, 298 K) together with key NOEs between the components (left) and complexation-induced changes in proton chemical shift.

The 2- and 4-rung ladder complexes (**NiP2·CPDIPS-ZnP2** and **NiP4·CPDIPS-ZnP4**) were prepared by ^1^H NMR titration. The equilibria for formation of both double-strand complexes are very strong, as reflected by the sharp NMR spectra of the complexes, and by the detection of complexes by MALDI-ToF mass spectrometry (see ESI†). The ^1^H NMR spectra of the complexes were fully assigned by 2D techniques (COSY and NOESY). Fig. 2 (left) shows the key NOEs between the strands of the 4-rung ladder complex (see ESI† for full details). The right hand side of Fig. 2 shows the changes in chemical shift on complexation (Δ*δ* = *δ*_bound_ – *δ*_free_). The corresponding data for 2-rung ladder complex are presented in the ESI.† The NOEs and changes in chemical shift confirm the geometry of the double-strand complexes. The Ni-strand lies within the shielding region of the porphyrin aromatic ring-currents of the Zn-strand, causing protons of the Ni-strand to resonate at lower chemical shift, by an amount which depends on the distance from the Zn-strand. On the other hand, the ring current of the Ni-strand deshields the protons of the Zn-strand.

#### Stability of ladder complexes and UV-vis-NIR titrations

**Fig. 3:**
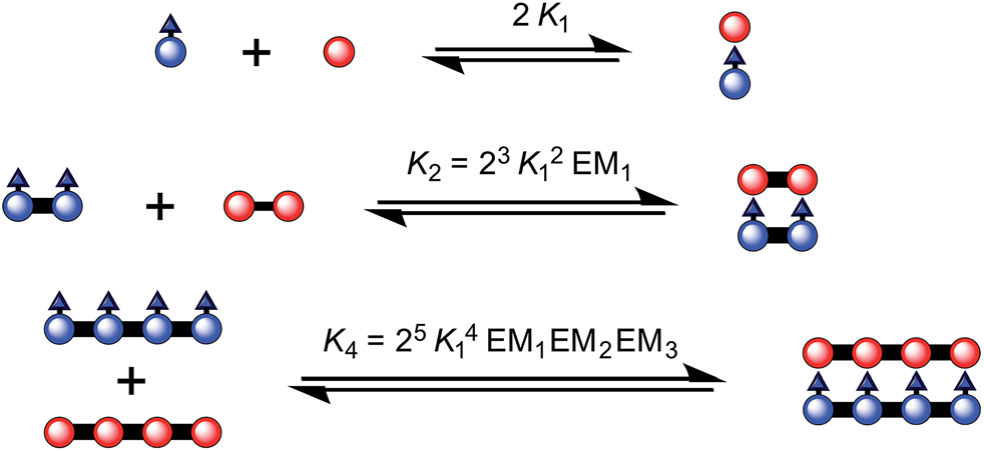
The ladder complexes and calculation of effective molarities from binding constants. The statistical factors used here, in Fig. 5 and Table 1 were calculated from the symmetry numbers as explained in ref. 39 and 40.

**Fig. 4:**
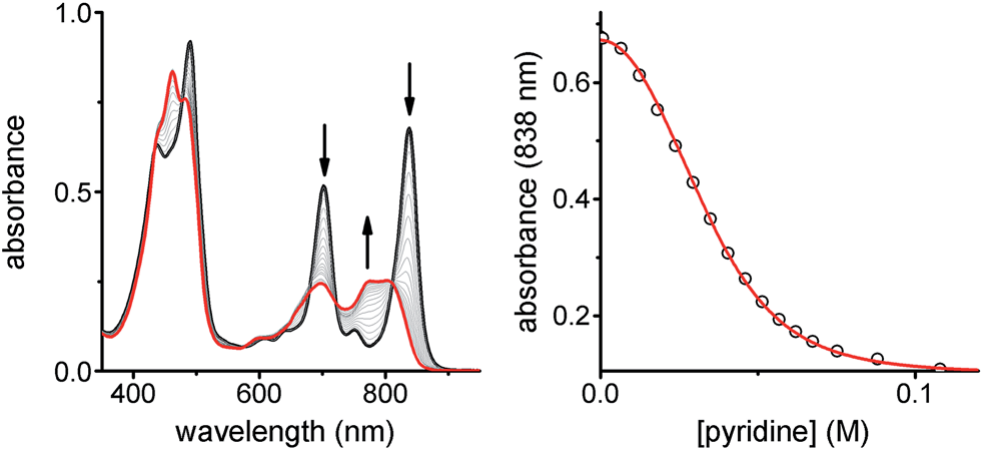
UV-vis-NIR denaturation titration of **CPDIPS-ZnP4·NiP4** ([complex] = 1.11 × 10^–6^ M) with pyridine in dry CH_2_Cl_2_ at 298 K. (left) Changes in absorption upon addition of pyridine; the spectrum of the initial complex (thick black line) and the final spectra of the pyridine-saturated Zn tetramer and unbound Ni tetramer (red line). Arrows indicate areas of increasing and decreasing absorption during the titration; (right) binding isotherm (open dots) derived from absorption data at *λ* = 838 nm and calculated isotherm (red line) (Run 1: *R* ^2^ = 0.9997; Run 2: *R* ^2^ = 0.9997).

The binding strength of the 4-rung ladder complex between **NiP4** and **CPDIPS-ZnP4** was studied to provide a measure of the stability and cooperativity of ladder formation (Fig. 3).^39,40^ The binding constant of the 2-rung ladder complex **NiP2·CPDIPS-ZnP2** was also elucidated as a reference (see ESI†). The UV-vis-NIR formation titration was performed at the constant micromolar concentration of **CPDIPS-ZnP4** in dry dichloromethane at 25 °C. Upon addition of **NiP4**, the Q-band absorption of **CPDIPS-ZnP4** red shifts and is sharpened as a consequence of coplanarization of the porphyrin units, leading to stronger electronic coupling among the units.^33,41^ The binding isotherm is essentially linear (see ESI† for the formation titration) with an abrupt end point after addition of 1 equivalent of the ligand **NiP4**, consistent with the 1 : 1 stoichiometry of the ladder complex **NiP4·CPDIPS-ZnP4**. The stability constant is too strong to determine directly from the formation curve but it can be evaluated indirectly by denaturation. Under the same conditions as the formation titration, a large excess of pyridine was titrated into the 4-rung ladder complex to displace the tetradentate ligand **NiP4**. The denaturation titration shows that the absorption spectrum of the complex **NiP4·CPDIPS-ZnP4** is red-shifted and sharper compared to that of the pyridine-bound **CPDIPS-ZnP4** complex formed at the end of the titration, due to the more rigid structure and coplanarization between porphyrin units in the ladder complex (Fig. 4).^42^ Observation of several isosbestic points in the denaturation titration and the sigmoidal binding isotherm indicate an all-or-nothing two-state equilibrium (*i.e.*, that partially denatured species are not populated) (Fig. 4 left).^40,43^ The simple two-state model gives a perfect fit to the denaturation data, resulting in *K*_dn_ = 0.39 ± 0.06 M^–3^. This value can be used to calculate the stability constant of the 1 : 1 ladder complex by considering a thermodynamic cycle, giving *K*_4_ = (1.8 ± 0.6) × 10^18^ M^–1^ (see details in ESI†). The complementarity between two strands for the 4-rung ladder complex **NiP4·CPDIPS-ZnP4** can be expressed as an effective molarity by comparison with the 2-rung ladder complex **NiP2·CPDIPS-ZnP2**.^40^ According to the equations in Fig. 3, the equilibrium constants, *K*_2_ and *K*_4_ for the formation of the 2- and 4-rung ladder complexes imply that the effective molarities are EM_1_ = 0.3 ± 0.1 M and EM_2_EM_3_ = 4.3 ± 1.5 M^2^, respectively (Table 1). This increase in effective molarity implies that binding becomes significantly more cooperative in the 4-rung ladder. The low EM in the 2-rung ladder reflects its flexibility. The higher cooperativity in the 4-rung ladder can be expected to lead to higher yield in the template-directed synthesis of linear porphyrin tetramer.

**Table 1:** Statistically corrected stability constants *K*_1_, *K*_2_ and *K*_3_, as defined in Fig. 3, measured in CH_2_Cl_2_ at 298 K for pyridine + **CPDIPS-ZnP1** (*K*_1_), **NiP2** + **CPDIPS-ZnP2** (*K*_2_) and **NiP4** + **CPDIPS-ZnP4** (*K*_4_). These values of *K*_1_, *K*_2_ and *K*_3_ were used to calculate the values of EM_1_, (EM_2_EM_3_)^0.5^, *β*_1_ _:_ _1_, *β*_1_ _:_ _2_, *β*_2_ _:_ _2_ and *β*_2_ _:_ _3_ as explained in Fig. 3 and 5

*K*_1_	(1.4 ± 0.1) × 10^4^ M^–1^
*K*_2_	(5.0 ± 0.7) × 10^8^ M^–1^
*K*_4_	(1.8 ± 0.6) × 10^18^ M^–1^
EM_1_	0.3 ± 0.1 M
(EM_2_EM_3_)^0.5^	2.1 ± 0.4 M
*β*_1_ _:_ _1_	(5.4 ± 1.6) × 10^18^ M^–1^
*β*_1_ _:_ _2_	(1.8 ± 0.8) × 10^27^ M^–2^
*β*_2_ _:_ _2_	(8.9 ± 5.2) × 10^35^ M^–3^
*β*_2_ _:_ _3_	(8.1 ± 7.1) × 10^53^ M^–4^

#### Stability of Vernier complexes

**Fig. 5:**
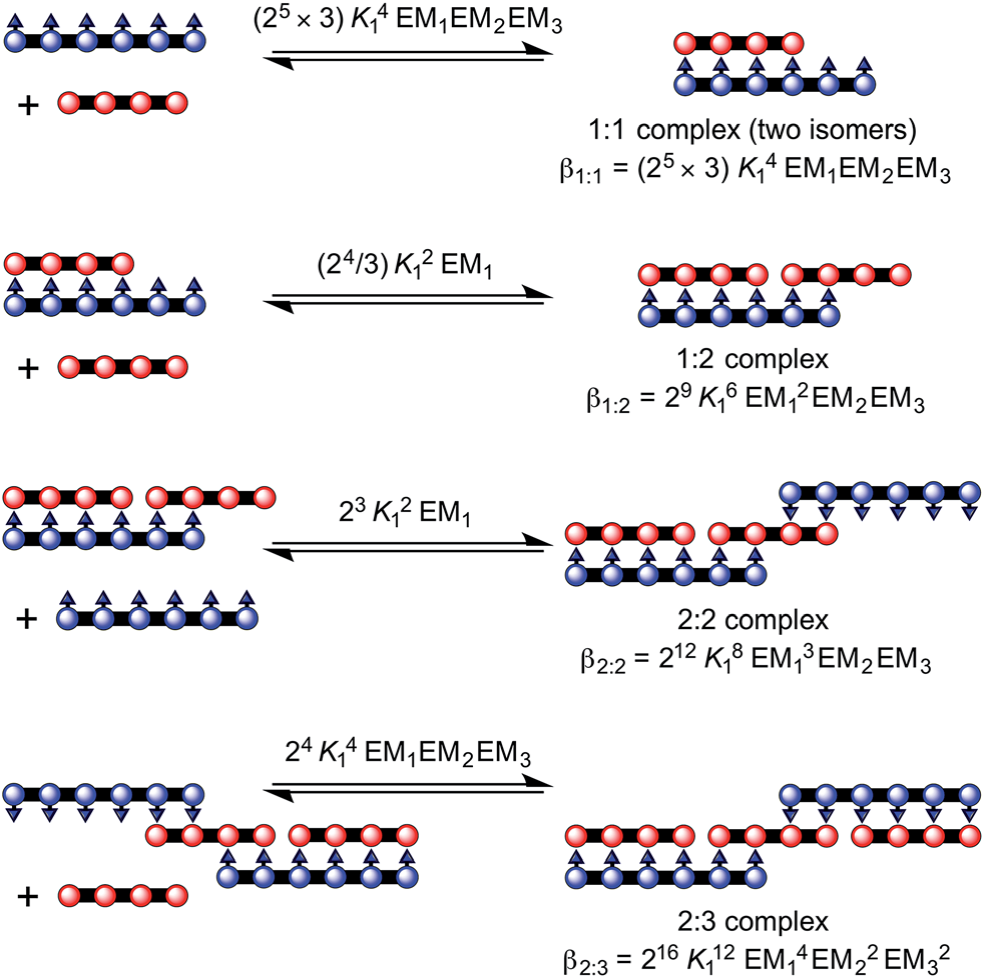
Complex formed between **ZnP4** and **NiP6**. The overall stability constants for the formation of each complex are provided in terms of *K*_1_ _:_ _2_, *K*_2_ _:_ _3_ and *K*_1_ _:_ _1_.

**Fig. 6:**
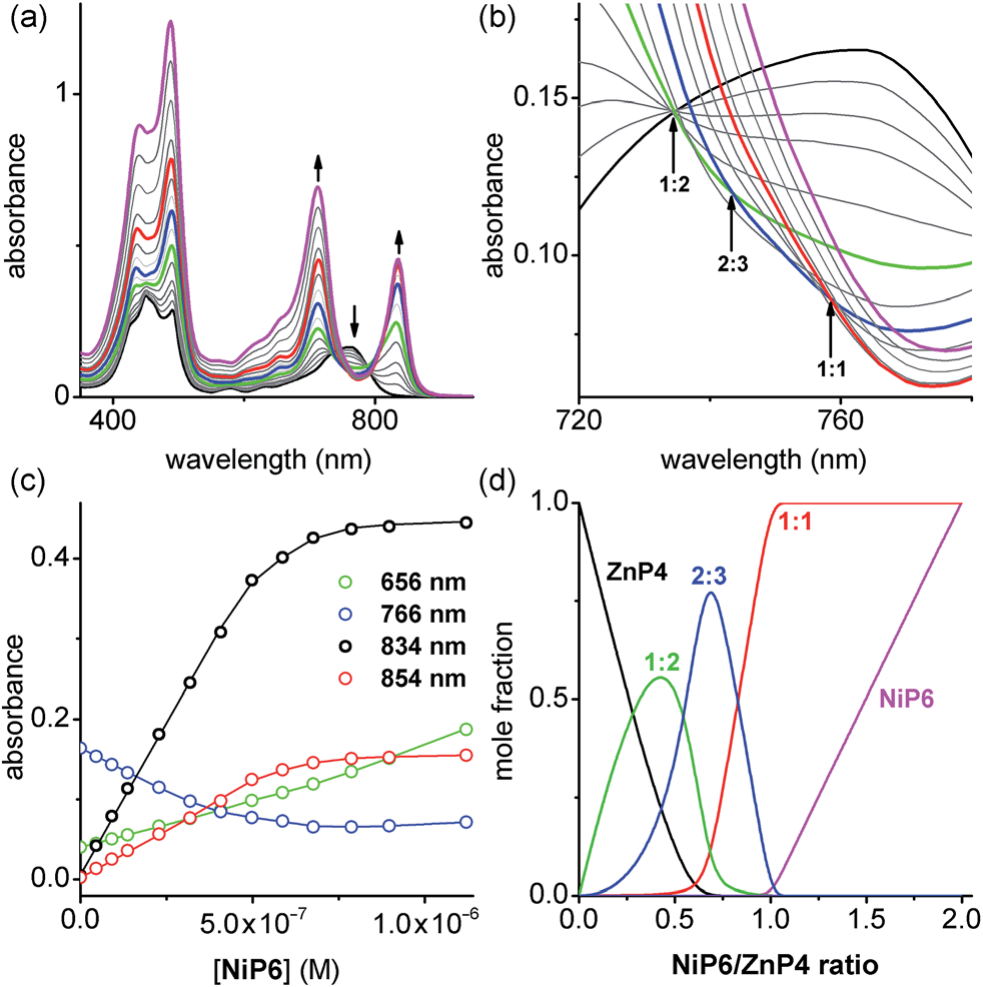
(a) UV-vis-NIR titration of **ZnP4** ([**ZnP4**] = 0.73 μM) with **NiP6** in dry CH_2_Cl_2_ at 298 K. Arrows indicate increasing or decreasing absorption (black, free **ZnP4**; green, 1 : 2 complex; blue, 2 : 3 complex, red, 1 : 1 complex and magenta, the end spectrum of **NiP6**-saturated-**ZnP4**). (b) Zoom-in of the change of isosbestic points in the absorption spectra. Arrows point out three subsequent isosbestic points at 0.5, 0.7 and 1.0 equivalents of **NiP6**, which is consistent with **NiP6** : **ZnP4** complexes of 1 : 2, 2 : 3 and 1 : 1 stoichiometries, respectively. (c) Binding isotherm (open dots) derived from absorption data at various wavelengths and fit obtained from global analysis with SPECFIT (solid lines). (d) The speciation plots as a function of the ratio [**NiP6**]/[**ZnP4**] (black, free **ZnP4**; green, 1 : 1 complex; blue, 2 : 3 complex, red, 1 : 1 complex and magenta, free **NiP6**).

In order to test the validity of the Vernier template synthesis of larger porphyrin oligomers, we investigated the ability of Zn and Ni porphyrin oligomers to form Vernier complexes by studying the stability of the 2 : 3 complex between a Ni hexamer and a Zn tetramer, (**NiP6**)_2_·(**ZnP4**)_3_ (Fig. 5). The overall binding constant (*β*_*N*_ _:_ _*N*_) of each complex can be predicted from the previously measured values *K*_1_, EM_1_ and EM_2_EM_3_ (Fig. 5) as listed in Table 1. The formation of the Vernier complex was probed by UV-vis-NIR titration of **ZnP4** with **NiP6**, at a constant concentration of **ZnP4** (*ca.* 10^–6^ M) in dry dichloromethane at 25 °C (Fig. 6). When increasing amounts of the **NiP6** were added, the intensity of the Q-band absorption of free **ZnP4** at 764 nm decreased, and two new sharper Q-bands appeared at the blue- and red-shifted wavelengths of 712 nm and 836 nm corresponding to two bound porphyrin species, *i.e.* bound **NiP6** at 712 nm and bound **ZnP4** at 836 nm (Fig. 6a). Observation of three subsequent isosbestic points at 0.5, 0.7 and 1.0 equivalents of **NiP6** indicates formation of a series of self-assembled complexes with **NiP6** : **ZnP4** stoichiometries of 1 : 2, 2 : 3 and 1 : 1 as illustrated in Fig. 6b. The titration data were analyzed by SPECFIT in term of six colored species as predicted in Fig. 5, **NiP6**, **ZnP4**, 1 : 1, 1 : 2, 2 : 2 and 2 : 3 complexes. The predicted *β*_*N*_ _:_ _*N*_ values as listed in Table 1 were used to fit to the experimental data by SPECFIT, giving an excellent fit to this model as shown in Fig. 6c. The corresponding speciation profile (Fig. 6d) shows that the intermediate 1 : 2 assembly appears before the 2 : 3 assembly becomes the major species (up to 75%) present at the appropriate stoichiometry. The 2 : 3 Vernier complex then dissociates in the presence of **NiP6**. This binding study confirms that the Vernier complex is the dominant species in solution at the 2 : 3 stoichiometry.

### Classical template-directed synthesis

Initial experiments were carried out to test the ability of a porphyrin tetramer to act as a template for coupling together two porphyrin dimers. Both Ni and Zn porphyrin tetramer templates were explored for these reactions. Subsequently, these studies were extended to use a porphyrin hexamer as a template.

#### Zn tetramer as the template (2 × P2 ⇔ P4)

**Fig. 7:**
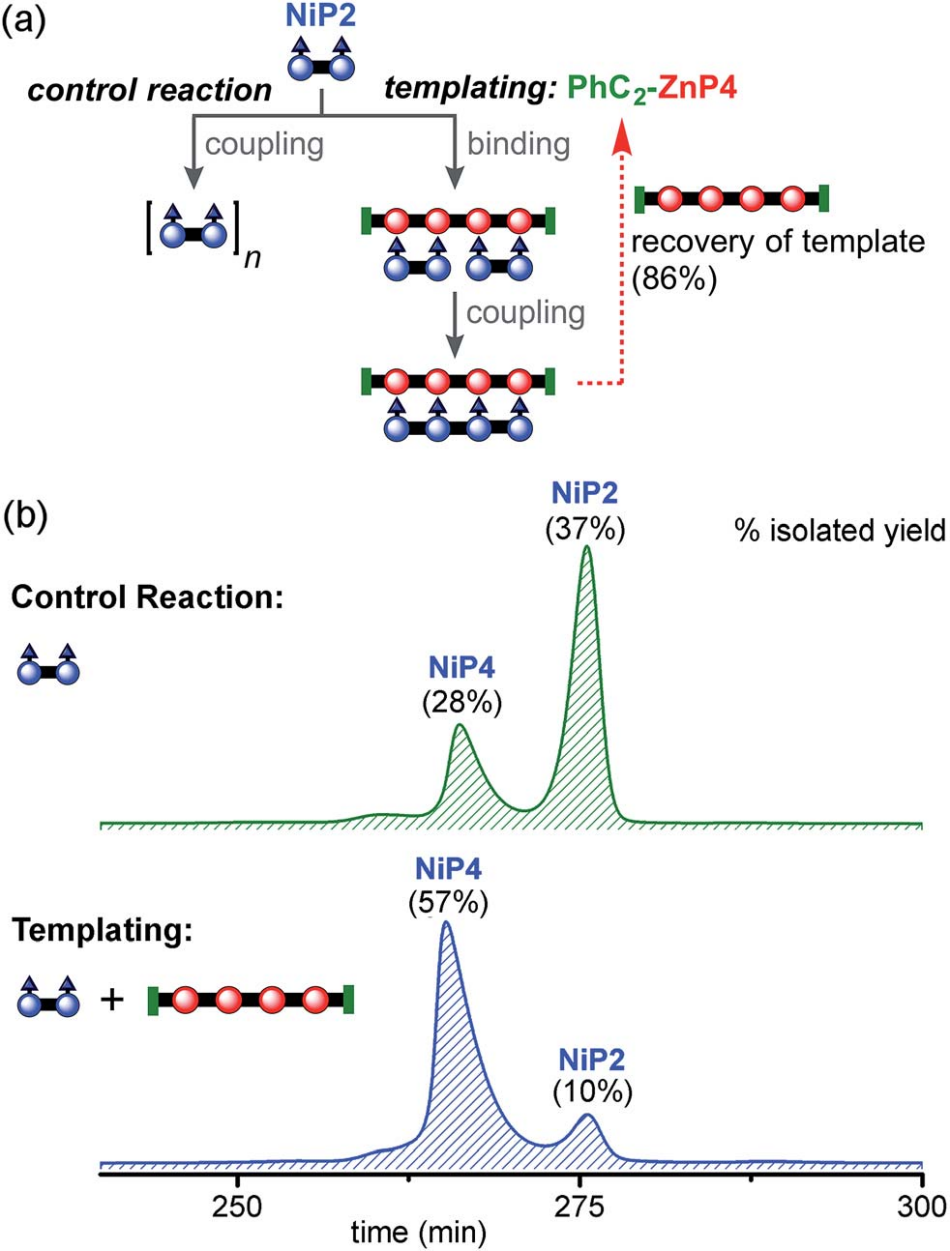
Reaction design and outcome of linear tetramer templating for the synthesis of **NiP4** from **NiP2**, using **PhC_2_-ZnP4** as the template. (a) Reaction scheme. (b) GPC traces (detection at 646 nm, toluene/1% pyridine) of the non-templated reaction (top) and the templated reaction (bottom) product mixture after removal of the template **PhC_2_-ZnP4**. The product **NiP4** and unreacted starting material **NiP2** were isolated and identified by MALDI-ToF MS and ^1^H NMR spectroscopy.

The **NiP2** dimer was used as a substrate with **PhC_2_-ZnP4** as the template. Initially, **NiP2** and **PhC_2_-ZnP4** were mixed in a 2 : 1 ratio in dichloromethane to form the ladder complex **(NiP2)_2_·(PhC_2_-ZnP4)**. UV-vis-NIR spectroscopy confirmed complex formation. Then the mixture was subjected to the CuCl/2,2′-BiPy Glaser–Hay coupling. A control reaction was performed under identical conditions, in the absence of the **PhC_2_-ZnP4** template (Fig. 7a). The reactions were monitored by UV-vis-NIR spectroscopy and stopped after 2 h, by which time conversion had become slow (see detail in ESI†). The coupling reagents and template were easily separated from the nickel-porphyrin oligomers by passing through a short silica column, eluting with a gradient of 1 to 10% pyridine in chloroform. The template was re-isolated in 86% yield. The control reaction was worked up in the same way. The mixtures of **NiP*N*** oligomers were purified by gel permeation chromatography (GPC) in toluene/1% pyridine (Fig. 7b), and analyzed by MALDI-ToF and ^1^H NMR spectroscopy. Without the template, **NiP2** was converted to **NiP4** slowly in 28% isolated yield and the unreacted **NiP2** substrate was re-isolated in 37% yield. In the templated reaction, **NiP4** was isolated in 57% isolated yield and unreacted **NiP2** was re-isolated in 10% yield.

Unreacted **NiP2** starting material was observed in both these reactions, because they were stopped before completion to prevent the formation of long Ni oligomers, which would be inevitable at high extents of coupling. It is surprising that traces of longer Ni porphyrin oligomers were not observed by GPC in these reaction mixtures; this may reflect the slowness of the coupling reaction; it is also possible that longer oligomers were lost during work-up due to low solubility. We also carried out similar reactions using **CPDIPS-ZnP4** as the template instead of **PhC_2_-ZnP4**. As expected, changing the capping group does not alter the effectiveness of the template. The desired product **NiP4** was isolated in yields ranging from 35% (from 39 mg of **NiP2**), 42% (from 3.0 mg of **NiP2**) to 54% (from 22.3 mg of **NiP2**), all using the **CPDIPS-ZnP4** template.

#### Ni tetramer as the template (2 × P2 ⇔ P4)

**Fig. 8:**
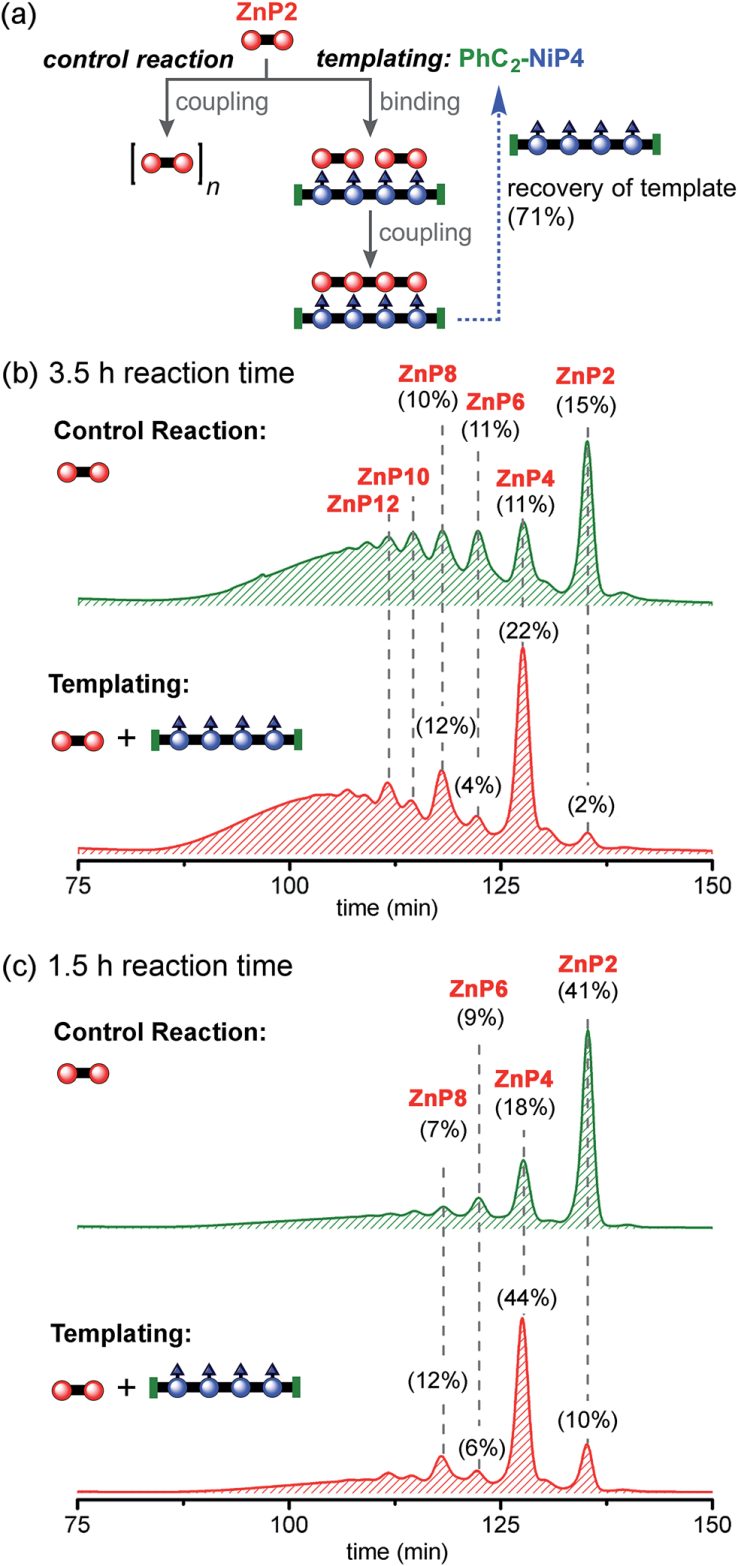
Reaction design and outcome of linear tetramer templating for the synthesis of **ZnP4** from **ZnP2** using **PhC_2_-NiP4** as a template. (a) Reaction scheme. (b) GPC traces (detection at 591 nm, toluene/1% pyridine) of the non-templated reaction (top) and the linear templated reaction (bottom) product mixture after removal of the template **PhC_2_-NiP4** at 3.5 h and (c) at 1.5 h. Products were identified by analytical GPC based on calibrated retention times, MALDI-ToF and ^1^H-NMR for **ZnP2** and **ZnP4**. **ZnP4** was isolated in 23% and 30% yield from the reaction time at 3.5 and 1.5 h, respectively. The percentages in GPC traces represent the % GPC yields of corresponding compounds, calculated by integration of the peak areas.

The reactions discussed in the previous section provided a good supply of **NiP4**, which was capped with phenylacetylene to give **PhC_2_-NiP4** then used as a template for preparing **ZnP4** from **ZnP2**. Thus Zn and Ni porphyrins can swap their roles. The templated and non-templated control reactions were performed as summarized in Fig. 8a. The reactions were stopped after 3.5 h, since UV-vis-NIR spectroscopy indicated formation of longer Zn porphyrin polymers after this time (see detail in ESI†). The coupling reagents were removed using a short silica column eluted with a chloroform/1–10% pyridine gradient, and the template was re-isolated in 71% yield. The product distribution was analyzed by GPC (toluene/1% pyridine) (Fig. 8b). The template accelerates the coupling reaction, consuming almost all starting material (98% conversion by GPC) and providing the desired product **ZnP4** in 23% isolated yield (22% GPC analytical yield). Besides the expected product **ZnP4**, longer oligomers were formed with lengths in multiples of four units (**ZnP8** and **ZnP12**). The formation of multiple-of-four products can be attributed to the further coupling of free acetylenic end groups subsequent to formation of the desired product **ZnP4**. With no template, the reaction yields a product distribution characteristic of statistical polymerization and shows more of the unreacted substrate **ZnP2** (15% GPC yield compared with 2% in the presence of the template).

Reducing the reaction time from 3.5 h to 1.5 h reduced the formation of longer oligomeric byproducts, while increasing the yield of the desired product **ZnP4** (Fig. 8c). In the presence of the template, the reaction gave **ZnP4** in up to 30% isolated yield (44% GPC yield), together with enhanced amounts of multiple-of-four byproducts (**ZnP8** and **ZnP12**). The template acts as a positive template in this reaction,^23^ promoting the intramolecular coupling leading to the formation of the desired tetramer from dimers. Surprisingly, under these reaction conditions, zinc porphyrins undergo faster Glaser coupling than nickel porphyrins, despite the fact that nickel is more electron-withdrawing than zinc, which would have been expected to favor the Glaser coupling reactivity.^37b,44^

#### Hexamer template-directed synthesis (3 × P2 ⇔ P6)

**Fig. 9:**
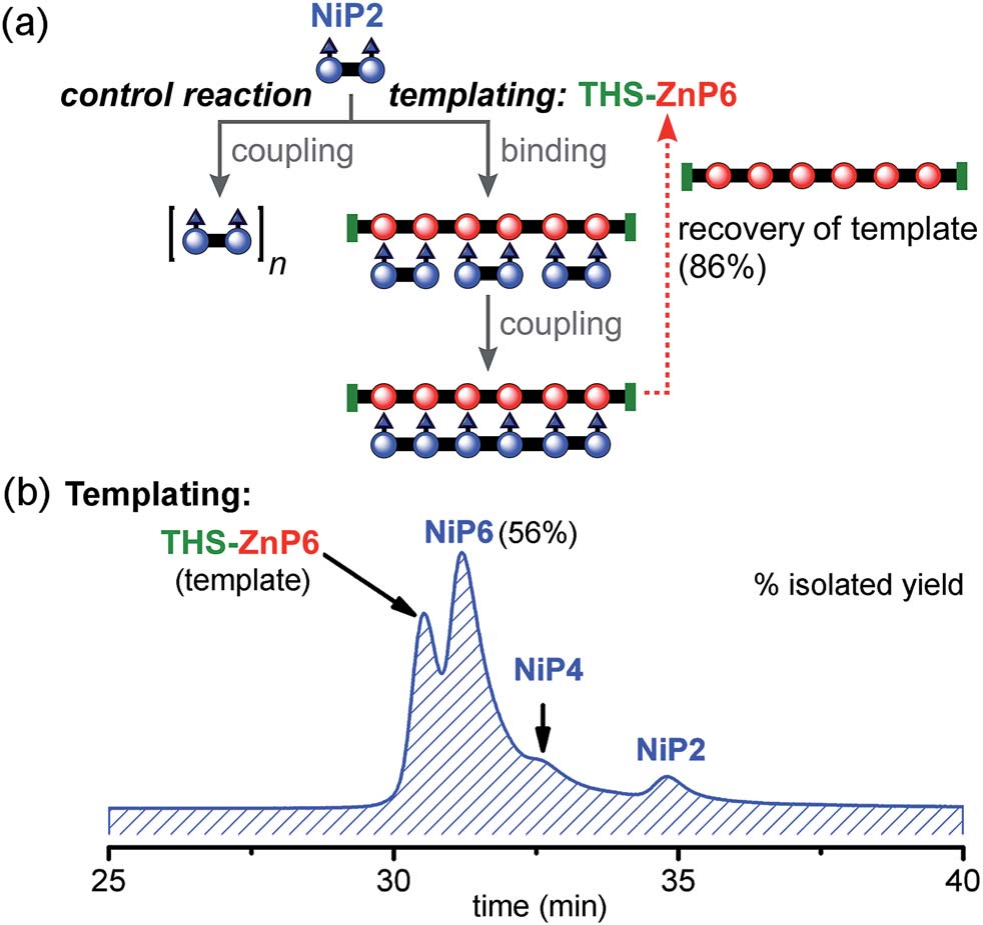
Reaction design and outcome of linear tetramer templating for the synthesis of **NiP6** from **NiP2** using **THS-ZnP6** as a template. (a) Reaction scheme. (b) GPC traces (detection at 646 nm, THF) of the linear templating reaction product mixture with the template **THS-ZnP6**. Products were identified by MLALDI-ToF and ^1^H-NMR for **NiP6**.

Encouraged by the results presented above, we extended the limits of linear templating by testing the hexamer **THS-ZnP6** as a template for coupling three molecules of **NiP2**. The templated and non-templated control reactions were set up as described above, with a stoichiometric ratio of **NiP2** to **THS-ZnP6** of 3 : 1 (Fig. 9). Under identical reaction conditions, the control reaction with no template gave negligible amounts of **NiP6** as previously shown in Fig. 7b. With this longer template, we isolated the desired product **NiP6** in a 56% yield (5 mg from 9 mg of **NiP2**) and the template was re-isolated in 86% yield (Fig. 9b). The reaction was scaled up to 20 mg of **NiP2** substrate giving the desired product **NiP6** in 63% isolated yield (13 mg). This practical route to **NiP6** was used to prepare the starting materials required for the Vernier templating and mutual Vernier templating experiments described below.

### Vernier template synthesis (3 × P4 ⇔ 2 × P6)

**Fig. 10:**
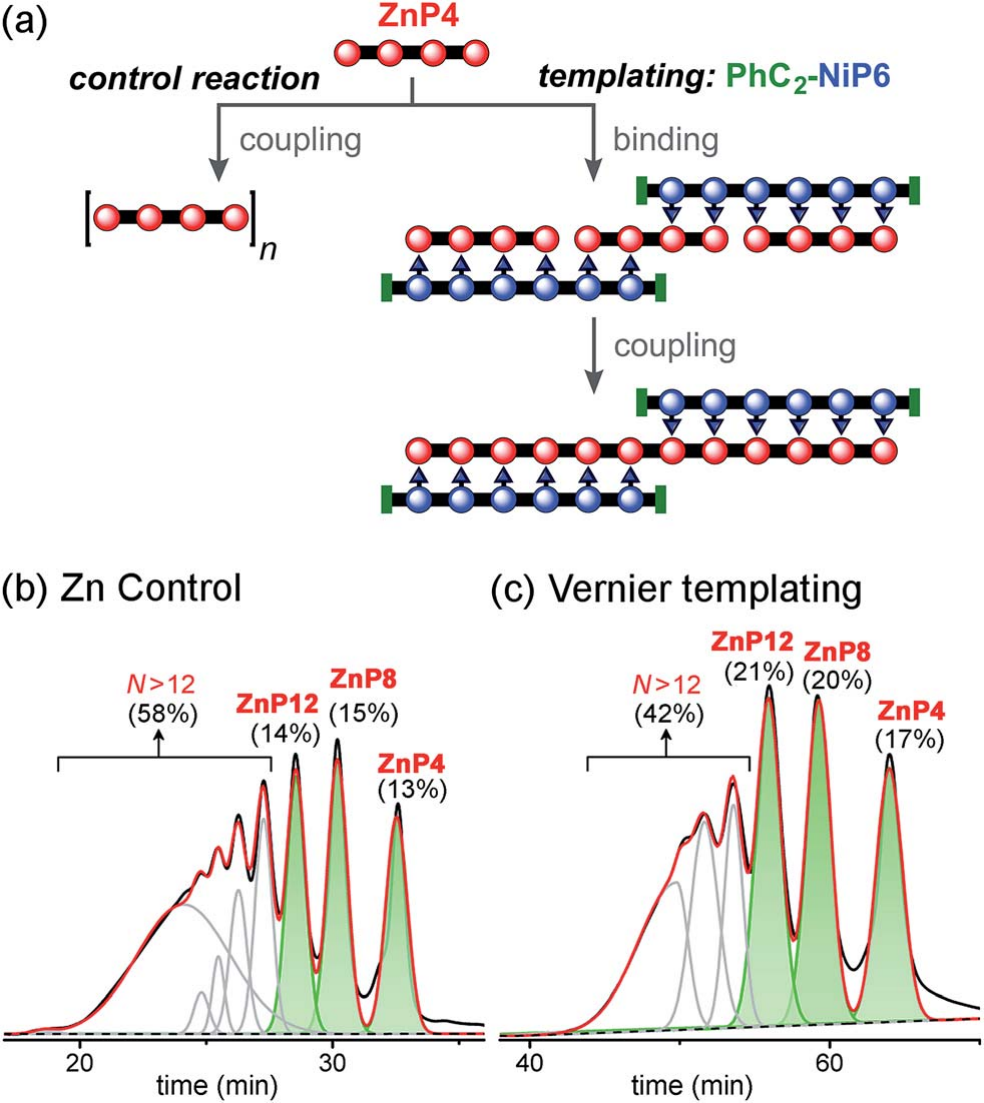
Reaction design and outcome of linear Vernier templating for the synthesis of **ZnP12** from **ZnP4** using **PhC_2_-NiP6** as a template. (a) Reaction scheme. (b) GPC traces (detection at 591 nm, toluene/1% pyridine) of the non-templated reaction and (c) the templated reaction product mixture after removal of the template **PhC_2_-NiP6** at 4 h. Products were identified by analytical GPC based on calibrated retention times.^19^ The percentages in GPC traces are the analytical yields of each oligomer, from integration of the peaks (absorption at 591 nm).

Vernier templating was tested using **PhC_2_-NiP6** as a template and **ZnP4** as a substrate to synthesize the zinc porphyrin dodecamer **ZnP12** (Fig. 10). The capped template **PhC_2_-NiP6** was prepared by reacting **NiP6** with excess phenylacetylene (see details in ESI†). The templated reaction was set up as described above: **ZnP4** and **PhC_2_-NiP6** were mixed in a 3 : 2 stoichiometric ratio in dry dichloromethane and complexation was confirmed by UV-vis-NIR spectroscopy, then CuCl and 2,2′-BiPy were added. The reaction was stopped after 4 h (when UV-vis-NIR spectroscopy indicated that conversion became slow; see detail in ESI†). After removal of the coupling reagents and template, the products were identified by analytical GPC (toluene/1% pyridine) based on calibrated retention times.^19^

With no template (Fig. 10b), the reaction produced more polymers and small amount of the desired product **ZnP12** (14% analytical yield) as noticed in the previous control experiment for Ni tetramer templated reaction (Fig. 8b). In the presence of template (Fig. 10c), the substrate **ZnP4** was converted into two major products: the intermediate **ZnP8** in 20% analytical yield and the Vernier product **ZnP12** in 21% analytical yield. The unreacted substrate **ZnP4** was observed in 17% analytical yield, indicating slower coupling in the Vernier templating reaction than the classical templating reaction. It appears that template-directed formation of **ZnP8** is faster than subsequent coupling to form **ZnP12**. The results show that **ZnP12** can be synthesized by the linear Vernier templating, even though there is competing polymerization. The yield of the templated reaction could probably be increased by optimizing the reaction time.

### Mutual Vernier template synthesis (3 × P4 ⇔ 2 × P6)

**Fig. 11:**
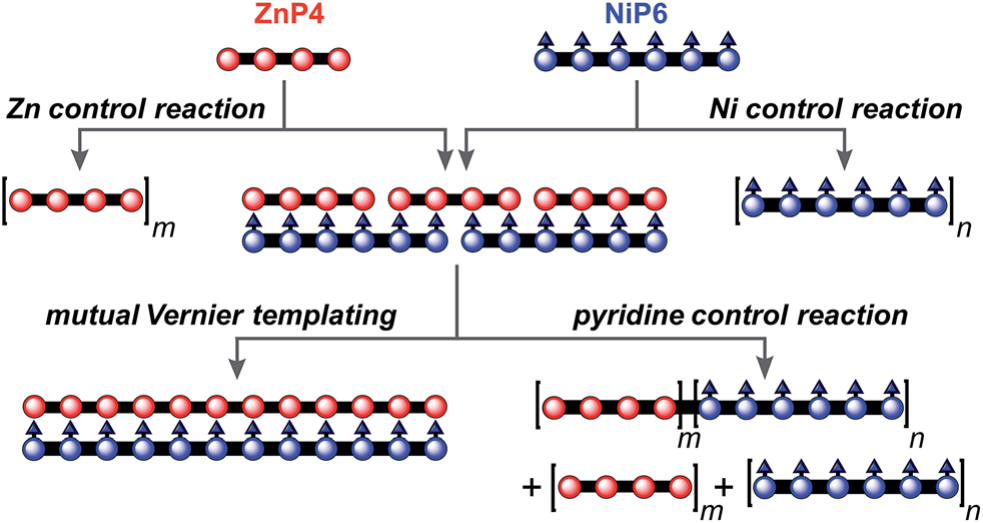
The mutual Vernier templating and three control reactions for the synthesis of **NiP12** and **ZnP12**. *m* and *n* are the number of the repeat units.

The result from the trial Vernier templating reaction and the binding study of the Vernier complex encouraged us to try the mutual Vernier template-directed synthesis of two different types of linear metalloporphyrin 12-mers using two non-commensurate substrates **ZnP4** and **NiP6**. When both substrates form the Vernier ladder complex, under coupling conditions the Zn and Ni-strand can serve as templates for each other in order to direct the construction of **ZnP12** and **NiP12** as expected from the lowest common multiple of the number of binding units of **ZnP4** and **NiP6** (Fig. 11). It is not necessary for both strands to couple at the same time because after one strand couples, the Vernier product can act as a template for the other strand. However the danger with mutual Vernier templating is that there are many potential reaction pathways, and that byproducts such as longer oligomers and hetero-coupled strands can act as templates for competing pathways.

Similarly to the previous experiment, **ZnP4** and **NiP6** were mixed in a 3 : 2 stoichiometric ratio in dry dichloromethane and the complexation was confirmed by UV-vis-NIR spectroscopy, then aliquots of the coupling reagents CuCl and 2,2′-BiPy were added every 3 h to maintain the coupling reaction. The reaction was monitored by analyzing aliquots by GPC (eluting with 1% pyridine/THF) at 1, 3, 4, 6, 7 and 8 h. After 8 h, the reaction was further analyzed by MALDI-ToF mass spectrometry.

Three control reactions were set up as follows (Fig. 11):

#### Zn control

This reaction was prepared in a similar approach to the Vernier-mutual templating reaction but in the absence of **NiP6**, to compare the statistical homocoupling of **ZnP4** alone.

#### Ni control

This reaction was set up similarly to the mutual Vernier templating reaction but in the absence of **ZnP4** in order to test the statistical homocoupling of **NiP6** with no template.

#### Pyridine control

This reaction was set up in identical condition to the mutual Vernier templating reaction but in the presence of 10% pyridine (by volume of solvent) to prevent the formation of the Vernier complex (**ZnP4**)_3_·(**NiP6**)_2_. As a result, the coupling reaction between **ZnP4** and **NiP6** should randomly produce the homocoupling product from Ni and Zn species and heterocoupling between Ni and Zn species in the presence of pyridine.

**Fig. 12:**
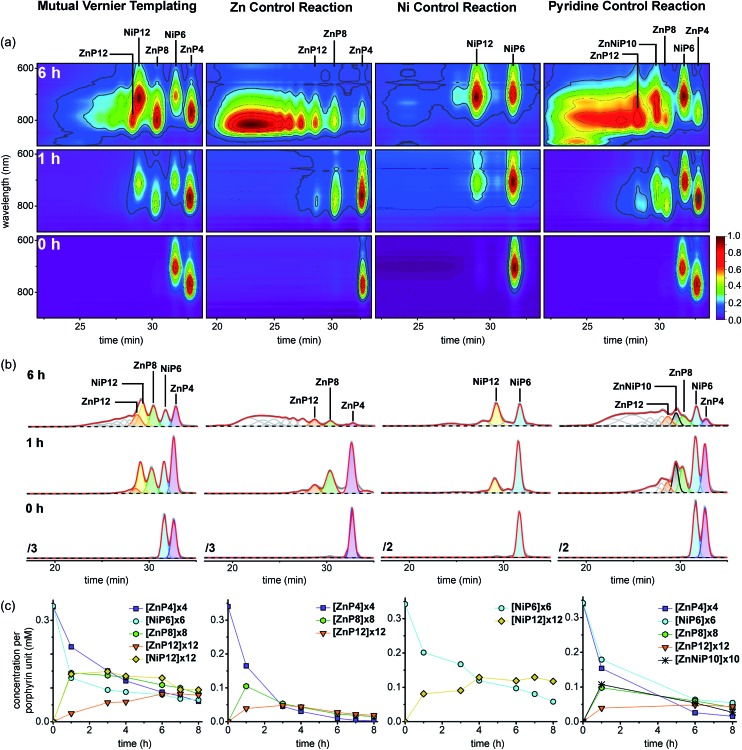
The outcome of the mutual Vernier templating and three control reactions of the synthesis of **NiP12** and **ZnP12**. (a) The selected deconvoluted GPC traces fitted to Gaussians by Origin® at 0, 1, 4, 6 and 8 h, respectively (THF/1% pyridine, detection at 479 nm where all species have essentially the same absorption coefficient per porphyrin unit). (b) The plots of analytical yields that were calculated by peak fitting of resolved GPC peak areas. (c) The selected 2D-plots at 0, 1, 4, 6 and 8 h, respectively. The intensity was normalized by rainbow colors from purple (lowest) to red (highest). The retention times of all components were calibrated from known compounds, and their identification was based on the calibrated retention time in GPC.

**Fig. 13:**
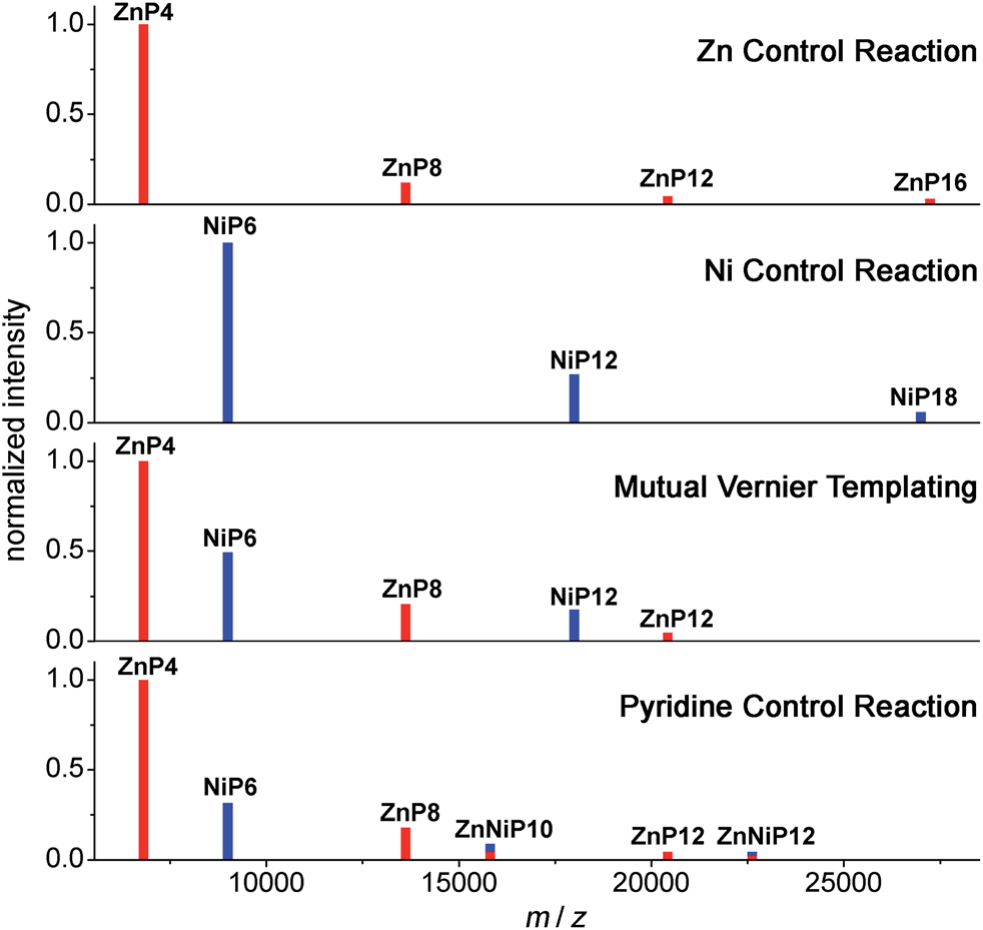
Schematic of MALDI-TOF spectra of the mutual Vernier templating and three control reactions of the synthesis of **NiP12** and **ZnP12**. Red lines represent the zinc porphyrin oligomers while blue lines indicate the nickel porphyrin oligomers. The mixed red-blue color indicates the heterocoupling zinc–nickel porphyrin oligomers. (See Fig. S34–S37† for raw mass spectra.)

The results from all reactions are presented in 2D-plots of the retention times *versus* Q-band absorption spectra, deconvoluted GPC traces and 1D-plots of analytical concentrations that were calculated by peak fitting of resolved GPC peak areas (Fig. 12), and MALDI-ToF mass analysis (Fig. 13), respectively, as follows.

#### 2D GPC-UV-vis plots

The 2D-plots of the retention times *versus* Q-band absorption spectra (Fig. 12a) allow the identification of the metalloporphyrins by the characteristic absorptions of Ni porphyrins at shorter wavelength than Zn porphyrins, even though the Ni and Zn porphyrin oligomers give overlapping peaks in the 1D GPC traces (Fig. 12b). In addition, we can see the growing formation of the products by monitoring the change in the Q-bands, yielding results consistent with the trends from the deconvoluted GPC traces, as discussed later.

At early reaction times (1 h) the mutual Vernier templating reaction shows a weak Q-band for **ZnP12** whereas **ZnP8** and **NiP12** are quite intense, but at later times the three products have similar intensities (Fig. 12a mutual Vernier templating). Although the retention times of **NiP12** and **ZnP12** are very close, their Q-bands can still be deconvoluted from the absorption wavelengths.

The Q-bands in the Zn control reaction become more red-shifted at shorter retention times as the reaction progresses, reflecting the formation of longer Zn oligomers (Fig. 12a Zn control) while the Q-bands in the Ni control show up at shorter wavelengths and are mostly unchanged, indicating slow conversion from **NiP6** to **NiP12** and small amount of polymers at shorter retention times (Fig. 12a Ni control).

Strikingly, in the pyridine control, in addition to the expected distinctive absorptions from Ni and Zn porphyrin oligomers, we can see the Q-band absorption in regions where both species absorb, implying the formation of the product **ZnNiP10** arising from heterocoupling between **ZnP4** and **NiP6** (Fig. 12a pyridine control).

#### Deconvoluted GPC traces and 1D-plots

Fig. 12b shows deconvoluted GPC traces of the Vernier-mutual templated and three control syntheses of **ZnP12** and **NiP12** from the absorption at 479 nm where all species have essentially the same absorption coefficient per porphyrin unit (see the absorption coefficient spectra in ESI†). All components were assigned based on their retention times, using a calibration scale based on the retention times of known compounds (see the detail of analytical GPC calibration in ESI†). Analytical yields were estimated from the areas of the GPC peak (Fig. 12b and c).

At the start of the linear mutual Vernier templating reaction (1 h), the Zn-strand accelerates the coupling reaction of **NiP6** yielding **NiP12** in 41%, whereas **ZnP12** forms only in 7% yield, and partial coupling from **ZnP4** to **ZnP8** (42%) was observed at the same yield as **NiP12**. It can be seen that the Zn-strand speeds up the coupling reaction of the Ni-strand as **NiP6** is consumed faster than in the Ni control (Fig. 12b and c mutual Vernier templating compared to Ni control). Surprisingly, the Ni-strand appears to slow down the coupling of the Zn-strand compared to the corresponding control (Fig. 12b and c mutual Vernier templating compared to Zn control). Then, while **ZnP8** is gradually converted to **ZnP12**, **NiP12** can further couple to form longer oligomers due to the absence of protecting groups at the acetylenic ends. After 8 h, the yields of the three products **ZnP8** (24%), **ZnP12** (23%) and **NiP12** (28%) became almost equivalent (Fig. 12a and b mutual Vernier templating). Both desired products **NiP12** and **ZnP12** can form longer oligomer while the coupling reagent is still active, providing traces of high-mass oligomers as seen in the GPC traces at longer reaction times (Fig. 12b mutual Vernier templating). The yields of the Vernier products appear to peak at a reaction time of 6 h (38% for **NiP12** and 24% for **ZnP12**).

As expected, the Zn control reaction led to the increasing statistical coupling of the Zn oligomer products as time proceeded, yielding polymers as the major products at the end of the reaction time (at 8 h). After at 1 h, the unreacted substrate **ZnP4** was dominant at 48%; **ZnP8** and **ZnP12** were also observed in 31% and 11% yield respectively. With increasing time from 3 to 8 h, the polymers become the major products and yields of **ZnP8** (from 16% to 4%) and **ZnP12** (from 14% to 6%) become very low. The highest yield of **ZnP12** (14%) is still much lower than from mutual Vernier templating (24%) (Fig. 12b and c Zn control).

On the other hand, the Ni control reaction gradually produced **NiP12** and a small trace of polymers due to the slow coupling rate of the Ni porphyrins. However without a template effect, the yield of **NiP12** in the beginning (24% at 1 h) is almost two times lower than from the Vernier templating (41%) and then increases to 34% at 8 h (Fig. 12a and b Ni control).

In the pyridine control (Fig. 12a and b pyridine control), homocoupling products **ZnP8** (from 28% to 12%) and **ZnP12** (from 12% to 13%) were statistically produced at almost the same rate as in the Zn control from 1 to 8 h. **NiP12** is not clearly observed in the GPC traces while the new species of the product of heterocoupling between **ZnP4** and **NiP6** was observed as anticipated, yielding the 10-porphyrin unit oligomer **ZnNiP10** in 29% and reducing to 7% yield from 1 to 8 h. After 8 h, polymers become the main products.

Since the retention time of **ZnP12** and **NiP12** are very close at 28.6 and 29.1 min in the Vernier-mutual templated reaction, there is uncertainty in the fitting area of the corresponding deconvoluted peaks. Thus, to confirm the reliability in the fitting results, yields of the product mixtures at 779 nm (where only Zn species absorb) were determined and compared indirectly by the product-to-substrate ratios of **ZnP12**/**ZnP4** and **ZnP8**/**ZnP4** to those at 479 nm, resulting in a good agreement of the values in both wavelengths as shown in ESI.†

To support the analyses obtained from calibrated GPC time-traces, the products of the Vernier-mutual templating and the control reactions were also analyzed by the MALDI-ToF mass spectrometry (Fig. 13). The mass data are in good agreement with the GPC analysis discussed above.

In comparison to the cyclic Vernier templating synthesis of 12-porphyrin nanoring using the Zn porphyrin tetramer substrate and radial hexapyridyl ligand template (T6),^17,18^ the linear Vernier-mutual templating approach shows that two distinctive species **NiP12** and **ZnP12** can be synthesized without a discrete template. Despite the fact that the unprotected end groups of the products **NiP12** and **ZnP12** can react further to produce polymeric mixtures in the linear route (a problem that does not arise in macrocyclization), the efficiency of the two methods are comparable in term of the range of yields (38% for **NiP12** and 24% for **ZnP12** compared to 39% for cyclic 12-mer^18^).

## Conclusions

We have demonstrated the first example of linear Vernier template-directed synthesis, and investigated a mutual Vernier template strategy, in which both strands of the Vernier complex undergo coupling.

Linear porphyrin oligomers of up to six porphyrin units were successfully synthesized by the classical template approach using complementary templates and substrates with commensurate lengths under oxidative-coupling conditions, in a manner reminiscent of DNA replication. The porphyrin templates in the classical approach can be recovered in high yield (up to 85%) making this method a practical route to the desired oligomers. However, classical templating is not suitable for the synthesis of longer linear oligomers due to the difficulty of synthesizing long templates. To solve this problem, we exploited linear mutual Vernier template-directed synthesis to achieve the construction of two complementary supramolecular porphyrin dodecamers, *via* the Vernier assembly of two different types of substrates. Mutual Vernier template coupling gives higher yields of the Vernier products than the control reactions, and it favors homo-coupling over cross-coupling (suppressing the formation of mixed Zn/Ni-strands). This approach adds to the toolbox for supramolecular strategies to the controlled synthesis of materials with interesting photophysical properties. In principle, the double-strand structures discussed here should be capable of autocatalytic self-replication, but replication will be strongly inhibited by the high stability of the duplex and the reaction profiles are expected to be parabolic rather than exponential.^45^ It is interesting to compare these ladder complexes with other designs for self-replicating molecules.^45–54^ There are also previous reports of synthetic systems in which double strand formation controls polymer growth.^55^ In the work presented here, we have confined our attention to templates in which all the sites are identical; in future it will be interesting to explore template-directed coupling of oligomers with sequences of Zn and pyridyl/Ni sites, for encoding binary information.^56^
