# Supplementary material for: Template-directed synthesis of linear porphyrin oligomers: classical, Vernier and mutual Vernier
Source: Chem Sci. 2017 Jan 20;8(4):2729–40. doi: 10.1039/c6sc05355f (PMC5426366; doi:10.1039/c6sc05355f)
Supplement: Supplementary file 1 [file SC-008-C6SC05355F-s001.pdf]

Electronic Supplementary Material (ESI) for Chemical Science.
This journal is © The Royal Society of Chemistry 2017

Electronic Supplementary Information

Template-directed synthesis of linear porphyrin oligomers: classical, Vernier and mutual Vernier

Nuntaporn Kamonsutthipaijit and Harry L. Anderson

Department of Chemistry, University of Oxford, Chemistry Research Laboratory, Oxford OX1 3TA, UK

Table of Contents

A. General methods **S2**

B. Synthetic procedures **S2**
B1. Synthesis of known compounds S2
B2. Synthesis of novel compounds S2
Fully deprotected Ni pyridyl porphyrin dimer **NiP2** S2
Phenylacetylene Zn porphyrin tetramer **PhC_2-ZnP4** S3
Phenylacetylene Ni porphyrin tetramer **PhC_2-NiP4** S4
Phenylacetylene Ni porphyrin hexamer **PhC_2-NiP6** S5
Fully THS protected Zn porphyrin heptamer **THS-ZnP7**, octamer **THS-ZnP8** and dodecamer **THS-ZnP12** S6
Fully deprotected Zn porphyrin dodecamer **ZnP12** S7
B3. Linear template-directed synthesis S8
B3.1 Classical templated synthesis of **NiP4** with **PhC_2-ZnP4** S8
B3.2. Classical templated synthesis of **ZnP4** with **PhC_2-NiP4** S9
B3.3. Classical templated synthesis of **NiP6** with **THS-ZnP6** S10
B3.4. Vernier templated synthesis of **ZnP12** with **PhC_2-NiP6** S11
B3.5. Mutual Vernier template-directed synthesis of **NiP12** and **ZnP12** with **NiP6** and **ZnP4** S12

C. Formation titrations of CPDIPS-ZnP1 with TMEDA and 2,2′-BiPy **S13**

D. Binding studies of molecular ladder complexes **S14**
D1. Determination of the binding constant of the 2-rung ladder complex S14
D2. Determination of the binding constant of the 4-rung ladder complex S17
D3. Determination of reference constant S19
D4. Calculation of effective molarities of 2- and 4-rung ladder complexes S20

E. Molecular structures of ladder complexes **S21**
E1. ^{1}H NMR Characterization S21
E1.1 ^{1}H NMR assignment of **CPDIPS-ZnP2•NiP2** S21
E1.2 ^{1}H NMR assignment of **CPDIPS-ZnP4•NiP4** S27
E1.3 Changes in chemical shifts for protons in **CPDIPS-ZnP4•NiP4** S37
E2. MALDI-ToF Mass analysis of **CPDIPS-ZnP4•NiP4** S38

F. Comparison of GPC yield ratios of Vernier templating reaction between 479 and 779 nm **S39**

G. MALDI-ToF mass analysis of mutual Vernier templating reaction **S39**

H. Analytical GPC calibration curve **S42**

I. Extinction coefficients of Zn and Ni oligomers per porphyrin unit **S43**

J. References **S43**

A. General methods

All chemicals were purchased from commercial suppliers and used without further purification unless otherwise stated. CuCl was freshly prepared.[1] Dry CH_2Cl_2 was obtained by passing through a column of activated alumina under nitrogen pressure. Diisopropylamine (DIPA) was dried over calcium hydride, distilled and stored under nitrogen over molecular sieves. "Petrol ether" (PE) always refers to 40/60 petrol ether. NMR data were recorded at 500 MHz using a Bruker AVII500 (with cryoprobe) or DRX500, or at 400 MHz using a Bruker AVII400 or AVIII400, or at 700 MHz using a Bruker AVIII700 (with cryoprobe) at 298 K. Chemical shifts are quoted as parts per million (ppm) relative to residual $CHCl_3$ (δ_H 7.27 ppm for ^1H NMR and at δ_C 77.2 ppm for ^{13}C NMR) and coupling constants (J) are reported in Hertz. MALDI-TOF-MS was measured with a Waters MALDI Micro MX spectrometer. *Trans*-2-[3-(4-*tert*-butylphenyl)-2-methyl-2-propenylidene]malononitrile (DTCB) was used as a matrix for all MALDI-TOF measurements. All UV-vis-NIR spectra were recorded at 25 °C on Perkin-Elmer Lambda 20 photospectrometer using quartz 1 cm cuvettes. UV-vis-NIR titrations were analyzed by calculating the difference in absorptions and plotted using Origin™ software or fitted with predicted models using SPECFIT™ software. Size exclusion chromatography (SEC) was carried out using Bio-Beads S-X1, 200–400 mesh (Bio Rad). Analytical and semi-preparative GPC were carried out on Shimadzu Recycling GPC system equipped with LC-20 AD pump, SPD-M20A UV detector and a set of JAIGEL 3H (20 × 600 mm) and JAIGEL 4H (20 × 600 mm) columns in toluene/1% pyridine as eluent with a flow rate of 3.5 mL/min or carried out on VWR Hitachi Lachrom Elite (pump L-2130, Autosampler L-2200, Diode Array Detector L-2450, Column oven L-2350) and a set of JAIGEL 3H-A (8 × 500 mm) and JAIGEL 4H-A (8 × 500 mm) columns in THF/1% pyridine as eluent with a flow rate of 1.0 mL/min.

B. Synthetic procedures

B1. Synthesis of known compounds

Fully protected Zn porphyrin monomer **CPDIPS-ZnP1**,[2] fully protected and fully deprotected Zn porphyrin dimer **THS-ZnP2**[3] and **ZnP2**,[3] fully protected and fully deprotected Zn porphyrin tetramer **THS-ZnP4**,[3] **CPDIPS-ZnP4**[2] and **ZnP4**,[2] Zn porphyrin pentamer **THS-ZnP5**[3], Zn porphyrin hexamer **THS-ZnP6**[3] and freebase-pyridyl porphyrin dimer **THS-H2P2py**[2] were synthesized by previously published procedures.

B2. Synthesis of Novel compounds

Fully deprotected Ni pyridyl porphyrin dimer **NiP2**

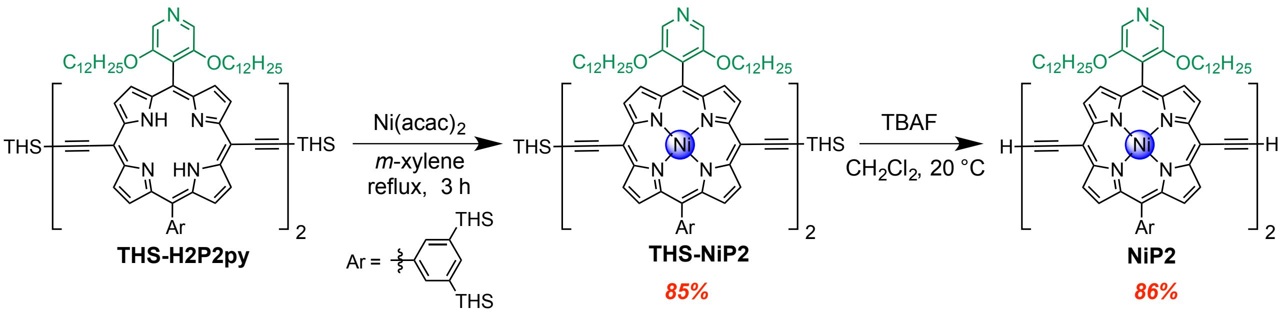

Fully protected Ni pyridyl porphyrin dimer, THS-NiP2: Freebase porphyrin dimer (50.0 mg, 0.014 mmol, 1.0 equiv.) was dissolved in *m*-xylene (10 mL). $Ni(acac)_2$ (108 mg, 0.420 mmol, 30.0 equiv.) was added. The mixture was heated to reflux under nitrogen for 3 h. After cooling the mixture was passed through a short silica plug eluting with chloroform/1% pyridine. The solvent was removed from the green filtrate and dried in vacuum, providing the desired product (44 mg, 85%) as a green solid.

THS-NiP2: ^{1}H NMR (400 MHz, $CDCl_3$): δ_H 9.63 (m, 4H, β-H), 9.46 (m, 4H, β-H), 8.80 (d, J = 4.0 Hz, 2H, β-H), 8.72 (d, J = 4.0 Hz, 2H, β-H), 8.68 (d, J = 4.0 Hz, 2H, β-H), 8.61 (d, J = 4.0 Hz, 2H, β-H), 8.45 (s, 4H, py-H), 8.10 (s, 4H, *o*-H), 7.95 (s, 2H, *p*-H), 3.98 (t, J = 4.0 Hz, 8H, OCH_2), 1.72–1.68 (m, 12H, alkyl-H),

1.56–1.30 (m, 150H, alkyl-H), 1.17–0.52 (m, 230H, alkyl-H). ***m/z* (MALDI-TOF)**: 3565.92 ($C_{226}H_{360}N_{10}Ni_2O_4Si_6$ requires 3562.56).

Fully deprotected Ni pyridyl porphyrin dimer, NiP2: Ni-dimer (32.7 mg, 0.009 mmol, 1.0 equiv.) was dissolved in chloroform (5 mL). TBAF-solution (1.0 M in THF, 5.0 µL, 0.005 mmol, 0.5 equiv.) was added and the mixture was stirred for 30 min at 20 °C. Methanol was layered and precipitation was completed in the fridge overnight. The green precipitate was filtered off, washed with methanol and dried in vacuum, affording the desired product (28.1 mg, 86% yield).

NiP2: ^{1}H NMR (400 MHz, $CDCl_3$): δ_H 9.63 (m, 4H, β-H), 9.46 (m, 4H, β-H), 8.81 (d, *J* = 4.0 Hz, 2H, β-H), 8.74 (d, *J* = 4.0 Hz, 2H, β-H), 8.68 (d, *J* = 4.0 Hz, 2H, β-H), 8.62 (d, *J* = 4.0 Hz, 2H, β-H), 8.43 (s, 4H, py-H), 8.08 (s, 4H, *o*-H), 7.94 (s, 2H, *p*-H), 4.07 (s, 2H, ethynyl-H), 3.96 (t, *J* = 4.0 Hz, 8H, OCH_2), 1.45–0.52 (m, 248H, alkyl-H). **^{13}C-deptQ-NMR** (125 MHz, $CDCl_3$): δ_C 155.3 (d, 4C, py-C), 145.9 (d, 2C, α-C), 145.7 (d, 2C, α-C), 145.3 (d, 2C, α-C), 145.1 (d, 2C, α-C), 143.4 (d, 2C, α-C), 143.2 (d, 2C, α-C), 143.0 (d, 2C, α-C), 142.8 (d, 2C, α-C), 139.9 (d, 2C, *ipso*-C), 139.6 (u, 4C, *o*-C), 138.5 (u, 2C, *p*-C), 135.6 (d, 4C, *m*-C), 133.6 (u, 2C, β-C), 133.3 (u, 2C, β-C), 132.6 (u, 2C, β-C), 132.4 (u, 2C, β-C), 132.3 (u, 2C, β-C), 132.1 (u, 2C, β-C), 131.6 (u, 2C, β-C), 131.4 (u, 2C, β-C), 129.1 (u, 4C, py-C), 126.2 (d, 2C, py-C), 122.7 (d, 2C, *meso*-C), 110.6 (d, 2C, *meso*-C), 99.3 (d, 2C, alkynyl-C), 98.7 (d, 2C, *meso*-C), 85.1 (d, 4C, *meso*-C and alkynyl-C), 84.7 (d, 2C, alkynyl-C), 82.5 (d, 2C, alkynyl-C), 69.9 (d, 4C, OCH_2), 33.7–22.7 (d, 108C, CH_2), 14.3 (u, 18C, CH_3), 14.2 (u, 4C, CH_3), 12.7 (d, 12C, CH_2-Si). ***m/z* (MALDI-TOF)**: 3001.23 ($C_{190}H_{284}N_{10}O_4Si_4Ni_2$ requires 3001.02). **λ_{max} (CH_2Cl_2)/ nm (log ε)**: 450 (5.28), 476 (5.24), 562 (4.34), 648 (4.76).

Phenylacetylene Zn porphyrin tetramer **PhA-ZnP4**

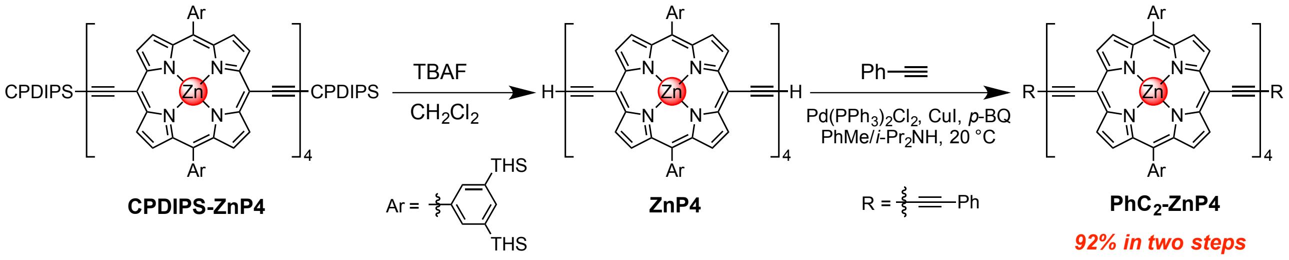

CPDIPS-ZnP4 (7.0 mg, 0.98 µmol, 1.0 equiv.) was dissolved in dry CH_2Cl_2 and then TBAF solution (1.0 M in THF; 3.0 µL, 2.44 µmol, 2.5 equiv.) was added. After stirring for 2 h, the reaction was complete. The crude mixture was passed through a small silica plug eluting with $CHCl_3$ to remove TBAF. The solvent was removed and dried under high vacuum. Then the crude product **ZnP4**, phenylacetylene (2.1 µL, 20 µmol, 20 equiv.), $Pd(PPh_3)_2Cl_2$ (1.0 mg, 2.0 µmol, 2.0 equiv.), copper(I) iodide (1.0 mg, 6.0 µmol, 6.0 equiv.) and 1,4-benzoquinone (1.0 mg, 10.0 µmol, 10.0 equiv.) were dissolved in dry toluene (1.00 mL), followed by dry DIPA (0.25 mL). The reaction mixture was stirred at 20 °C for 30 min and then further phenylacetylene (25.0 µL, 0.306 mmol, 312 equiv.) was added to ensure that all terminal acetylenes were coupled. The reaction mixture was stirred for another 1 h, then passed through a short alumina plug eluting with chloroform/1% pyridine, evaporated and redissolved in toluene to pass through a SEC-column to remove 1,4-benzoquinone and excess diphenylbutadiyne. The desired product was reprecipitated from layered chloroform/MeOH, affording **PhA-ZnP4** in 6.5 mg, 92% yield in two steps.

PhC_2-ZnP4: ^{1}H NMR (400 MHz, $CDCl_3$): δ_H 9.92 (m, 12H, β-H), 9.69 (d, *J* = 4.0 Hz, 4H, β-H), 8.98 (m, 12H, β-H), 8.91 (d, *J* = 4.0 Hz, 4H, β-H), 8.33 (s, 8H, *o*-H), 8.29 (s, 8H, *o*-H), 8.04 (s, 4H, *p*-H), 8.02 (s, 4H, *p*-H), 7.75 (m, 4H, Ph-H), 7.45 (m, 6H, Ph-H), 1.55–1.20 (m, 384H, CH_2), 0.99–0.82 (m, 240H, CH_2 and CH_3). **^{13}C-NMR** (125 MHz, $CDCl_3$): δ_C 153.2, 153.1, 153.0, 150.8, 150.7, 143.7, 140.9, 140.3, 139.4, 135.3, 133.5, 132.8, 131.2, 128.7, 125.1, 125.0, 122.6, 122.4, 100.8, 53.6, 33.7, 31.8, 24.2, 22.8, 14.3, 12.8. ***m/z* (MALDI-TOF)**: 7004.9 ($C_{448}H_{690}N_{16}Si_{16}Zn_4$ requires 7011.4).

Phenylacetylene Ni porphyrin tetramer **PhC$_2$-NiP4**

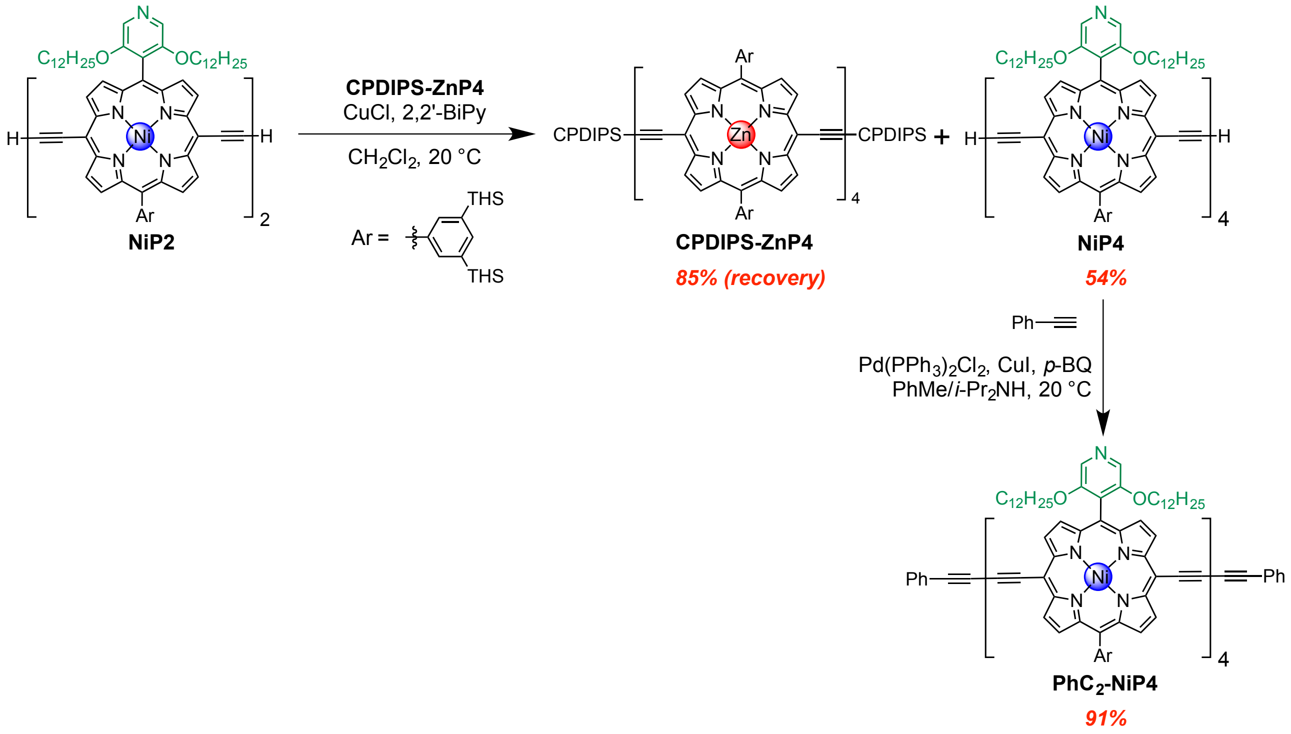

Firstly, **NiP4** was synthesized by linear templating with template **CPDIPS-ZnP4**. **NiP2** (22.3 mg, 7.43 μmol, 1.0 equiv.) was dissolved in dry CH_2Cl_2 (74 mL) using a 1-L-flask. Template **CPDIPS-ZnP4** (26.7 mg, 3.71 μmol, 0.5 equiv.) was added and the mixture was stirred for 20 min, monitoring by UV-vis-NIR spectroscopy. CuCl (103 mg, 1.04 mmol, 140 equiv.) was added, followed by **2,2′-BiPy** (162 mg, 1.04 mmol, 140 equiv.). The reaction mixture was stirred vigorously in a large flask with plenty of air. The reaction was monitored by UV-vis-NIR spectroscopy, and after 3 h it had gone to completion. To the reaction mixture, pyridine (7 mL) was added. Then crude product was subjected into a short silica column eluting with gradient 1%–10% pyridine/chloroform to separate the template and elute the Ni-porphyrin products. Then the crude Ni-product was passed through the small SEC-column. The solvent was removed and dried over vacuum. The crude product was dissolved in toluene/1% pyridine and filtered by microfilter. The product distribution was analyzed and separated by recycling GPC eluting with toluene/1% pyridine, affording the desired **NiP4** (12.3 mg, 54% yield). The template was recovered (22.6 mg, 85% recovered yield). Characterization data are shown in **Section 4.9.3.2**, the classical templated synthesis of **NiP4**.

Then **NiP4** (14.7 mg, 2.40 μmol, 1.0 equiv.), phenylacetylene (5.4 μL, 49.0 μmol, 20.0 equiv.), $Pd(PPh_3)_2Cl_2$ (1.0 mg, 2.00 μmol, 0.8 equiv.), copper(I) iodide (1.0 mg, 6.0 μmol, 2.5 equiv.) and 1,4-benzoquinone (1.0 mg, 10.0 μmol, 4.2 equiv.) were dissolved in dry toluene (4.0 mL), followed by dry DIPA (1 mL). The reaction mixture was stirred at 20 °C for 30 min and then further phenylacetylene (25 μL, 0.31 mmol, 312 equiv.) was added to ensure that all terminal acetylenes were coupled. The reaction mixture was stirred for another 1 h, then passed through a short alumina plug eluting with chloroform/1% pyridine, evaporated and redissolved in toluene to pass through a SEC-column to remove 1,4-benzoquinone and excess diphenylbutadiyne. The desired product was reprecipitated by bilayers of chloroform/MeOH, affording **PhC$_2$-NiP4** (13.5 mg, 91% yield).

PhC$_2$-NiP4: ^{1}**H NMR** (400 MHz, $CDCl_3$): δ_H = 9.64 (m, 12H, β-H), 9.47 (m, 4H, β-H), 8.82 (m, 6H, β-H), 8.76 (d, J = 8.0 Hz, 2H, β-H), 8.69 (m, 6H, β-H), 8.64 (d, J = 4.0 Hz, 2H, β-H), 8.48 (s, 4H, py-H), 8.46 (s, 4H, py-H), 8.14 (s, 4H, *o*-H), 8.11 (s, 4H, *o*-H), 7.98 (s, 2H, *p*-H), 7.97 (s, 2H, *p*-H), 7.70 (m, 4H, Ph-H), 7.45 (m, 6H, Ph-H), 3.98 (m, 16H, OCH_2), 1.47–0.56 (m, 496H, alkyl-H). ^{13}**C NMR** (125 MHz, $CDCl_3$): δ_C 155.5, 146.0, 145.8, 145.8, 143.2, 143.0, 140.0, 139.7, 138.4, 135.7, 133.7, 132.8, 132.2, 131.5, 129.5, 129.1, 128.7, 126.1, 125.2, 123.2, 123.1, 122.1, 111.2, 111.0, 99.2, 99.1, 99.1, 85.3, 84.3, 82.8, 82.7, 82.3, 81.8, 70.0, 69.9, 33.7, 31.9, 31.9, 31.8, 29.5, 29.5, 29.4, 29.3, 29.3, 29.2, 29.1, 28.8, 25.4, 24.2, 22.8, 22.7, 22.7, 14.3, 14.2, 14.1, 12.8. ***m/z* (MALDI-TOF)**: 6197.43 ($C_{396}H_{574}N_{20}Ni_4O_8Si_8$ requires 6202.08).

Phenylacetylene Ni porphyrin hexamer **PhC_2-NiP6**

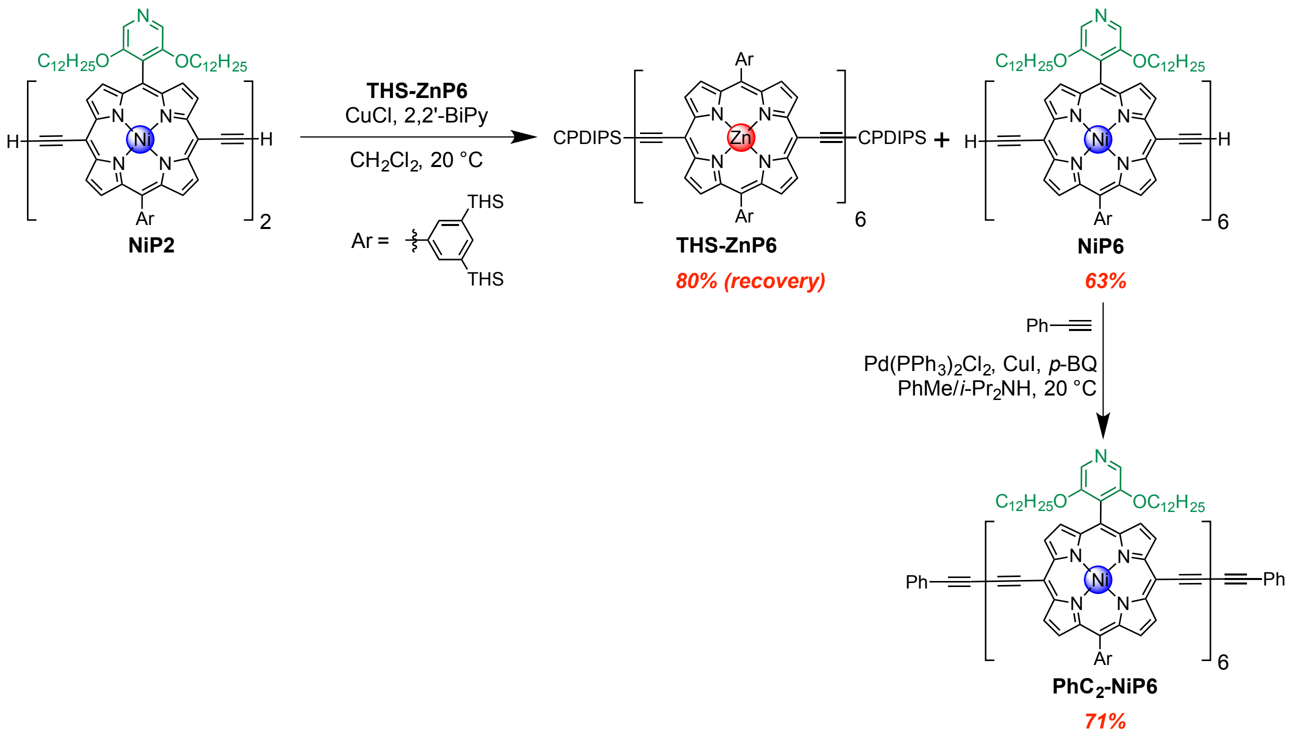

Firstly, **NiP6** was synthesized by linear templating with template **THS-ZnP6**. **NiP2** (20 mg, 6.6 μmol, 1.0 equiv.) and template **THS-ZnP6** (24 mg, 2.2 μmol, 0.33 equiv.) were dissolved in dry CH_2Cl_2 (74 mL) using a 1-L-flask. The mixture was stirred for 20 min, monitoring by UV-vis-NIR spectroscopy. CuCl (92 mg, 0.93 mmol, 140 equiv.) was added, followed by **2,2′-BiPy** (145 mg, 0.93 mmol, 140 equiv.). The reaction mixture was stirred vigorously in the large flask with plenty of air. The reaction was followed by UV-vis-NIR spectroscopy, and after 3 h it had gone to completion. To the reaction mixture, pyridine (7 mL) was added. Then crude product was subjected into a short silica column eluting with gradient chloroform/1%–10% pyridine to remove all the coupling reagents, separate the template and elute the Ni-porphyrin products. The solvent was removed from the crude Ni product and dried over vacuum. The crude product was dissolved in toluene/1% pyridine and filtered by microfilter. The product distribution was separated by recycling GPC eluting with toluene/1% pyridine, affording the desired **NiP6** (12.6 mg, 63% yield). The template was recovered (19 mg, 80% recovered yield). Characterization data are shown in **Section 4.9.3.3**, the classical templated synthesis of **NiP6**.

Then **NiP6** (5.0 mg, 0.56 μmol, 1.0 equiv.), phenylacetylene (25.0 μL, 228 μmol, 407 equiv.), $Pd(PPh_3)_2Cl_2$ (1.0 mg, 2.0 μmol, 3.6 equiv.), copper(I) iodide (1.0 mg, 6.0 μmol, 11 equiv.) and 1,4-benzoquinone (1.0 mg, 10 μmol, 18 equiv.) were dissolved in dry CH_2Cl_2 (1.60 mL) and pyridine (0.05 mL), followed by dry DIPA (0.40 mL). The reaction was stirred at 20 °C for 30 min and then phenylacetylene (25.0 μL) was added to ensure that all terminal acetylenes were coupled. The reaction mixture was stirred further for another 4 h. The reaction mixture was passed through a short silica plug eluting with chloroform/1%–10% pyridine and then removed all solvent and redissolved in toluene to pass through a SEC-column to remove 1,4-benzoquinone and excess diphenylbutadiyne. The desired product was reprecipitated by bilayers of hexane/chloroform/MeOH and then hexane/MeOH, affording **PhC_2-NiP6** (3.5 mg, 71% yield).

PhC_2-NiP6: ^{1}H NMR (500 MHz, $CDCl_3$): δ_H 9.64 (m, 20H, β-H), 9.47 (m, 4H, β-H), 8.82 (m, 10H, β-H), 8.75 (d, J = 5.0 Hz, 2H, β-H), 8.70 (m, 10H, β-H), 8.63 (d, J = 5.0 Hz, 2H, β-H), 8.47 (broad s, 12H, py-H), 8.14 (s, 8H, *o*-H), 8.11 (s, 4H, *o*-H), 7.98 (s, 4H, *p*-H), 7.96 (s, 2H, *p*-H), 7.70 (m, 4H, Ph-H), 7.44 (m, 6H, Ph-H), 4.02 (m, 24H, OCH_2), 1.47–0.54 (m, 744H, alkyl-H). **^{13}C NMR** (125 MHz, $CDCl_3$): δ_C 145.9, 145.7, 143.2, 143.0, 139.9, 139.7, 138.4, 135.7, 133.7, 132.8, 132.2, 131.5, 129.1, 128.9,128.7, 125.1, 123.2, 99.2, 69.9, 69.9, 38.9, 33.7, 32.3, 32.1, 31.9, 31.8, 31.8, 29.8, 29.5, 29.4, 29.3, 29.3, 29.2, 29.1, 28.8, 26.5, 25.4, 24.2, 23.9, 23.6, 23.1, 22.8, 22.7, 22.6, 14.3, 14.2, 14.1, 12.7. ***m/z* (MALDI-TOF)**: 9202.7 ($C_{586}H_{856}N_{30}Ni_6O_{12}Si_{12}$ requires 9202.08)

Fully THS protected Zn porphyrin heptamer **THS-ZnP7**, *octamer* **THS-ZnP8** *and dodecamer* **THS-ZnP12**

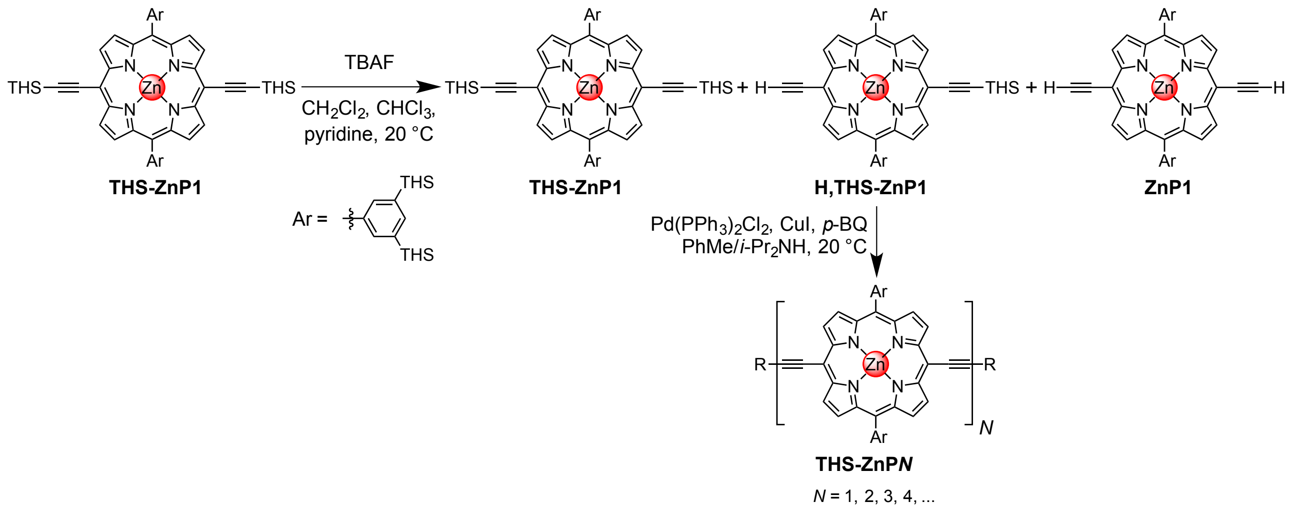

THS-ZnP1 (790 mg, 0.35 mmol, 1.0 equiv.) was dissolved in CH_2Cl_2 (52 mL), chloroform (26 mL) and pyridine (7.0 mL). The solution was cooled in a water-ice bath and TBAF (520 μL, 1.0 M in THF) was added. The mixture was stirred and frequently monitored by TLC (petrol/toluene 98:2). As soon as the preferred ratio of deprotection was reached the reaction was quenched with acetic acid (few drops) and the mixture of **THS-ZnP1**, **H,THS-ZnP1** and **ZnP1** was filtered through a short column of silica gel (CH_2Cl_2/ 1% pyridine).

The crude mixture was used without further purification for the statistical coupling reaction. The crude mixture was dissolved in dry toluene (40 mL). $PdCl_2(PPh_3)_2$ (122 mg, 174 μmol, 0.5 equivalent), copper(I) iodide (684 mg, 4.9 mmol, 14 equivalent), 1,4-benzoquinone (677 mg, 6.3 mmol, 18 equivalent) and dry *i*Pr_2NH (10 mL) were finally added. The reaction was monitored by TLC. After one and a half hour, all **H,THS-ZnP1** and **ZnP1** had gone. The volume was reduced and the mixture was filtered through a short column of silica gel (Petrol ether/CH_2Cl_2/ 1 % pyridine). The mixture was passed through a SEC column (toluene/ 1% pyridine) to remove **THS-ZnP1** and 1,4-benzoquinone. The crude porphyrin oligomers were further purified by GPC eluting with toluene/1% pyridine. The forth cycle of GPC traces is shown below:

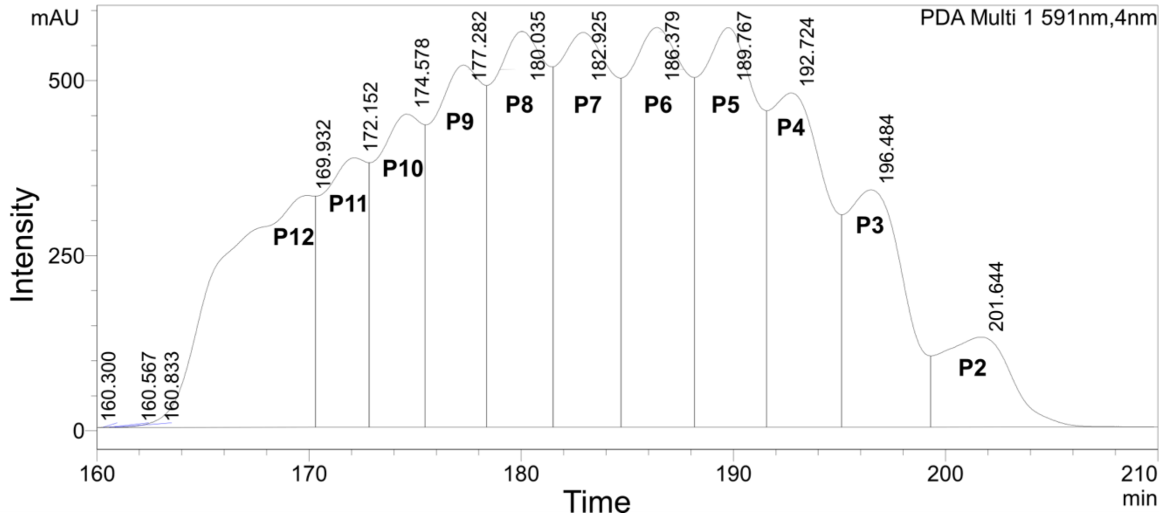

THS-ZnP2 (2%), **THS-ZnP4** (5%), **THS-ZnP5** (6%), **THS-ZnP6** (6%), **THS-ZnP7** (6%), **THS-ZnP8** (5%) and **THS-ZnP12** (3%) were isolated by recycling GPC to be used as references for making analytical GPC calibration curve for the THF/1% pyridine GPC column as see detail in **Section 4.9.4**.

THS-ZnP2, **THS-ZnP4**, **THS-ZnP5** and **THS-ZnP6** were characterized as literature.[3]

THS-ZnP7: ^{1}**H NMR** (400 MHz, $CDCl_3$): δ_H 9.90 (m, 24H, β-H), 9.61 (d, J = 4.0 Hz, 4H, β-H), 8.96 (m, 24H, β-H), 8.86 (d, J = 4.0 Hz, 4H, β-H), 8.32 (s, 20H, *o*-H), 8.26 (s, 8H, *o*-H), 8.02 (s, 10H, *p*-H), 8.00

(s, 4H, *p*-H), 1.55–0.89 (m, 1170H, CH_2 and CH_3). ***m/z*** **(MALDI-TOF)**: 12637.4 ($C_{792}H_{1268}N_{28}Si_{30}Zn_7$ requires 12460.8).

THS-ZnP8: ^{1}H NMR (400 MHz, $CDCl_3$): δ_H 9.91 (m, 28H, β-H), 9.68 (d, *J* = 4.0 Hz, 4H, β-H), 8.96 (m, 28H, β-H), 8.86 (d, *J* = 4.0 Hz, 4H, β-H), 8.33 (s, 24H, *o*-H), 8.27 (s, 8H, *o*-H), 8.03 (s, 12H, *p*-H), 8.00 (s, 4H, *p*-H), 1.56–0.89 (m, 1326H, CH_2 and CH_3). ***m/z*** **(MALDI-TOF)**: 13993.6 ($C_{900}H_{1438}N_{32}Si_{34}Zn_8$ requires 14160.0).

THS-ZnP12: ^{1}H NMR (400 MHz, $CDCl_3$): δ_H 9.91 (m, 44H, β-H), 9.67 (d, *J* = 4.0 Hz, 4H, β-H), 8.95 (m, 44H, β-H), 8.86 (d, *J* = 4.0 Hz, 4H, β-H), 8.33 (s, 40H, *o*-H), 8.27 (s, 8H, *o*-H), 8.03 (s, 20H, *p*-H), 8.00 (s, 4H, *p*-H), 1.56–0.89 (m, 1950H, CH_2 and CH_3). ***m/z*** **(MALDI-TOF)**: 21441.3 ($C_{1332}H_{2118}N_{48}Si_{50}Zn_{12}$ requires 20956.7).

Fully deprotected Zn porphyrin dodecamer **ZnP12**

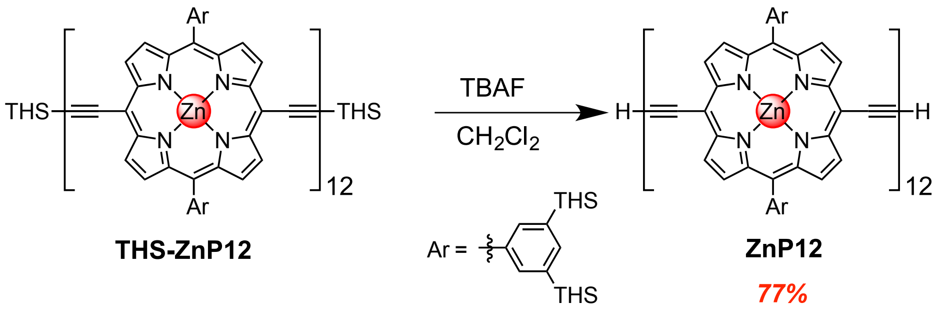

THS-ZnP12 (5.0 mg, 0.24 μmol, 1.0 equiv.) was dissolved in CH_2Cl_2 (1.0 mL), chloroform (1.0 mL) and pyridine (20 μL) and then added TBAF solution (1.0 M in THF; 2.4 μL, 2.4 μmol, 2.5 equiv.). After stirring for 10 minutes, the reaction was completed. The crude mixture was passed through a small silica plug eluting with $CHCl_3$ to remove TBAF. The solvent was removed and dried under high vacuum, providing **ZnP12** (3.8 mg, 77% yield).

ZnP12: ^{1}H NMR (400 MHz, $CDCl_3$): δ_H 9.99 (m, 44H, β-H), 9.76 (d, *J* = 4.0 Hz, 4H, β-H), 9.06 (m, 44H, β-H), 8.97 (d, *J* = 4.0 Hz, 4H, β-H), 8.38 (s, 40H, *o*-H), 8.32 (s, 8H, *o*-H), 8.07 (s, 20H, *p*-H), 8.04 (s, 4H, *p*-H), 4.22 (s, 2H, ethynyl-H), 1.56–0.89 (m, 1872H, CH_2 and CH_3).

m/z **(MALDI-TOF)**: 20847.2 ($C_{1296}H_{2042}N_{48}Si_{48}Zn_{12}$ requires 20392.17).

B3. Linear template-directed synthesis

*B3.1 Classical templated synthesis of **NiP4** with **PhC$_2$-ZnP4***

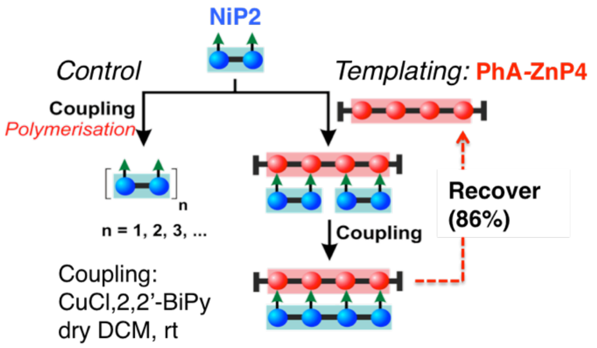

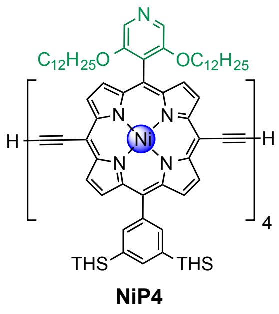

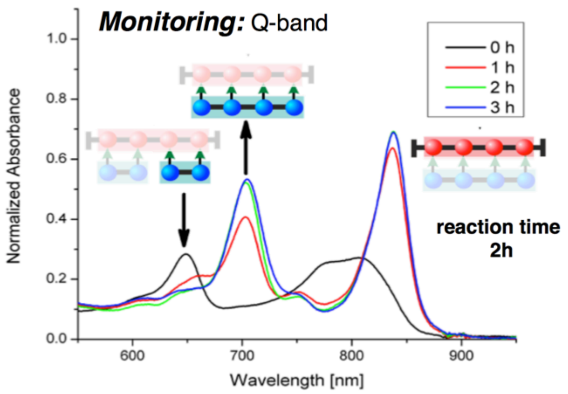

NiP2 (6.20 mg, 2.24 µmol, 1.0 equiv.) was dissolved in dry CH_2Cl_2 (20.7 mL) and 10 mL of this solution was aliquoted into a 50-mL-flask. Template **PhC$_2$-ZnP4** (3.40 mg, 0.53 µmol, 0.5 equiv.) was added and the mixture was stirred for 20 min, monitoring by UV-vis-NIR spectroscopy. CuCl (13.9 mg, 0.14 mmol, 140 equiv.) was added, followed by **2,2′-BiPy** (21.9 mg, 0.14 mmol, 140 equiv.). A second reaction was set up, similar with respect to the above, but in the absence of **PhC$_2$-ZnP4**. The reactions were followed carefully by UV-vis-NIR spectroscopy, and after 3 h had gone to completion. To each of the reaction mixtures, 0.1 mL of pyridine was added. Then crude product was subjected into a short silica column eluting with gradient 1%-10% pyridine/chloroform to separate the Zn template and elute the Ni-porphyrin products. The Zn template can be recovered in 2.9 mg, 86% yield. Then the crude Ni-product was passed through the small SEC-column eluting with toluene/1% pyridine. The solvent was removed and dried over vacuum. The crude product was dissolved in toluene/1% pyridine and filtered by microfilter. The product distribution was analyzed and separated by recycling GPC eluting with toluene/1% pyridine. The desired product **NiP4** was obtained (1.7 mg, 57% isolated yield) and the unreacted substrate was reisolated (0.3 mg, 10% yield).

The reaction was set up again but changed the template **PhC$_2$-ZnP4** to **CPDIPS-ZnP4**, providing the desired product **NiP4** in 13.7 mg, 30% yield from 39 mg of **NiP2**, in 1.26 mg, 42% yield from 3.0 mg of **NiP2** and in 11.9 mg, 54% yield from 22 mg of **NiP2**.

Monitoring reaction by UV-vis-NIR spectroscopy: The absorption spectra of porphyrins consist of two bands called a B-band (400–550 nm) and a Q-band (650–950 nm). The absorption spectra of Ni and Zn porphyrins are distinctive for equal oligomer length because the Q-band of Ni porphyrins occurs at shorter wavelengths, making it easy to observe changes in the course of the reaction. As shown in absorption spectra, initially, before the start of the coupling reaction, the absorption spectra show two Q-bands at the short (648 nm) and long (808 nm) wavelengths corresponding to the bound Ni dimer and the Zn template on the ladder complex, respectively. Over the course of the reaction the Q-band of **NiP2** starts to decrease and a new Q-band appears at the red-shifted wavelength of 704 nm, which indicates the formation of longer Ni porphyrin oligomers, including the desired product **NiP4.** At the same time, the Q-band of the Zn template is red-shifted and becomes sharper at 838 nm, indicating that the Zn template is held in a more rigid and conjugated conformation.[4,5] After 2 h, there is no further change in the absorption spectra.

NiP4: ^{1}**H NMR** (400 MHz, $CDCl_3$): δ_H 9.65 (m, 12H, β-H), 9.48 (dd, J = 4.8, 5.2 Hz, 4H, β-H), 8.83 (m, 6H, β-H), 8.76 (d, J = 4.8 Hz, 2H, β-H), 8.70 (m, 6H, β-H), 8.64 (d, J = 5.2 Hz, 2H, β-H), 8.46 (d, J = 7.6 Hz, 8H, py-H), 8.13 (d, 8H, *o*-H), 7.97 (d, 4H, *p*-H), 4.07 (s, 1H, ethynyl-H), 4.01 (m, 16H, OCH_2), 1.47–0.55 (m, 496H, alkyl-H). ^{13}**C NMR** (125 MHz, $CDCl_3$): δ_C 175.5, 155.3, 145.9, 143.0, 139.9, 135.7, 132.4, 130.2, 129.9, 129.1, 70.0, 69.9, 36.0, 33.7, 32.1, 31.9, 31.9, 31.8, 29.9, 29.8, 29.7, 29.5, 29.4, 29.4, 29.4, 29.3, 29.3, 29.2, 29.1, 29.1, 28.7, 27.4, 27.3, 25.7, 25.4, 25.4, 24.2, 22.8, 22.7, 22.7, 14.3, 14.2, 14.1, 12.7. ***m/z* (MALDI-TOF)**: 5997.73 ($C_{380}H_{566}N_{20}Ni_4O_8Si_8$ requires 6001.01). **λ_{max} (CH_2Cl_2)/ nm (log *ε*)**: 445 (5.56), 485 (5.61), 561 (4.69), 683 (5.30).

NiP2: as described in **Section 4.9.2.2**

B3.2. Classical templated synthesis of **ZnP4** *with* **PhC_2-NiP4**

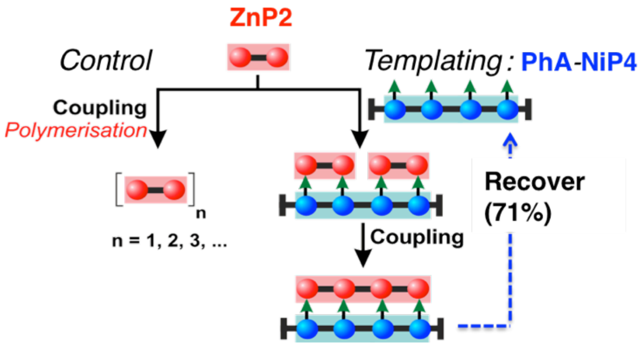

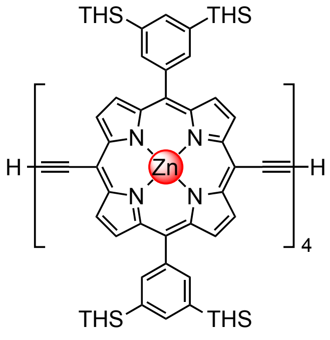

ZnP4

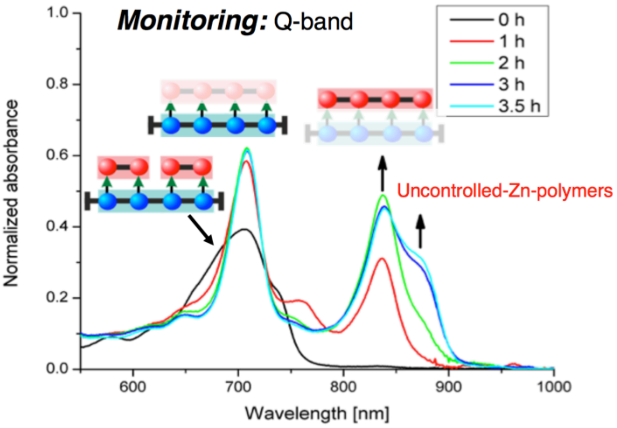

ZnP2 (7.0 mg, 2.05 μmol, 1.0 equiv.) was dissolved in dry CH_2Cl_2 (20.5 mL) and 10 mL of this solution was aliquoted into a 50-mL-flask. Template **PhC_2-NiP4** (3.10 mg, 0.50 μmol, 0.5 equiv.) was added and the mixture was stirred for 20 min, monitoring by UV-vis-NIR spectroscopy. CuCl (13.9 mg, 0.14 mmol, 140 equiv.) was added, followed by **2,2′-BiPy** (21.9 mg, 0.14 mmol, 140 equiv.). A second reaction was set up, similar with respect to the above, but in the absence of **PhC_2-NiP4**. The reactions were followed carefully by UV-vis-NIR spectroscopy, and after 3.5 h had gone to completion. To each of the reaction mixtures, 0.1 mL of pyridine was added. Then crude product was subjected into a short silica column eluting with gradient 1%–10% pyridine/chloroform to separate the Zn-porphyrin products and elute the Ni template. The Ni template was recovered in 2.2 mg, 71% yield. Then the crude Zn-products were passed through the small SEC-column eluting with toluene/1% pyridine. The solvent was removed and dried over vacuum. The crude product was dissolved in toluene/1% pyridine and filtered by microfilter. The product distribution was analyzed and separated by recycling GPC eluting with toluene/1% pyridine. The desired product **ZnP4** was isolated (0.78 mg, 23% yield).

The reaction was set up again but reduced the reaction time to 1.5 h, providing the desired product (1.02 mg, 30% yield).

Monitoring reaction by UV-vis-NIR spectroscopy: UV-vis-NIR spectroscopy before addition of the coupling reagents showed overlapping absorptions between the substrate **ZnP2** and the template **PhA-NiP4** because the red-shift of the absorption of longer Ni-containing oligomers causes the Q-band to come in the same region as shorter Zn oligomers. After 1 h, the planarization of the template provides a slightly red-shifted and sharper Q-band at 709 nm and a new Q-band corresponding to the longer oligomers (most likely the desired product **ZnP4**) appears at longer wavelengths (838 nm). Then at 2 h, another species appears with a far-red shifted absorption at 875 nm, possibly corresponding to longer Zn porphyrin polymers. Since the peak at 875 nm increases with longer reaction times, the reaction was stopped after 3.5 h at the first attempt.

ZnP4: ^{1}**H NMR** (400 MHz, $CDCl_3$): δ_H 9.65 (m, 12H, β-H), 9.48 (dd, J = 4.8, 5.2 Hz, 4H, β-H), 8.83 (m, 6H, β-H), 8.76 (d, J = 4.8 Hz, 2H, β-H), 8.70 (m, 6H, β-H), 8.64 (d, J = 5.2 Hz, 2H, β-H), 8.46 (d, J = 7.6 Hz, 8H, py-H), 8.13 (d, 8H, *o*-H), 7.97 (d, 4H, *p*-H), 4.07 (s, 1H, ethynyl-H), 4.01 (m, 16H, OCH_2), 1.47–0.55 (m, 496H, alkyl-H). ***m/z* (MALDI-TOF)**: 5997.73 ($C_{380}H_{566}N_{20}Ni_4O_8Si_8$ requires 6001.01).

These data match previous literature reports.[2]

*B3.3. Classical templated synthesis of **NiP6** with **THS-ZnP6***

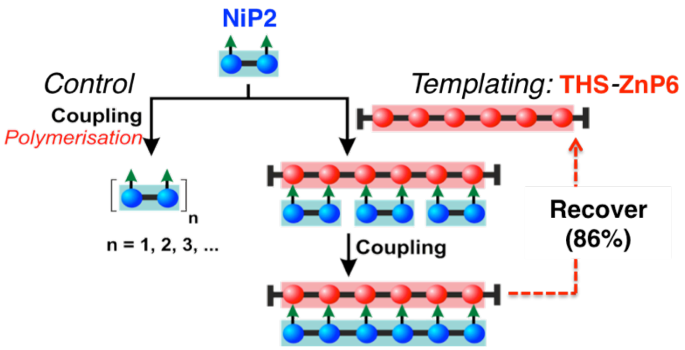

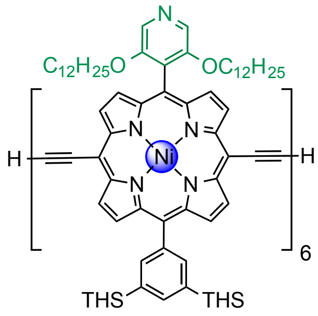

NiP6

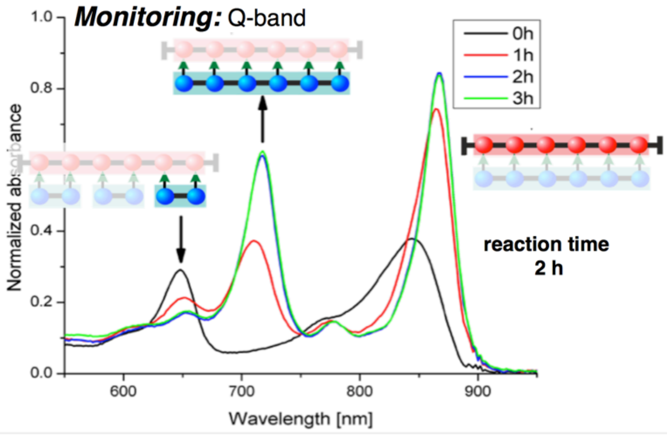

NiP2 (9.0 mg, 3.0 µmol, 1.0 equiv.) was dissolved in dry CH_2Cl_2 (33.3 mL) into a 1000-mL-flask. Template **THS-ZnP6** (10.7 mg, 1.0 µmol, 0.33 equiv.) was added and the mixture was stirred for 20 min, monitoring by UV-vis-NIR spectroscopy. CuCl (41.5 mg, 0.42 mmol, 140 equiv.) was added, followed by **2,2′-BiPy** (65 mg, 0.42 mmol, 140 equiv.). The non-templated reaction was prepared similarly as the templated reaction but in the absence of template **THS-ZnP6**. The reaction was followed carefully by UV-vis-NIR spectroscopy, and after 3 h had gone to completion. Pyridine (0.3 mL) was added. Then crude product was subjected into a short silica column eluting with 20% pyridine/chloroform to remove the coupling reagent. Then the crude mixture was passed through the small SEC-column eluting with toluene/1% pyridine. The solvent was removed and dried over vacuum. The crude product was dissolved in toluene/1% pyridine and filtered by microfilter. The product distribution was analyzed and separated by recycling GPC eluting with toluene/1% pyridine. The template **THS-ZnP6** was separated and recovered in 9.2 mg, 86%yield. The desired product **NiP6** was separated (5.1 mg, 56% yield).

Monitoring reaction by UV-vis-NIR spectroscopy: before the start of the coupling reaction, the absorption spectra show two Q-bands at 648 nm and 844 nm corresponding to the bound Ni dimer and the Zn hexamer template on the ladder complex, respectively. During the reaction the Q-band of **NiP2** starts to decrease and a new Q-band appears at the red-shifted wavelength of 718 nm, which indicates the formation of longer Ni porphyrin oligomers, including the desired product **NiP6**. At the same time, the Q-band of the Zn template is red-shifted and becomes sharper at 867 nm. After 2 h, there is no further change in the absorption spectra.

NiP6: ^{1}**H NMR** (400 MHz, $CDCl_3$): δ_H 9.64 (m, 20H, β-H), 9.47 (m, 4H, β-H), 8.82 (d, J = 4.0 Hz, 10H, β-H), 8.75 (d, J = 4.0 Hz, 2H, β-H), 8.70 (d, J = 8.0 Hz, 10H, β-H), 8.63 (d, J = 4.0 Hz, 2H, β-H), 8.47 (s, 8H, py-H), 8.45 (s, 4H, py-H), 8.13 (s, 8H, *o*-H), 8.10 (s, 4H, *o*-H), 7.97 (s, 4H, *p*-H), 7.95 (s, 2H, *p*-H), 4.05 (s, 2H, ethynyl-H), 3.96 (m, 24H, OCH_2), 1.47–0.55 (m, 744H, alkyl-H). ^{13}**C NMR** (125 MHz, $CDCl_3$): δ_C 175.5, 155.3, 145.9, 145.7, 143.2, 143.0, 139.9, 139.7, 138.4, 135.7, 133.7, 132.8, 132.2, 131.5, 129.1, 128.9, 128.7, 125.1, 123.2, 99.2, 69.9, 69.9, 38.9, 33.7, 32.3, 32.1, 31.9, 31.8, 31.8, 29.8, 29.5, 29.4, 29.3, 29.3, 29.2, 29.1, 28.8, 26.5, 25.4, 24.2, 23.9, 23.6, 23.1, 22.8, 22.7, 14.3, 14.2, 14.1, 12.7. ***m/z*** **(MALDI-TOF)**: 8996.17 ($C_{570}H_{848}N_{30}Ni_6O_{12}Si_{12}$ requires 9002.01). λ_{max} **(CH_2Cl_2)/ nm (log ε)**: 448 (5.67), 485 (5.78), 562 (4.83), 708 (5.5).

By-product **NiP12** from **NiP6**

After pure **NiP6** was obtained from the templating reaction, it was kept in the freezer at –20 °C for two weeks. The Glaser coupling reaction of **NiP6** gradually occurred, providing the by-products. The crude mixture was injected into GPC (THF/1% pyridine). The by-product was isolated and identified by ^{1}H NMR and MALDI-ToF mass analysis, confirming **NiP12**. This compound can be used as a reference for the mutual Vernier templating reaction.

NiP12: ^{1}**H NMR** (500 MHz, $CDCl_3$): δ_H 9.63 (m, 44H, β-H), 9.46 (m, 4H, β-H), 8.81 (m, 20H, β-H), 8.74 (d, J = 5.0 Hz, 2H, β-H), 8.68 (m, 20H, β-H), 8.62 (d, J = 5.0 Hz, 2H, β-H), 8.46 (s, 20H, py-H), 8.43 (s, 4H, py-H), 8.12 (s, 20H, *o*-H), 8.09 (s, 4H, *o*-H), 7.96 (s, 10H, *p*-H), 7.94 (s, 2H, *p*-H), 4.04 (s, 2H, ethynyl-H), 3.96 (m, 48H, OCH_2), 1.47–0.62 (m, 1488H, alkyl-H).

m/z **(MALDI-TOF)**: 18106.9 ($C_{1140}H_{1696}N_{60}Ni_{12}O_{24}Si_{24}$ requires 18002.9).

B3.4. Vernier templated synthesis of ***ZnP12*** *with* ***PhC_2-NiP6***

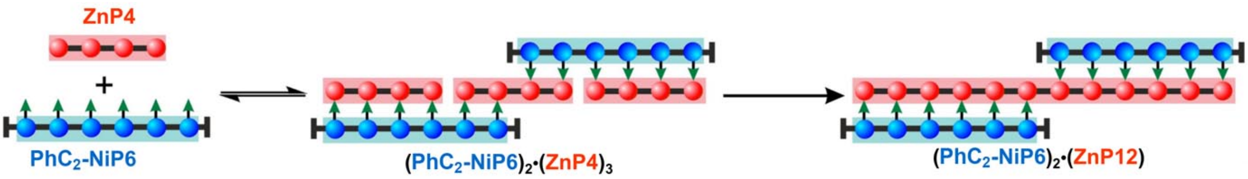

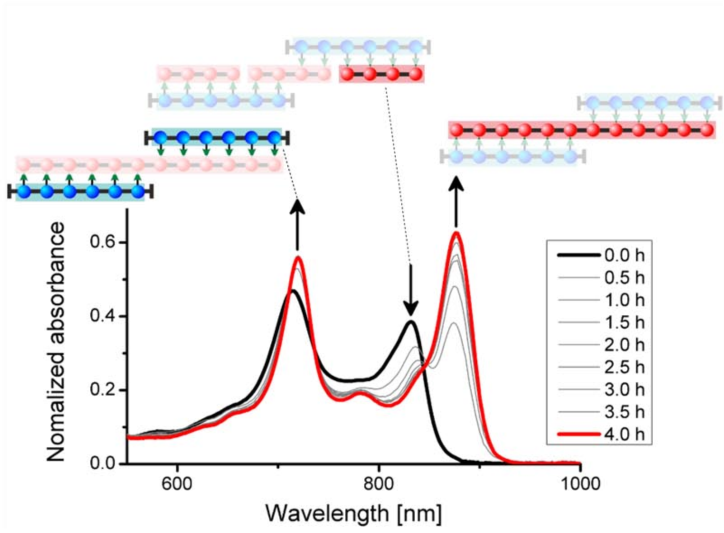

ZnP4 (3.5 mg, 0.51 μmol, 1.0 equiv.) was dissolved in dry CH_2Cl_2 (3.0 mL) into a 25-mL-flask. Template **PhC_2-NiP6** (3.2 mg, 0.30 μmol, 0.60 equiv.) was dissolved in dry CH_2Cl_2 (3.0 mL) and then added

into the Zn porphyrin solution. The mixture was stirred for 20 min, monitoring by UV-vis-NIR spectroscopy. CuCl (7.1 mg, 0.07 mmol, 140 equiv.) was added, followed by **2,2′-BiPy** (11 mg, 0.07 mmol, 140 equiv.). The reactions were followed carefully by UV-vis-NIR spectroscopy, and after 4 h, there was insignificant change in absorption spectrum. To the reaction mixture, 0.6 mL of pyridine was added. Then crude product was subjected into a short silica column eluting with gradient chloroform/1%–10% pyridine to separate the Zn-porphyrin products and elute the Ni template. Then the crude Zn-products were passed through the small SEC-column eluting with toluene/1% pyridine. The solvent was removed and dried over vacuum. The crude product was dissolved in toluene/1% pyridine and filtered by microfilter. The product distribution was analyzed by recycling GPC eluting with toluene/1% pyridine based on the GPC calibration curve as published.[6] The desired product **ZnP12** was analyzed by fitting peak area in 21% yield.

Monitoring reaction by UV-vis-NIR spectroscopy: Before addition of the coupling reagents, the absorption spectra show two Q-bands at 714 nm and 831 nm corresponding to the bound Ni hexamer template and the Zn tetramer on the complex, respectively. During the reaction the Q-band of **ZnP4** starts to decrease and a new Q-band appears at the red-shifted wavelength of 877 nm, which indicates the formation of longer Zn porphyrin oligomers, including the desired product **ZnP12**. At the same time, the Q-band of the Ni template is slightly red-shifted and becomes sharper at 721 nm. After 3–4 h, there was insignificant change in the absorption spectrum. During GPC analysis (Fig. 10) analytical yields were estimated from the deconvoluted areas of the GPC peaks from the absorption at 591 nm, since only Zn porphyrin oligomers have the same extinction coefficient per porphyrin unit at this wavelength.

B3.5. Mutual Vernier template-directed synthesis of ***NiP12*** *and* ***ZnP12*** *with* ***NiP6*** *and* ***ZnP4***

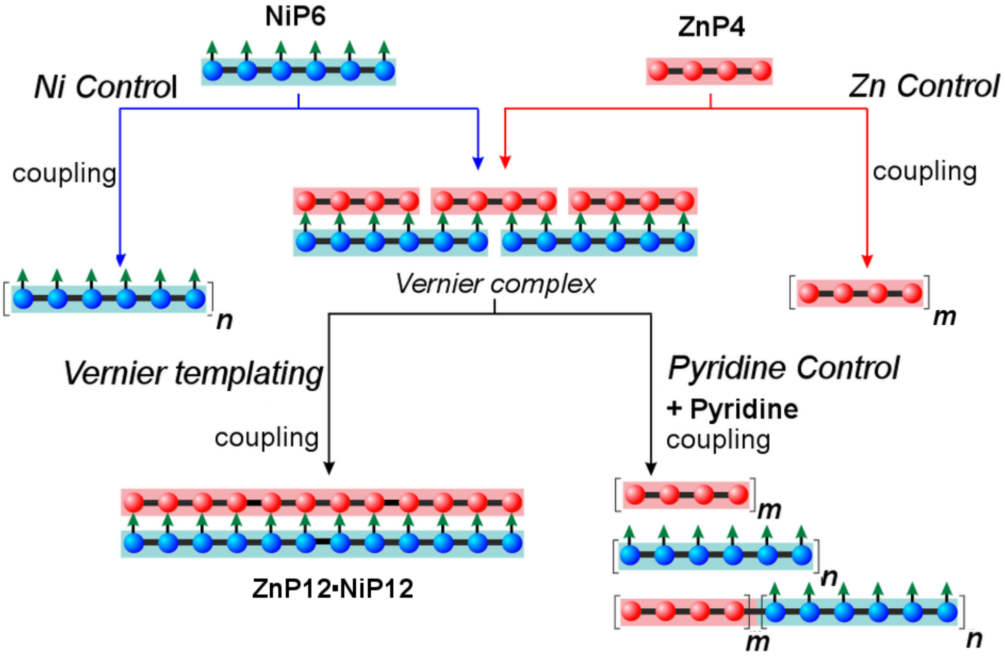

NiP6 (5.0 mg, 0.56 µmol, 1.0 equiv.) was dissolved in dry CH_2Cl_2 (3.3 mL) (Ni-stock solution). **ZnP4** (5.38 mg, 0.79 µmol, 1.5 equiv.) was dissolved in dry CH_2Cl_2 (9.0 mL) (Zn-stock solution). 1.0 mL of each stock solution was aliquoted into a 100-mL-flask. The mixture was further diluted with 1.0 mL of dry CH_2Cl_2. The mixture was stirred for 20 min. CuCl (2.36 mg, 24.0 µmol, 140 equiv.) was added, followed by **2,2′-BiPy** (3.72 mg, 24.0 µmol, 140 equiv.) 3 times at 0, 3 and 6 h. The Ni-control reaction was set up similarly with respect to the above but added dry CH_2Cl_2 (1.0 mL) instead of the Zn-stock solution. The Zn-control reaction was set up similarly with the previous reaction but added dry CH_2Cl_2 (1.0 mL) instead of the Ni-stock solution. The pyridine-control reaction was set up similarly with the first reaction but also added pyridine (0.3 mL). The reaction mixtures were stirred vigorously with the dry tube at 20 °C. 20 µL of the linear Vernier templating reaction was aliquoted at 1, 3, 4, 6, 7 and 8 h and passed through a short silica plug eluting with CH_2Cl_2/10% pyridine. Then all solvents were removed before injecting to GPC (THF/1% pyridine) to monitor the starting material consuming by time. All reactions were stopped after 8 h and then passed through a short silica plug eluting with CH_2Cl_2/10% pyridine. The crude product mixture was analyzed by GPC eluting with THF/1% pyridine and MALDI-ToF MS.

C. Formation titrations of CPDIPS-ZnP1 with TMEDA and 2,2′-BiPy

Classical Glaser-Hay conditions employ CuCl and tetramethylethylenediamine (**TMEDA**) as coupling reagents for homocoupling reaction. The reaction proceeds in a dry air atmosphere in dry CH_2Cl_2 at 20 °C.[7–9] However, coordination of the nitrogen-containing ligand to the porphyrin could disrupt the formation of the porphyrin substrate-template complex. The UV-vis-NIR formation titration of Zn porphyrin monomer with **TMEDA** (**Figure S1-left**) revealed significant binding K_f (**TMEDA**) = (1.9 ± 0.2) × 10^4 M^{-1} which is almost as strong as the binding of pyridine to the Zn monomer, K_{py} = (2.8 ± 0.2) × 10^4 M^{-1} (see the binding constant of pyridine with the Zn monomer in **Section D3**). 2,2′-Bipyridine (**2,2′-BiPy**) is also known to act as a ligand in this reaction[8,9] and the titration shows it binds less strongly to zinc porphyrins, K_f (**2,2′-BiPy**) = 5.6 ± 0.3 M^{-1} because of a steric clash between the porphyrin and the pyridyl substituent upon coordination (**Figure S1-right**). The use of **2,2′-BiPy** rather than **TMEDA** gives a larger template effect on coupling reactions; the CuCl and **TMEDA** reaction gives faster coupling but **TMEDA** binds to Zn-porphyrins four orders of magnitude more strongly than **2,2′-BiPy**. Although the reaction is slower, the **2,2′-BiPy** ligand also facilitates coupling, and thus is chosen for this work.

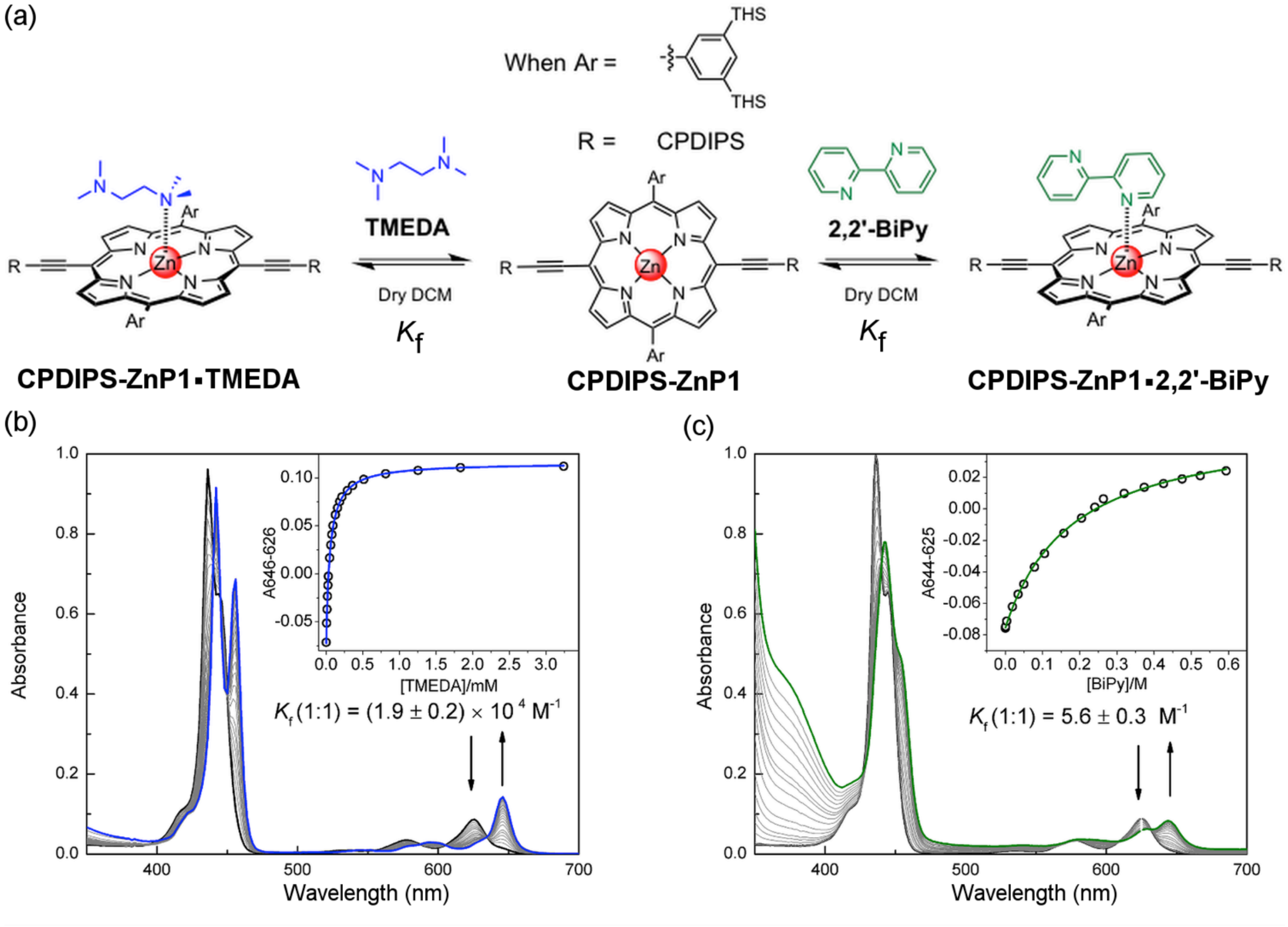

Figure S1 UV-vis-NIR formation titrations of **CPDIPS-ZnP1** ([**CPDIPS-ZnP1**] = 2.12 × 10^{-6} M) with two different amine bases: **TMEDA** and **2,2′-BiPy** in dry CH_2Cl_2 (DCM) at 298 K. (a) (left) Formation of **TMEDA** and Zn porphyrin monomer complex. (right) Formation of **2,2′-BiPy** and Zn porphyrin monomer complex. (b) Changes in absorption upon addition of **TMEDA**; (inset) binding isotherm (open dots) derived from the absorption data at λ = 646–626 nm and fit obtained from Origin™ (blue line). R^2 = 0.9998. (c) Changes in absorption upon addition of **2,2′-BiPy**; (inset) binding isotherm (open dots) derived from the absorption data at λ = 644–625 nm and fit obtained from Origin™ (green line). R^2 = 0.9987. Arrows indicate area of increasing and decreasing absorption during titration.

D. Binding studies of molecular ladder complexes

Effective molarity (*EM*) is a key factor to determine the stability and cooperativity of ladder formation. *EM*s for both 2- and 4-rung ladder complexes were determined and compared, which require the binding constants for formation of both ladder complexes to be determined.

D1. Determination of the binding constant of the 2-rung ladder complex

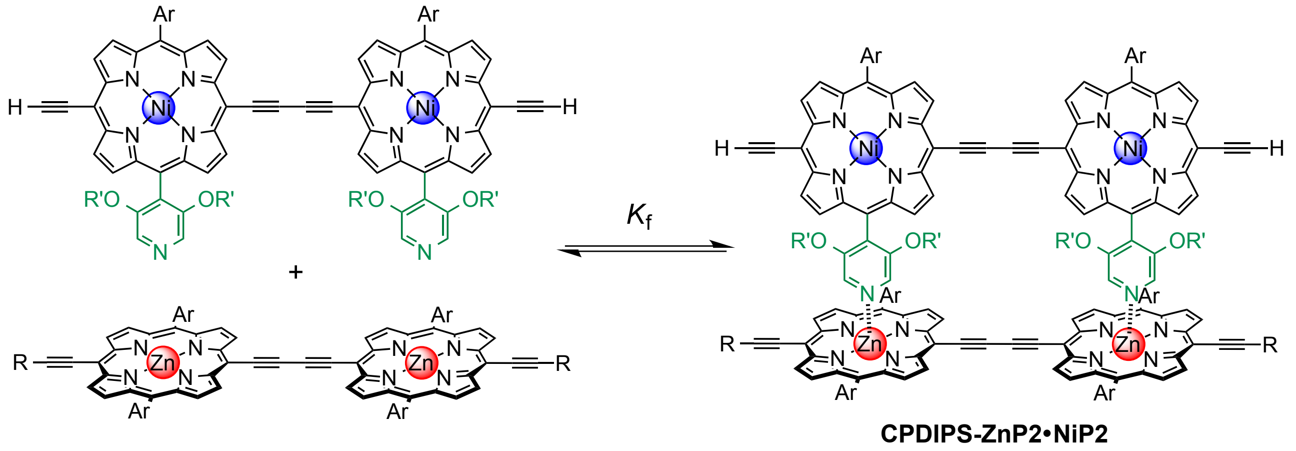

Figure S2 Formation of **CPDIPS-ZnP2•NiP2** complex. R = (3-cyanopropyl)dimethylsilyl, R′ = dodecyl. Ar = 3,5-bis(trihexylsilyl)phenyl.

The binding constant of the **CPDIPS-ZnP2•NiP2** complex was elucidated as a reference in comparison to the binding constant of the **CPDIPS-ZnP4•NiP4** complex. The UV-vis-NIR formation titration of **CPDIPS-ZnP2** with **NiP2** was first performed (**Figure S3**). The binding isotherm of dimer-dimer formation gave a sharp end point at 1:1 ratio of **NiP2** to **CPDIPS-ZnP2**, confirming the 1:1 stoichiometry of the complex (**Figure S3**).

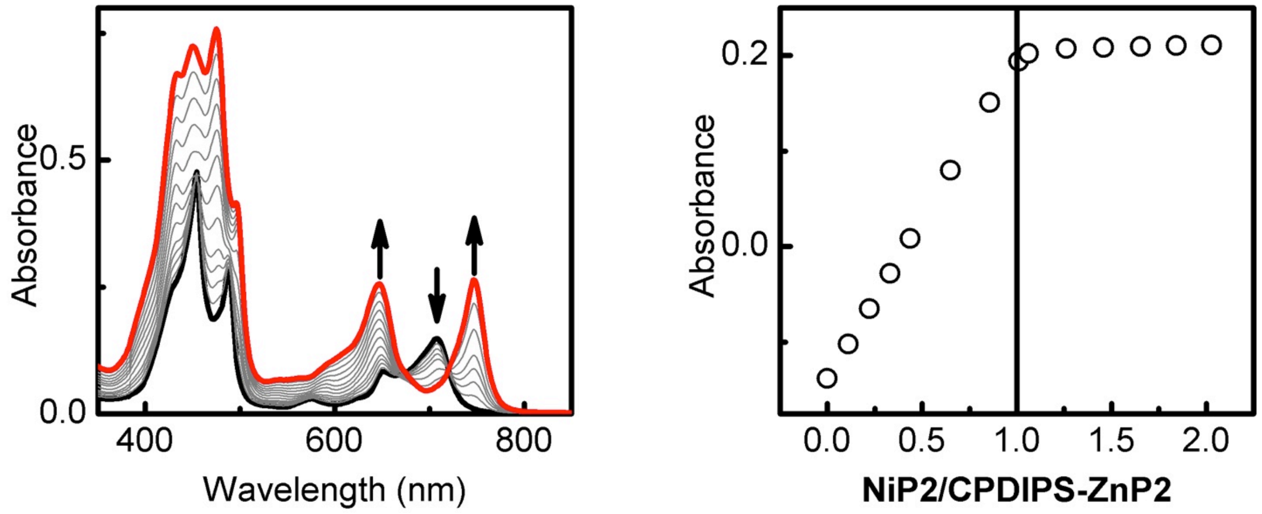

Figure S3 UV-vis-NIR formation titration of **CPDIPS-ZnP2** ([**CPDIPS-ZnP2**] = 1.41 × 10^{-6} M) with **NiP2** in dry CH_2Cl_2 at 298 K. (left) Changes in absorption upon addition of **NiP2**. Arrows indicate areas of increasing and decreasing absorption during the titration; (right) Binding isotherm derived from absorption data at 747–707 nm. The vertical line indicates a 1 to 1 ratio of **NiP2** to **CPDIPS-ZnP2**.

This complex is too stable ($K_f > 10^8$ M^{-1}) due to the square-binding isotherm (**Figure S3-right**), which does not allow K_f to be determined. Thus, the formation constant, K_f can be determined indirectly via the generic thermodynamic cycle in **Figure S4** using the following equation:

$$K_f = \frac{K_{py}^N}{K_{dn}}$$ (eq. S.1)

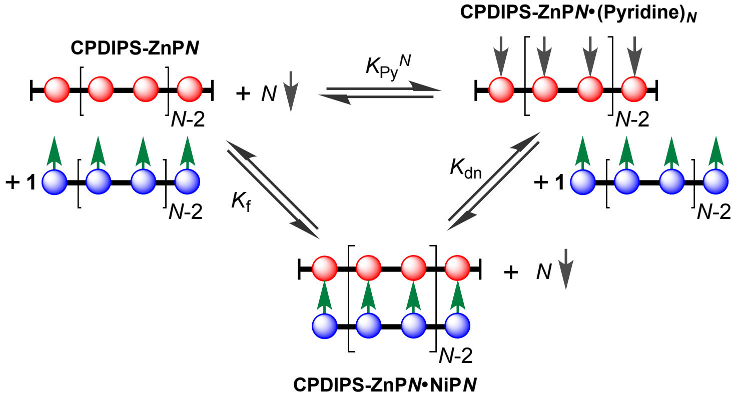

Figure S4 Thermodynamic cycle relating the formation constant of the template complex (K_f) to the denaturation constant (K_{dn}) and binding constant of each porphyrin unit for pyridine (K_{py}). The excess of pyridine was added to displace the multidentate ligand **NiP*N*** from **CPDIPS-ZnP*N*** to generate (**CPDIPS-ZnP*N***)•(**Pyridine**)$_N$.

According to **Figure S4** and **Equation S.1**, in order to acquire K_f, the denaturation constant (K_{dn}) and the reference constant (K_{py}) are necessary. The former is determined by the denaturation titration as shown below and the latter is described in **Section D3**.

The denaturation titration of **CPDIPS-ZnP2•NiP2** with pyridine was analyzed and the equilibrium can be treated as the displacement of a two-site ligand **L** from a two-site receptor **P**, with the initial concentration of **PL** being, [**P**]$_0$.

PL + 2**Py** ⇆ **PPy$_2$** + **L**

$$K_{dn} = \frac{[\mathbf{PPy_2}][\mathbf{L}]}{[\mathbf{PL}][\mathbf{Py}]^2}$$ (eq. S.2)

The denaturation constant, K_{dn} of **CPDIPS-ZnP2•NiP2** complex was defined by **Equation S.2** and was evaluated by fitting the binding isotherm to **Equation S.3**.

$$\frac{A - A_0}{A_f - A_0} = \frac{-K_{dn}[\mathbf{Py}]^N + \sqrt{K_{dn}^2[\mathbf{Py}]^{2N} + 4K_{dn}[\mathbf{Py}]^N[\mathbf{P}]_0}}{2[\mathbf{P}]_0}$$ (eq. S.3)

where A is the observed absorption at a specific wavelength or difference of absorption at two wavelengths; A_0 is the starting absorption at this wavelength or difference of absorption in these two wavelengths; A_f is the asymptotic final absorption at this wavelength or difference of absorption in these two wavelengths; [**Py**] is the concentration of pyridine and [**P**]$_0$ is the total concentration of **CPDIPS-ZnP2•NiP2** complex. N is the number of binding sites (N = 2). **Equation S.3** makes the approximation that [**Py**] ≈ total pyridine concentration [**Py**]$_0$ which is valid when [**Py**]$_0$ >> [**P**]$_0$.

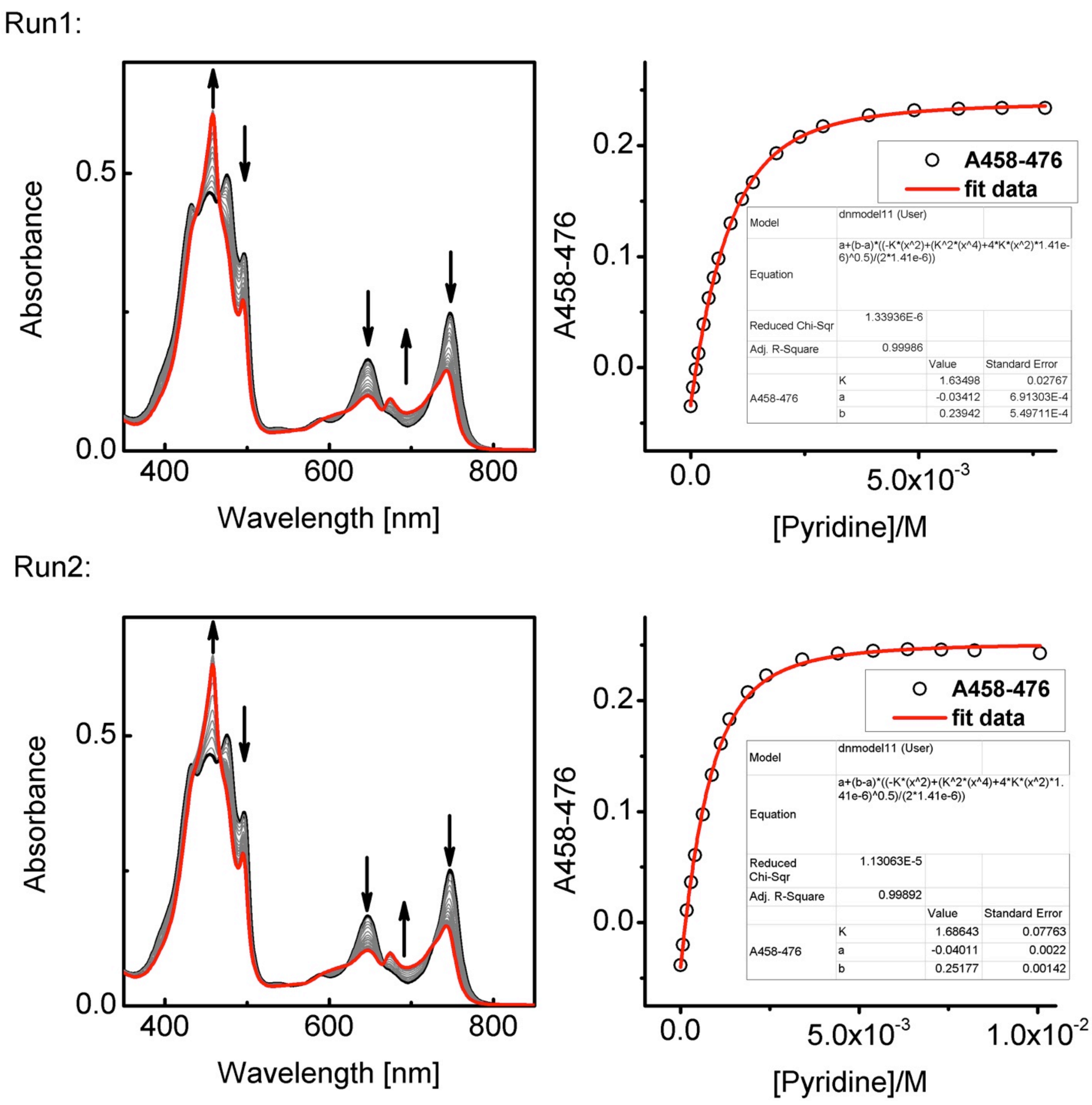

Figure S5. UV-vis-NIR denaturation titration of **CPDIPS-ZnP2•NiP2** ([complex] = 1.41 × 10^{-6} M) with pyridine in dry CH_2Cl_2 at 298 K. (left) Changes in absorption upon addition of pyridine. Arrows indicate areas of increasing and decreasing absorption during the titration; (right) Binding isotherm (open dots) derived from absorption data at λ = 458–476 nm and fit with **Equation S.3** obtained from Origin™ (red line) (Run 1: R^2 = 0.999; Run2: R^2 = 0.999).

The average value of K_{dn} obtained from fitting the binding isotherms of two titrations to a 2-site binding model for the breakup of **CPDIPS-ZnP2•NiP2** is K_{dn} = 1.66 ± 0.04 M^{-1} (**Figure S5**). The error was estimated from two replicates.

The 1:1 formation constant, K_f for **CPDIPS-ZnP2•NiP2** was calculated from K_{dn} = 1.66 ± 0.04 M^{-1} and K_{py} = (2.88 ± 0.21) × 10^4 M^{-1} (see detail of K_{py} in determination of reference constant **Section D3**) using **Equation S.1** with N = 2, giving K_f = (5.00 ± 0.74) × 10^8 M^{-1}. This K_f value will be used to calculate the effective molarity in **Section D4**.

D2. Determination of the binding constant of the 4-rung ladder complex

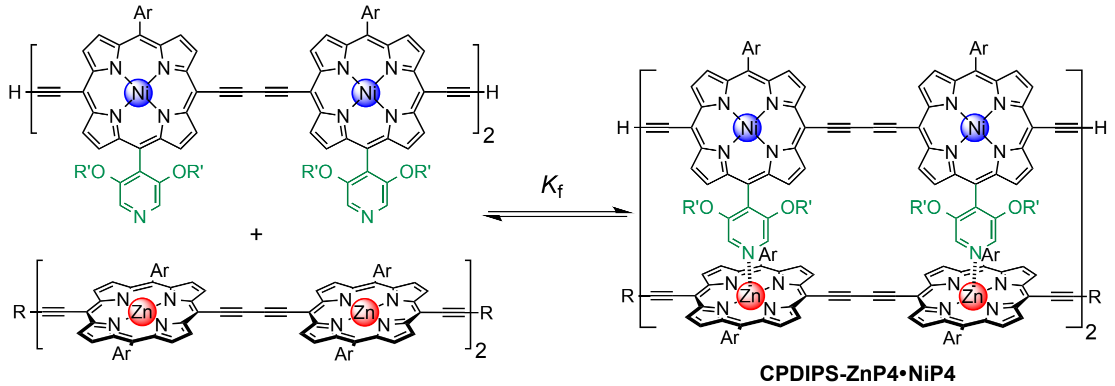

CPDIPS-ZnP4•NiP4

Figure S6. Formation of **CPDIPS-ZnP4•NiP4** complex. R = (3-cyanopropyl)dimethylsilyl, R′ = dodecyl. Ar = 3,5-bis(trihexylsilyl)phenyl.

The binding strength of the 4-rung ladder complex between **NiP4** and **CPDIPS-ZnP4** was studied to provide a measure of the stability and cooperativity of ladder formation (**Figure S6**). The UV-vis-NIR formation titration was performed at a constant micromolar concentration of **CPDIPS-ZnP4** in dry CH_2Cl_2 at 25 °C (**Figure S7**). Upon addition of **NiP4**, the Q-band absorption of **ZnP4** red-shifts and is sharpened as a consequence of coplanarization of the porphyrin units, leading to stronger electronic coupling among the units.[4,10,11] The binding isotherm is essentially linear and reaches an abrupt end-point after addition of 1 equivalent of the ligand **NiP4**, consistent with the 1:1 stoichiometry of the ladder complex **NiP4•CPDIPS-ZnP4.** As in the previous section, the stability constant is too high to determine directly from the formation curve, but it can be evaluated indirectly by denaturation with pyridine via thermodynamic cycle (**Figure S4**).

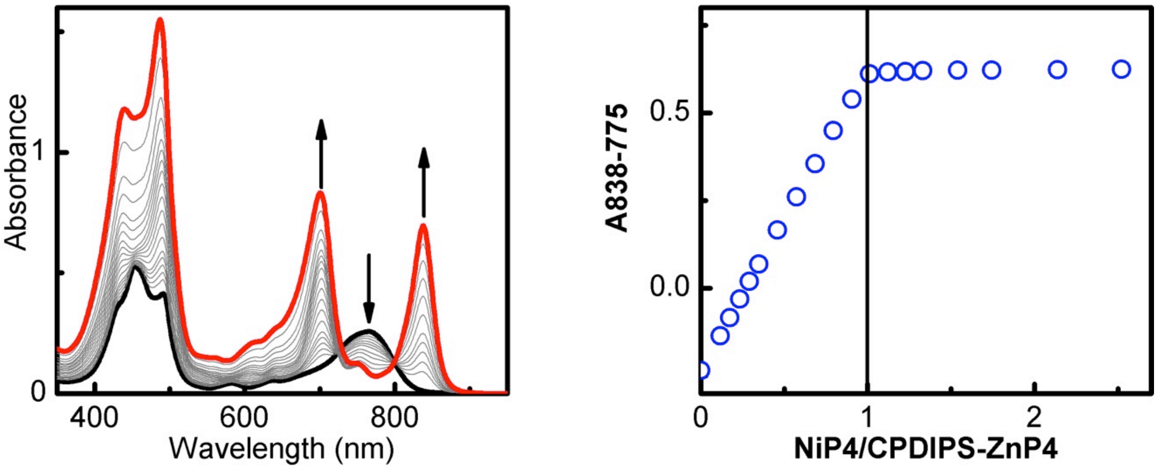

Figure S7 UV-vis-NIR formation titration of **CPDIPS-ZnP4** ([**CPDIPS-ZnP4**] = 1.11 × 10^{-6} M) with **NiP4** in dry CH_2Cl_2 at 298 K. (left) Changes in absorption upon addition of **NiP4**. Arrows indicate areas of increasing and decreasing absorption during the titration; (right) Binding isotherm derived from absorption data at 838–775 nm. The vertical line indicates a 1 to 1 ratio of **NiP4** to **CPDIPS-ZnP4**.

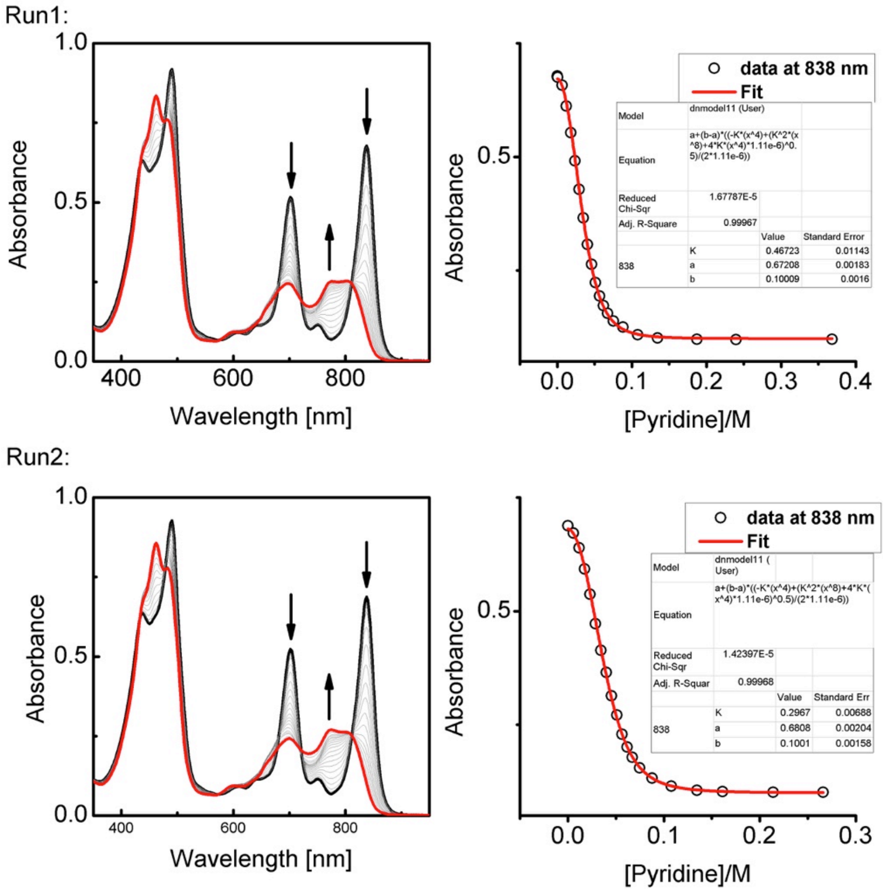

Figure S8 UV-vis-NIR denaturation titration of **CPDIPS-ZnP4•NiP4** ([complex] = 1.11×10^{-6} M) with pyridine in dry CH_2Cl_2 at 298 K. (left) Changes in absorption upon addition of pyridine; the spectrum of the initial complex (thick black line) and the final spectra of the pyridine-saturated Zn tetramer and unbound Ni tetramer (red line). Arrows indicate areas of increasing and decreasing absorption during the titration; (right) Binding isotherm (open dots) derived from absorption data at λ = 838 nm and fit with **Equation S.3** obtained from Origin™ (red line) (Run1: $R^2 = 0.9997$; Run2: $R^2 = 0.9997$).

Under the same conditions as the formation titration, a large excess of pyridine was titrated to the 4-rung ladder complex to displace the tetradentate ligand **NiP4**. The denaturation titration shows that the absorption spectra of the **NiP4•CPDIPS-ZnP4** complex are red-shifted and sharper in Q-bands compared to the pyridine bound **CPDIPS-ZnP4** at the end of titration due to the more rigid structure and coplanarization between porphyrin units in the ladder complex (**Figure S8**).[11,12] Observation of several isosbestic points in the denaturation titration and the sigmoidal binding isotherm indicate an all-or-nothing two-state equilibrium (i.e., that partially denatured species are not populated) (**Figure S8-left**).[12–15]

The binding constant, K_f of the **CPDIPS-ZnP4•NiP4** complex was determined via the denaturation titration of **CPDIPS-ZnP4•NiP4** with excess pyridine can be analyzed as a two-equilibrium state, where PL is **CPDIPS-ZnP4•NiP4** and **L** is **NiP4**.

$$\mathbf{PL} + 4\mathbf{Py} \leftrightarrows \mathbf{PPy_4} + \mathbf{L}$$

$$K_{dn} = \frac{[\mathbf{PPy_4}][\mathbf{L}]}{[\mathbf{PL}][\mathbf{Py}]^4} \qquad \textbf{(eq. S.4)}$$

The denaturation constant, K_{dn}, defined by **Equation S.4** was determined by fitting the binding isotherm to **Equation S.3** with $N = 4$.

The average value of K_{dn} obtained from fitting the binding isotherms of two titrations to a 4-site binding model for the breakup of **CPDIPS-ZnP4•NiP4** is $K_{dn} = 0.39 \pm 0.06$ M^{-3} (**Figure S8-right**). The error was estimated from two replicates. The 1:1 formation constant, K_f for **CPDIPS-ZnP4•NiP4** was calculated via

the generic thermodynamic cycle using **Equation S.1** with $N = 4$, giving $K_f = (1.8 \pm 0.6) \times 10^{18}$ M^{-1}. This K_f value will be used to calculate the effective molarity in **Section D4**.

D3. Determination of reference constant

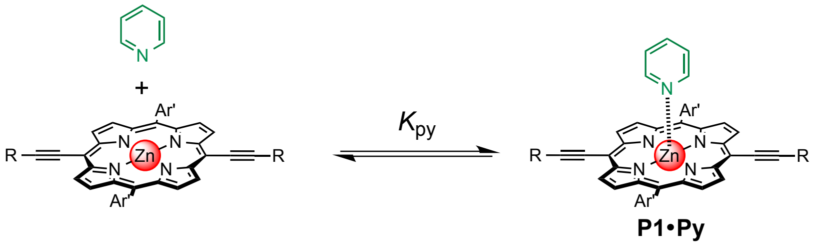

Figure S9 Formation of pyridine and porphyrin monomer complex (**P1•Py**). Ar′ = 3,5-bis(trihexylsilyl)phenyl, R = (3-cyanopropyl)dimethylsilyl.

The binding constant of pyridine to Zn porphyrin monomer complex (K_{py}) is needed to derive the binding constants (K_f) of the **CPDIPS-ZnP2•NiP2** and **CPDIPS-ZnP4•NiP4** complexes from the thermodynamic cycle. K_{py} was obtained by UV-vis-NIR formation titration of Zn porphyrin monomer **CPDIPS-ZnP1** with pyridine (**Figure S9**).

The experimental data were in excellent agreement with the theoretically derived 1:1 formation binding mode, resulting in average $K_{py} = (2.88 \pm 0.21) \times 10^4$ M^{-1} (**Figure S10**). The error was estimated from two replicates.

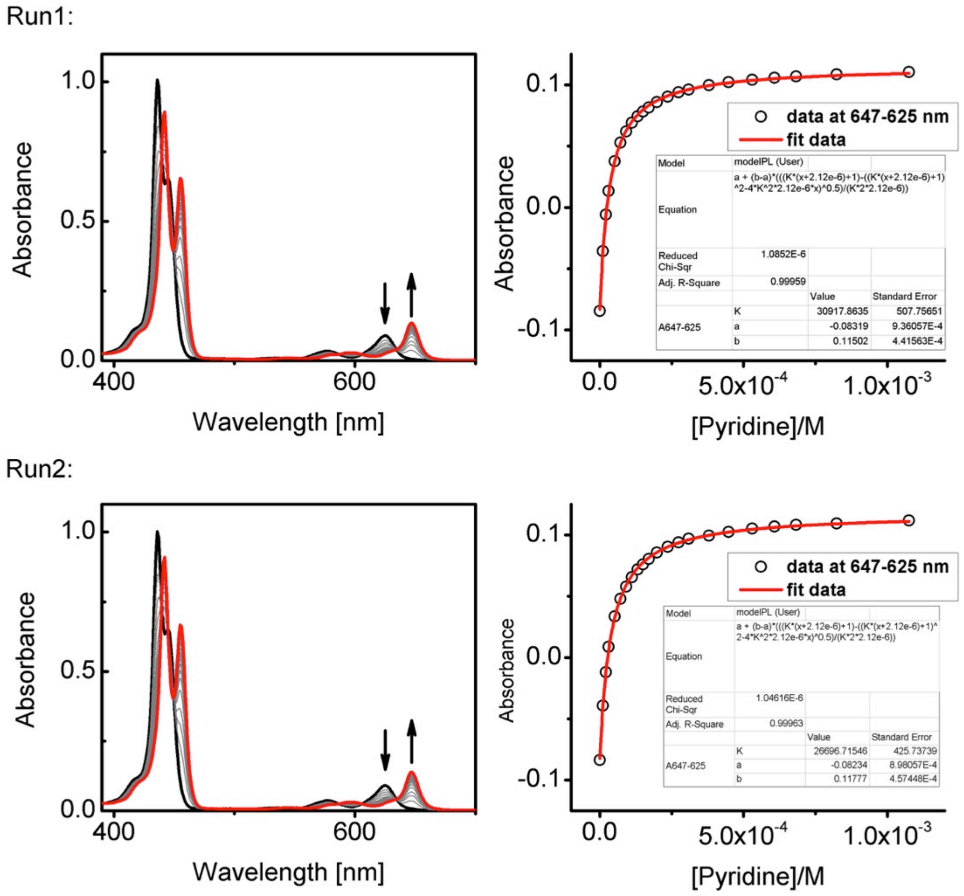

Figure S10 UV-vis-NIR formation titration of **CPDIPS-ZnP1** ([**CPDIPS-ZnP1**] = 2.12×10^{-6} M) with pyridine in dry CH_2Cl_2 at 298 K; average $K_{py} = (2.88 \pm 0.21) \times 10^4$ M^{-1} from two runs. (left) Changes in absorption upon addition of pyridine; the spectra of the Zn monomer (thick black line) and the end spectra of the pyridine-saturated Zn monomer (red line). Arrows indicate areas of increasing and decreasing absorption during the titration; (right) Binding isotherm (black dots) derived from absorption data at λ = 647– 625 nm and fit obtained from Origin™ (red line) (Run 1, R^2 = 0.9996; Run 2, R^2 = 0.9996).

D4. Calculation of effective molarities of 2- and 4-rung ladder complexes

The complementarity between the two strands of the 4-rung ladder complex **NiP4•CPDIPS-ZnP4** can be evaluated by effective molarity, in comparison to the one for the 2-rung ladder complex **NiP2•CPDIPS-ZnP2**.[13,14] The binding strength of the 4-rung ladder complex from the previous section is very high which could lead to the strong *EM*.[15]

The effective molarities of **CPDIPS-ZnP2•NiP2** (EM_1) and **CPDIPS-ZnP4•NiP4** (EM_2) can be calculated using the equations in **Figure S11a** and **b**, respectively. The statistical factors for equilibria as shown in **Figure S11** are evaluated from symmetry numbers as explained in Ref. 14. The chemical binding constant $K_{chem,1}$ was estimated by dividing the binding constant for coordination of pyridine to Zn porphyrin monomer **CPDIPS-ZnP1** K_1 by the statistical factor ($K_\sigma = 2$), providing $K_{chem,1} = (1.44 \pm 0.11) \times 10^4$ M^{-1}. K_2 and K_4 were obtained from the measured binding constants of **CPDIPS-ZnP2•NiP2** ($K_2 = (5.00 \pm 0.74) \times 10^8$ M^{-1}) and **CPDIPS-ZnP4•NiP4** ($K_4 = (1.79 \pm 0.60) \times 10^{18}$ M^{-1}) from denaturation titrations in **Section D1** and **D2**, respectively. The chemical binding constant of **CPDIPS-ZnP2•NiP2** $K_{chem,2}$ was obtained by dividing K_2 by the statistical factor ($K_\sigma = 8$), giving $K_{chem,2} = (6.24 \pm 0.92) \times 10^7$ M^{-1}). With the values of $K_{chem,1}$, $K_{chem,2}$, K_2 and K_4, the effective molarities of **CPDIPS-ZnP2•NiP2** and **CPDIPS-ZnP4•NiP4** can be given by $EM_1 = 0.3 \pm 0.1$ M and $EM_2 = 14 \pm 6$ M, respectively. For the 4-rung ladder complex, the average effective molarity ($\overline{EM}$) was also determined using the equation in **Figure S11c**, providing $\overline{EM} = 1.1 \pm 0.2$ M.

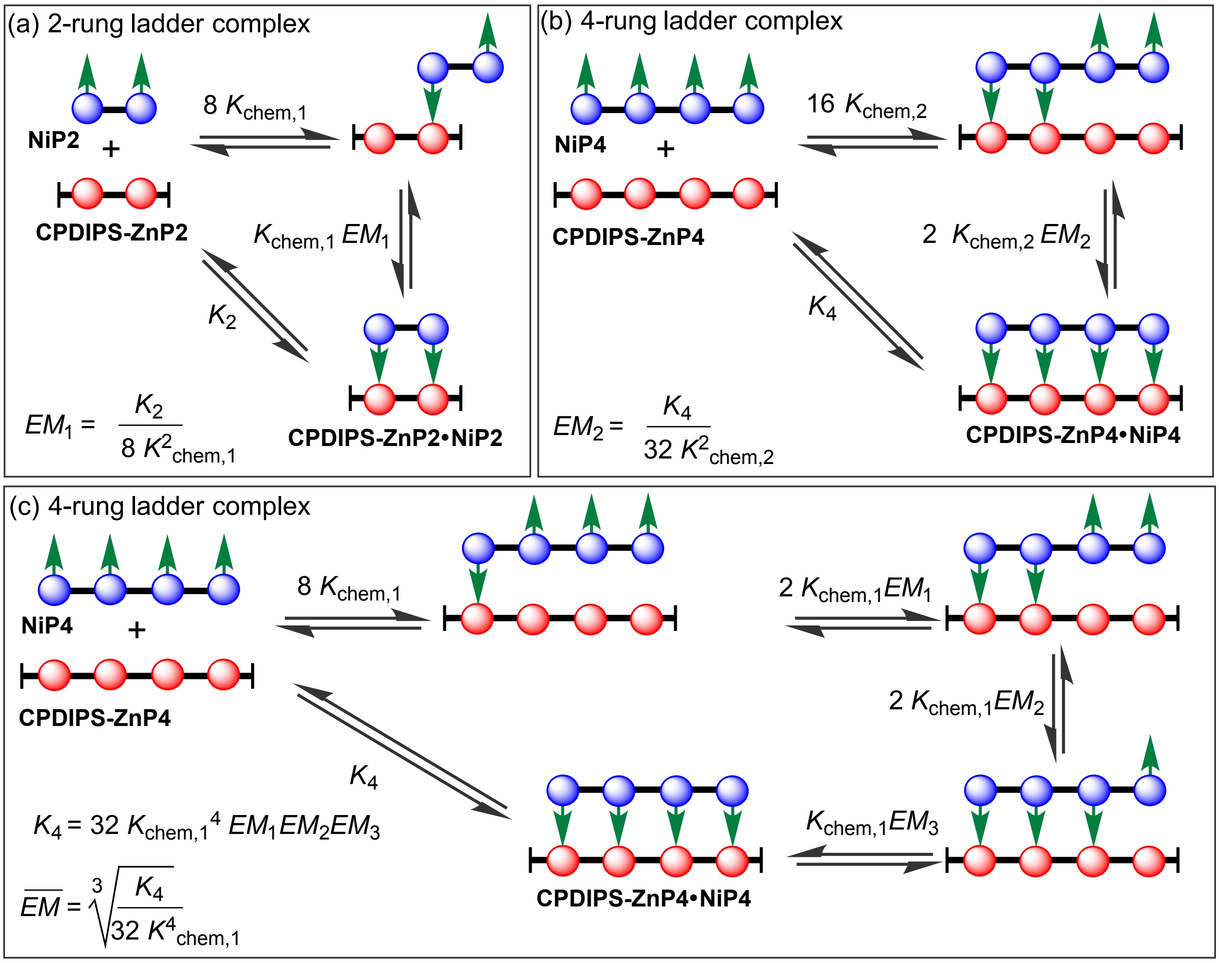

Figure S11 Self-assembly of (a) **CPDIPS-ZnP2•NiP2**, (b) and (c) **CPDIPS-ZnP4•NiP4** complexes.

It is remarkable that increasing the number of coordination sites within the structure by a factor of two enhances the cooperative effect by almost a factor of 50, reflecting the preorganized structure and increase in rigidification of the structure with increasing molecular size. The low *EM* in the 2-rung ladder complex also reveals the flexibility of the complex. The higher cooperativity in the 4-rung ladder complex leads to the higher yield in the template-directed synthesis of linear porphyrin tetramer.

E. Molecular structures of ladder complexes

E1. ¹H NMR Characterization

The assignments of the signals in the NMR spectra of the ladder complexes were carried out by a combination of COSY and NOE spectroscopy. Due to the complexity of the ¹H NMR of the 4-rung ladder complex **CPDIPS-ZnP4•NiP4**, the 2-rung ladder complex **CPDIPS-ZnP2•NiP2** was analyzed first to gain understanding of the simplest system.

E1.1 ¹H NMR assignment of **CPDIPS-ZnP2•NiP2**

The 2-rung ladder complex **CPDIPS-ZnP2•NiP2** was prepared by titration of **CPDIPS-ZnP2** ([**CPDIPS-ZnP2**] = 0.57 mM) with **NiP2** in $CDCl_3$ at 298 K. When a 1:1 ratio of Zn dimer to Ni dimer was reached, the signals corresponding to individual components disappeared and a new product appeared to have been formed (**Figure S12**).

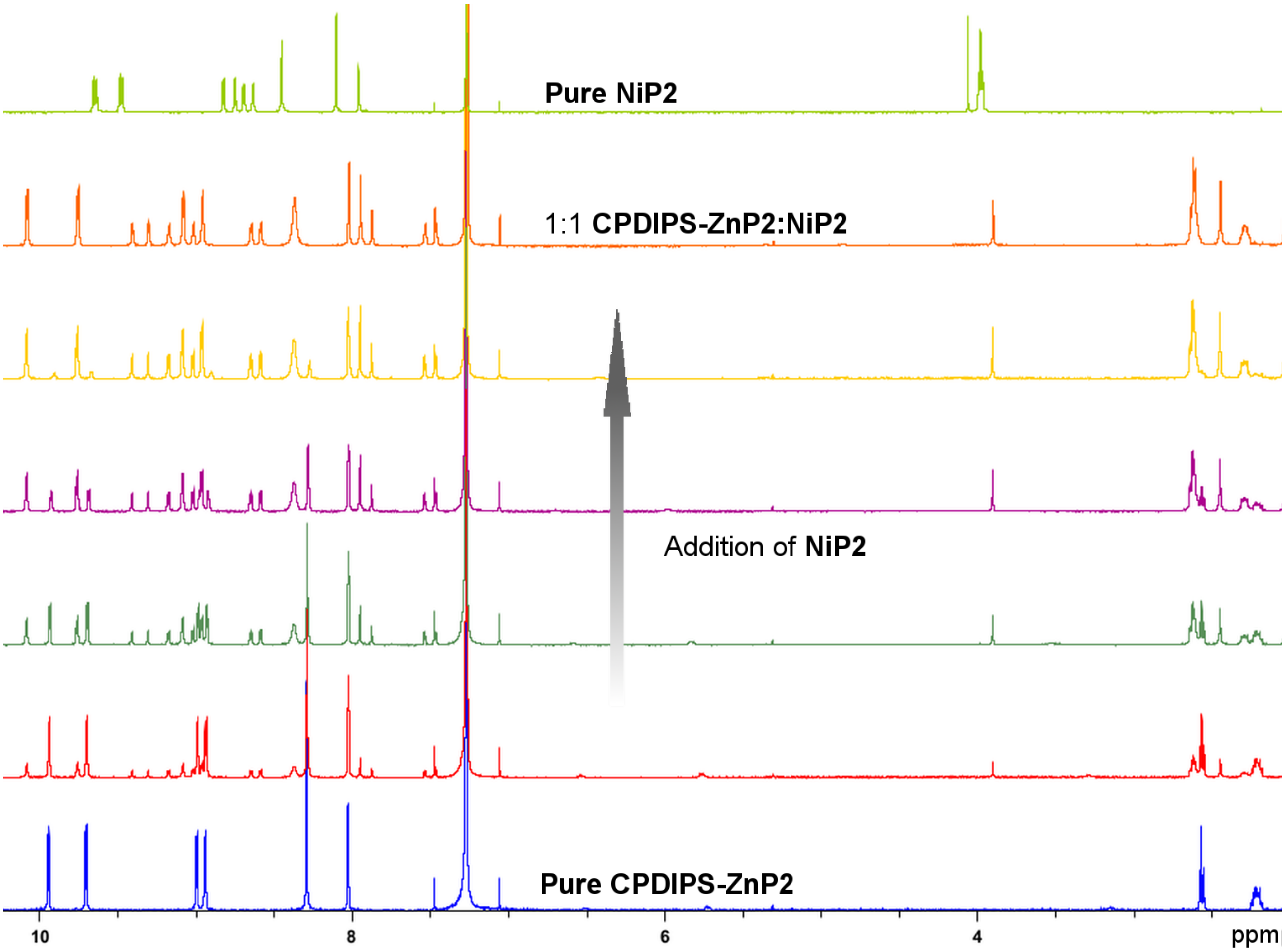

Figure S12 ¹H NMR titration of the 2-rung ladder complex of **CPDIPS-ZnP2** with **NiP2** (500 MHz, $CDCl_3$, 298 K, [**CPDIPS-ZnP2**] = 0.5 mM).

Based on the symmetry of the **CPDIPS-ZnP2•NiP2** complex, only half of the molecule needs to be considered in the interpretation of the ^{1}H NMR spectrum. The chemical structure in **Figure S13** represents a porphyrin unit of **CPDIPS-ZnP2** coordinating to a porphyrin unit of **NiP2**.

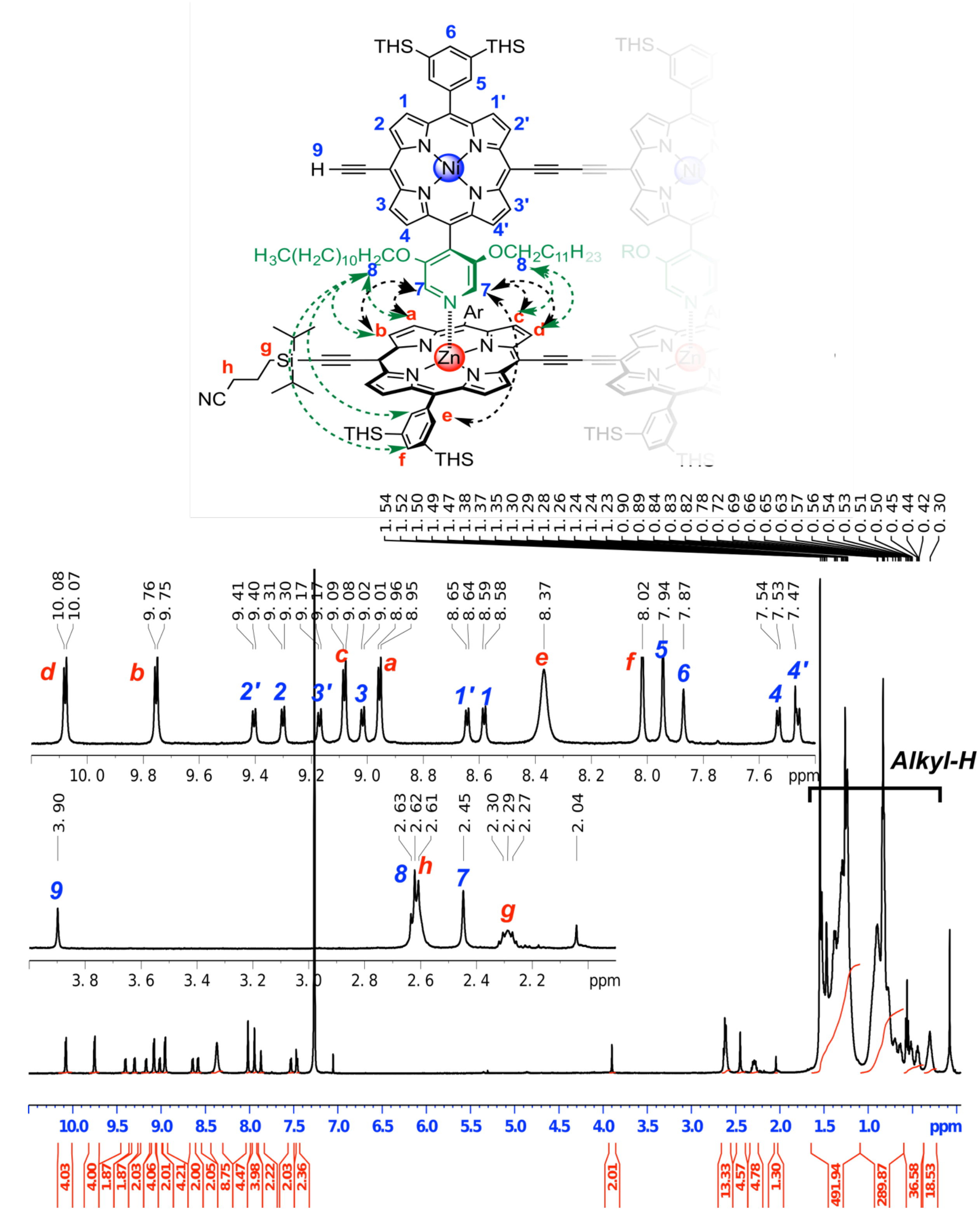

Figure S13 Representative half of the 2-rung ladder structure for the interpretation of the ^{1}H NMR spectrum (top, arrows for key NOEs) and ^{1}H NMR spectrum of **CPDIPS-ZnP2•NiP2** (bottom, 500 MHz, $CDCl_3$, 298 K).

Our assignment of the ^{1}H NMR spectrum starts with the β-protons from the Zn porphyrin dimer (**Figure S14**). Protons ***d*** and ***b*** which are adjacent to the triple bond are at the higher chemical shift than protons ***a*** and ***c***. Proton ***d*** which is inside the molecule, experiences more deshielding than proton ***b***, resulting in chemical

shift of proton ***d*** higher than ***b***. There are also correlations ***a⇔b*** and ***c⇔d*** in the COSY spectrum, confirming the close proximity of these β-protons.

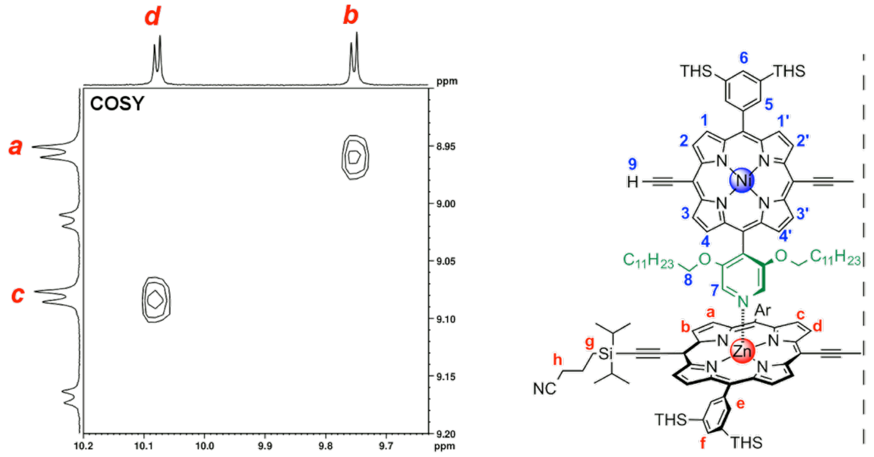

Figure S14 Region of the COSY spectrum of **CPDIPS-ZnP2•NiP2** corresponding to **CPDIPS-ZnP2** β-protons ***a***, ***b***, ***c*** and ***d*** (500 MHz, $CDCl_3$, 298 K).

As shown in **Figure S15**, β-protons ***a*** and ***c*** show strong NOEs with aryl side-group proton ***e*** and β-protons ***b*** and ***d*** show weak NOEs with proton ***e***. Proton ***e*** also has coupling with aryl side group proton ***f*** in the COSY spectrum. All protons ***a***, ***b***, ***c***, ***d***, ***e*** and ***f*** are on the Zn porphyrin dimer. Aryl rotation of the Zn porphyrin dimer interconverts two ***e*** environments, making signal of ***e*** broad.

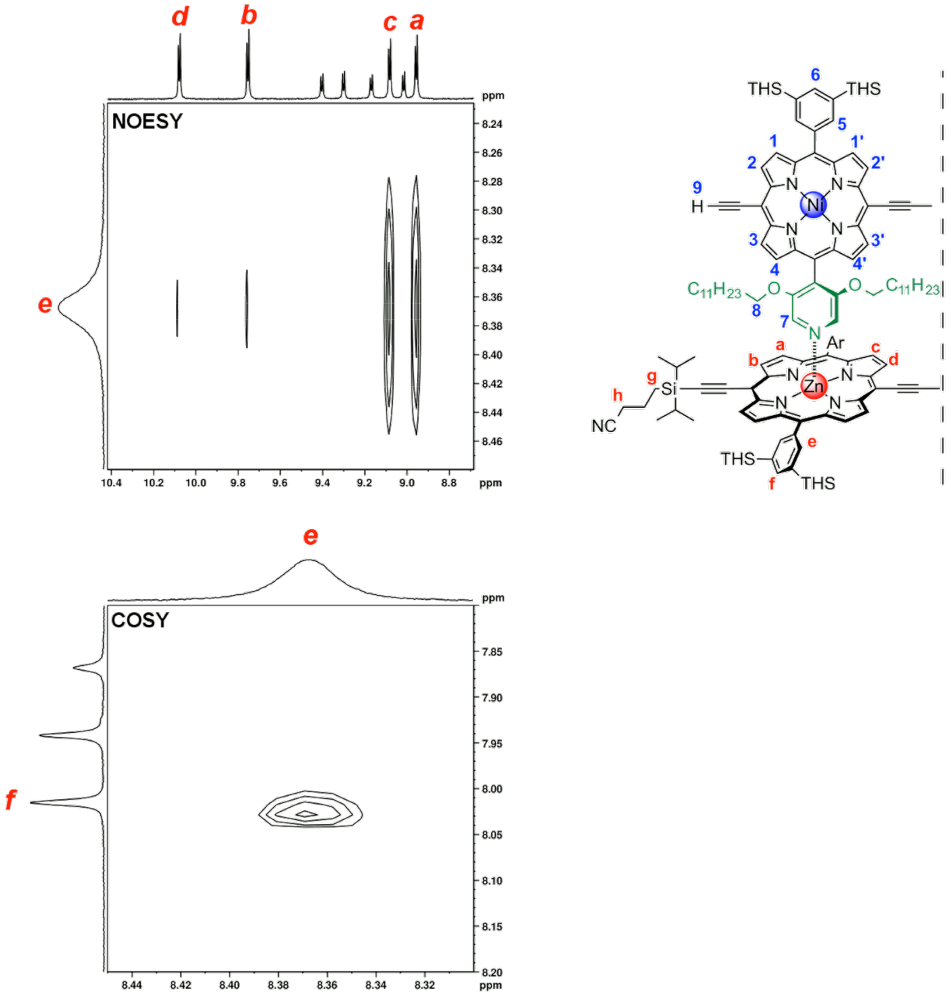

Figure S15 Region of the NOESY and COSY spectra of **CPDIPS-ZnP2•NiP2** corresponding to **CPDIPS-ZnP2** β-protons ***a***, ***b***, ***c*** and ***d*** and aryl side-group protons ***e*** and ***f*** (500 MHz, $CDCl_3$, 298 K).

Protecting group protons ***g*** and ***h*** on the Zn porphyrin dimer were assigned based on their correlation in the COSY spectrum and the relationship of NOEs between proton ***g*** and proton ***b*** (**Figure S16**).

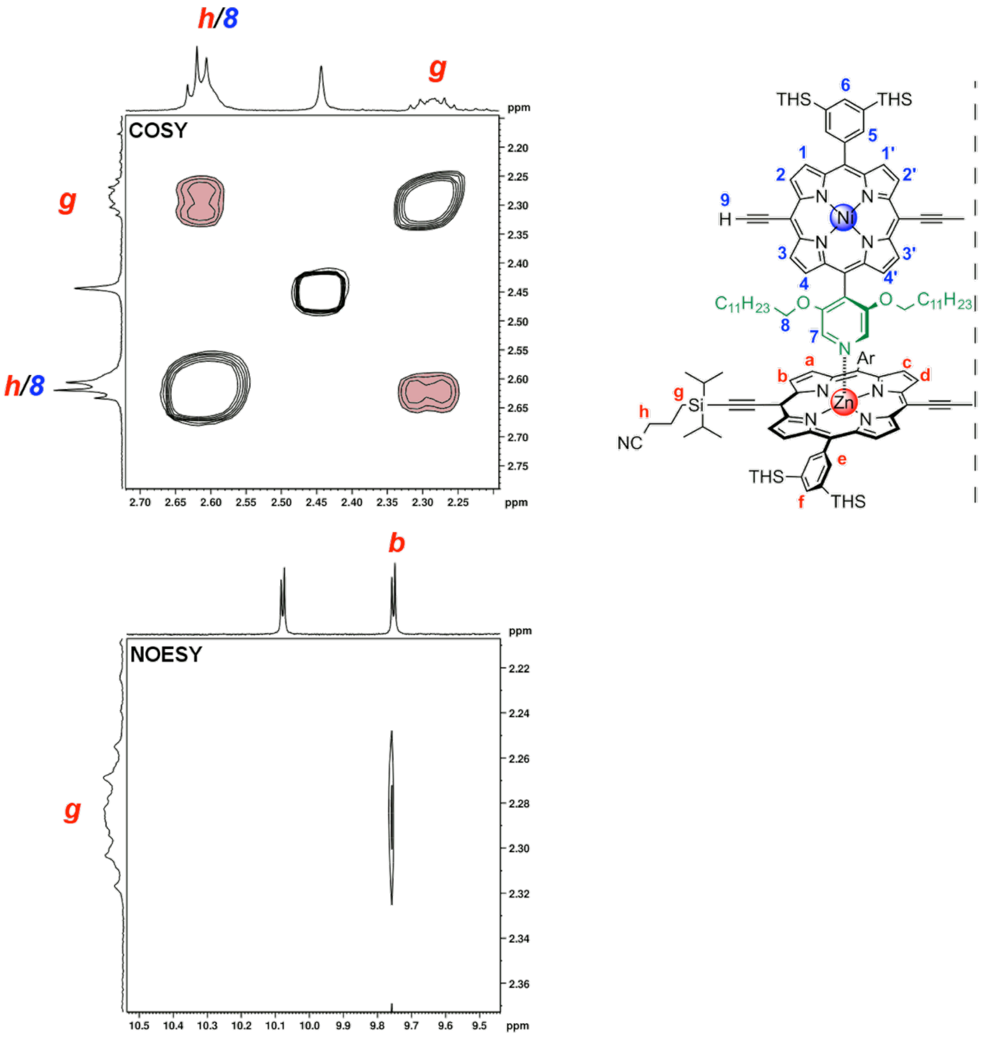

Figure S16 Region of the COSY and NOESY spectra of **CPDIPS-ZnP2•NiP2** corresponding to **CPDIPS-ZnP2** protecting group protons ***g*** and ***h*** and β-proton ***b*** (500 MHz, $CDCl_3$, 298 K). The pink color of the peaks highlights the correlation between protons ***g*** and ***h*** in the COSY spectrum.

Due to the ring current effect, the α-proton *7* on pyridyl side-groups of **NiP2** is strongly shielded and appears as a singlet signal at very low chemical shifts (2.45 ppm). The triplet signal of alkyl side-chain proton ***8*** on the pyridyl side-groups is also shielded from the ring current effect and shifted to the up-field at 2.62 ppm which is overlapping with the proton ***h*** of **CPDIPS-ZnP2**.

In the NOESY spectrum (**Figure S17**), the Zn porphyrin β-protons ***a***, ***b***, ***c***, ***d*** and ***e*** show NOEs with the Ni porphyrin pyridyl proton *7* and alkyl side-chain proton ***8*** on the pyridyl groups and there is also a NOE between proton ***f*** and proton ***8***, confirming the proximity of **CPDIPS-ZnP2** and **NiP2**.

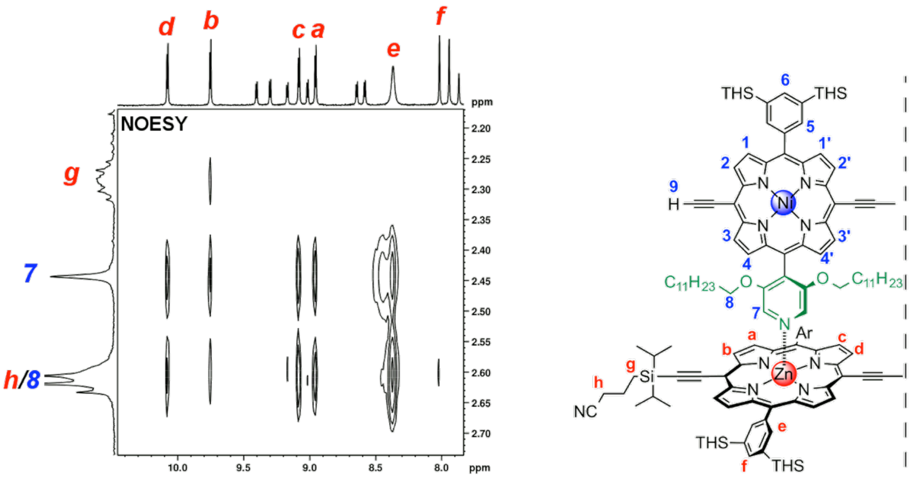

Figure S17 Region of the NOESY spectrum of **CPDIPS-ZnP2•NiP2** corresponding to **CPDIPS-ZnP2** protons ***a***, ***b***, ***c***, ***d***, ***e*** and ***f*** and **NiP2** protons *7* and ***8*** (500 MHz, $CDCl_3$, 298 K).

Of the β-protons of **NiP2**, protons ***2***, ***2'***, ***3*** and ***3'***, which are adjacent to the triple bond, are at higher chemical shift than protons ***1***, ***1'***, ***4*** and ***4'***. Inner Protons ***2'*** and ***3'*** experience a stronger deshielding effect from butadiyne bridge, resulting in a higher chemical shift than outer protons **2** and **3**, respectively.

Additionally, β-protons ***1*** and ***1'*** show NOEs with THS aryl side-group proton ***5*** and the coupling between β-protons ***1⇔2*** and ***1'⇔2'*** are also observed in the COSY spectrum (**Figure S18**). Based on these couplings and NOEs, ***1***, ***1'***, ***2*** and ***2'*** are readily assigned on the upper part of the Ni-strand.

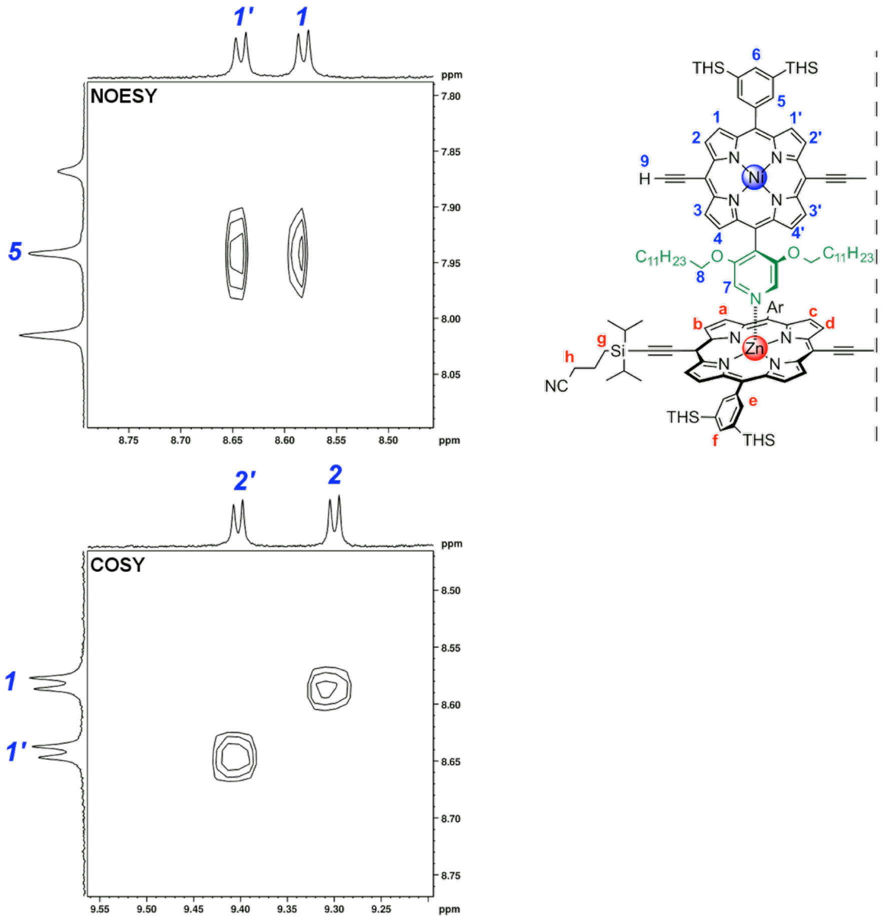

Figure S18 Region of NOESY and COSY spectra of **CPDIPS-ZnP2•NiP2** corresponding to **NiP2** β-protons ***1***, ***1'***, ***2*** and ***2'*** and THS aryl side-group proton ***5*** (500 MHz, $CDCl_3$, 298 K).

In the lower part of the Ni-strand, β-protons ***4*** and ***4'*** are more shielded by the ring current of the Zn-strand than the other β-protons ***1***, ***1'***, ***2***, ***2'***, ***3*** and ***3'*** due to the close proximity to the Zn porphyrin plane, providing the large shift at 7.54 and 7.47 ppm, respectively. Based on their NOEs with protons ***7*** and ***8***, the assignment of β-protons ***4*** and ***4'*** can be confirmed (**Figure S19 top**). The couplings between protons ***3'***⇔***4'*** and ***3***⇔***4*** are observed in the COSY spectrum (**Figure S19 bottom**). This clarifies the assignment of protons ***3*** and ***3'*** on the lower part of the Ni-strand.

The acetylene proton ***9*** is slightly shifted to the up-field since the small effect of ring currents from the Zn-strand.

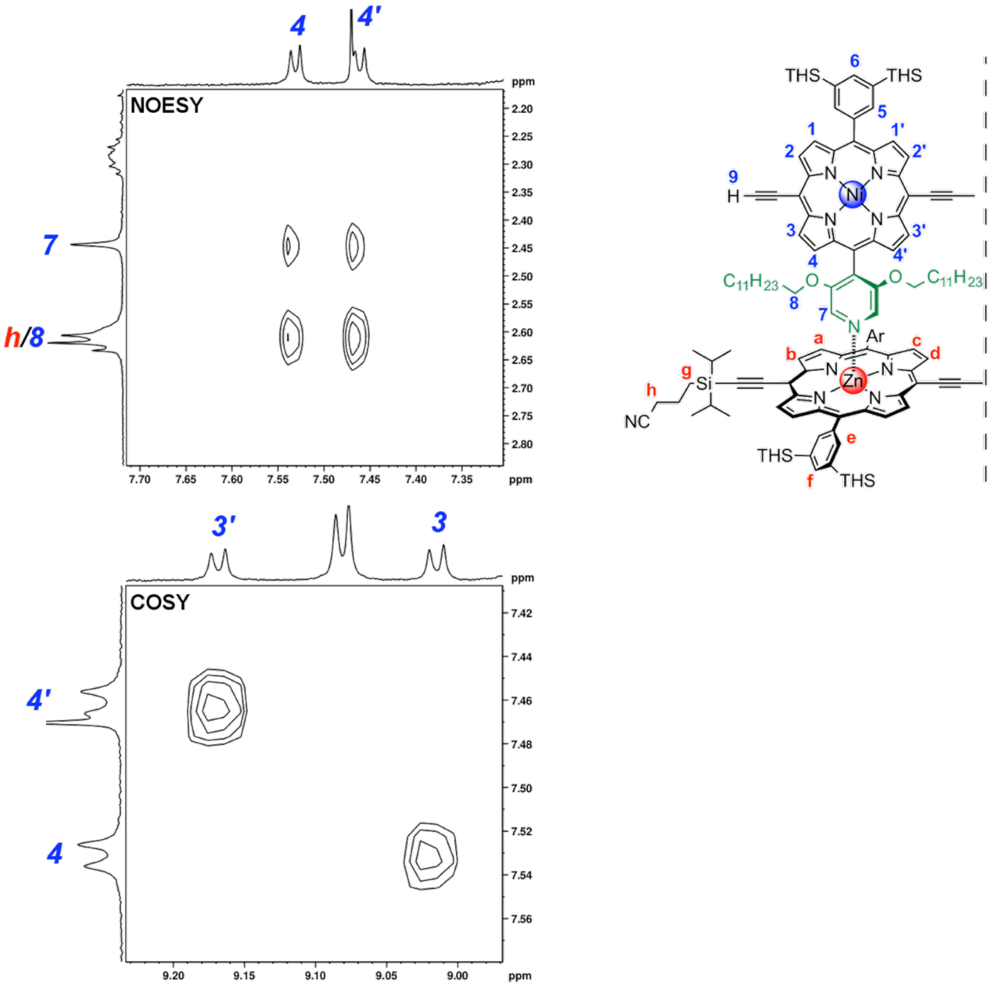

Figure S19 Region of the NOESY and COSY spectra of **CPDIPS-ZnP2•NiP2** corresponding to **NiP2** β-protons ***3***, ***3'***, ***4*** and ***4'*** and protons *7* and ***8*** (500 MHz, $CDCl_3$, 298 K).

E1.2 ¹H NMR assignment of **CPDIPS-ZnP4•NiP4**

The 4-rung ladder complex **CPDIPS-ZnP4•NiP4** was prepared by titration of a solution of **NiP4** into **CPDIPS-ZnP4** in a similar fashion to the 2-rung ladder complex (**Figure S20**).

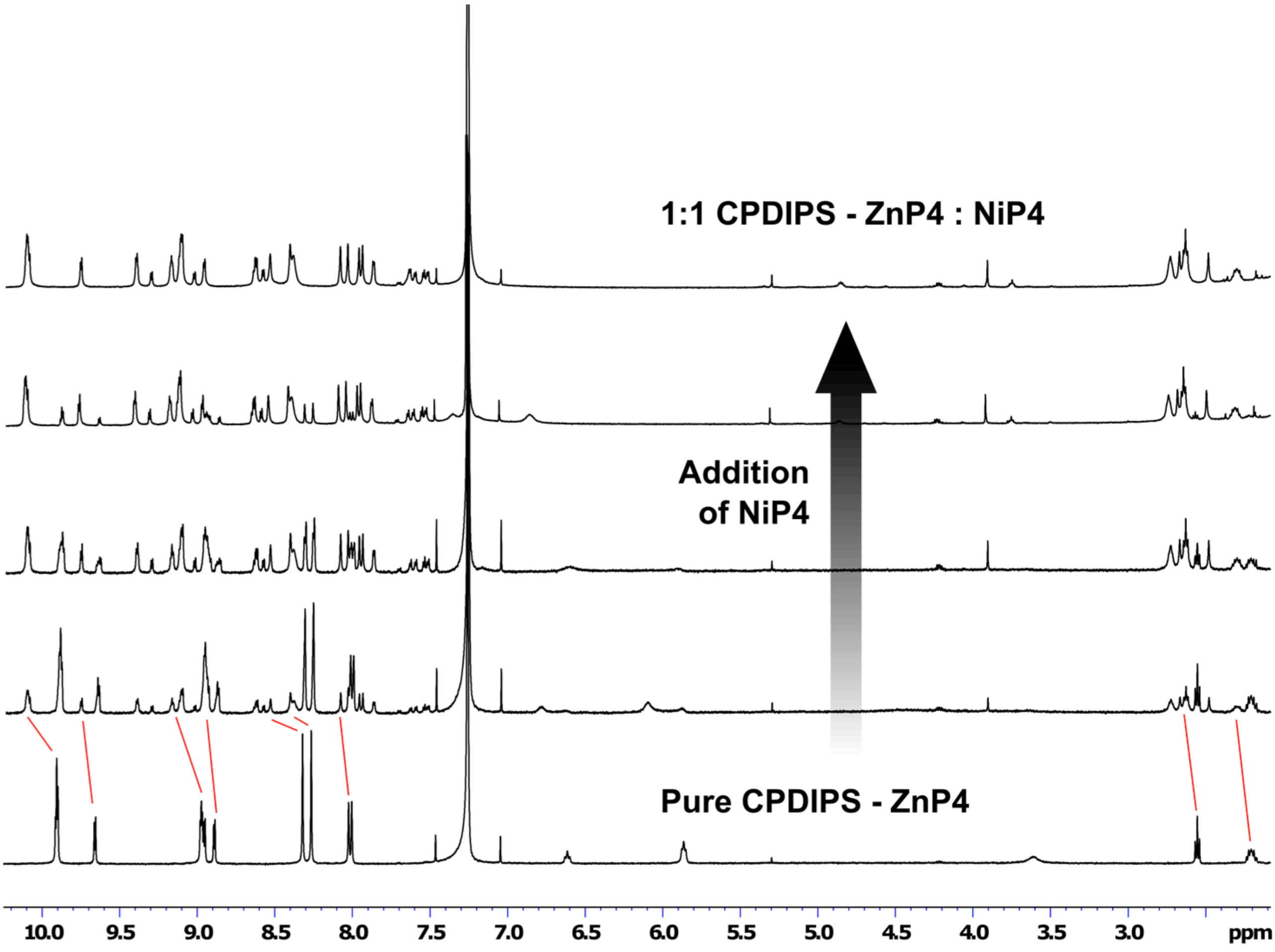

Figure S20 ¹H NMR titration of **CPDIPS-ZnP4** with **NiP4** to form the 4-rung ladder complex (500 MHz, $CDCl_3$, 298 K, [**CPDIPS-ZnP4**] = 0.22 mM).

Based on the symmetry of the **CPDIPS-ZnP4•NiP4** complex, only half of the molecule needs to be considered in the interpretation of the ^{1}H NMR spectrum. The chemical structure in **Figure S21** represents two porphyrin units of **CPDIPS-ZnP4** coordinating to two porphyrin units of **NiP4**.

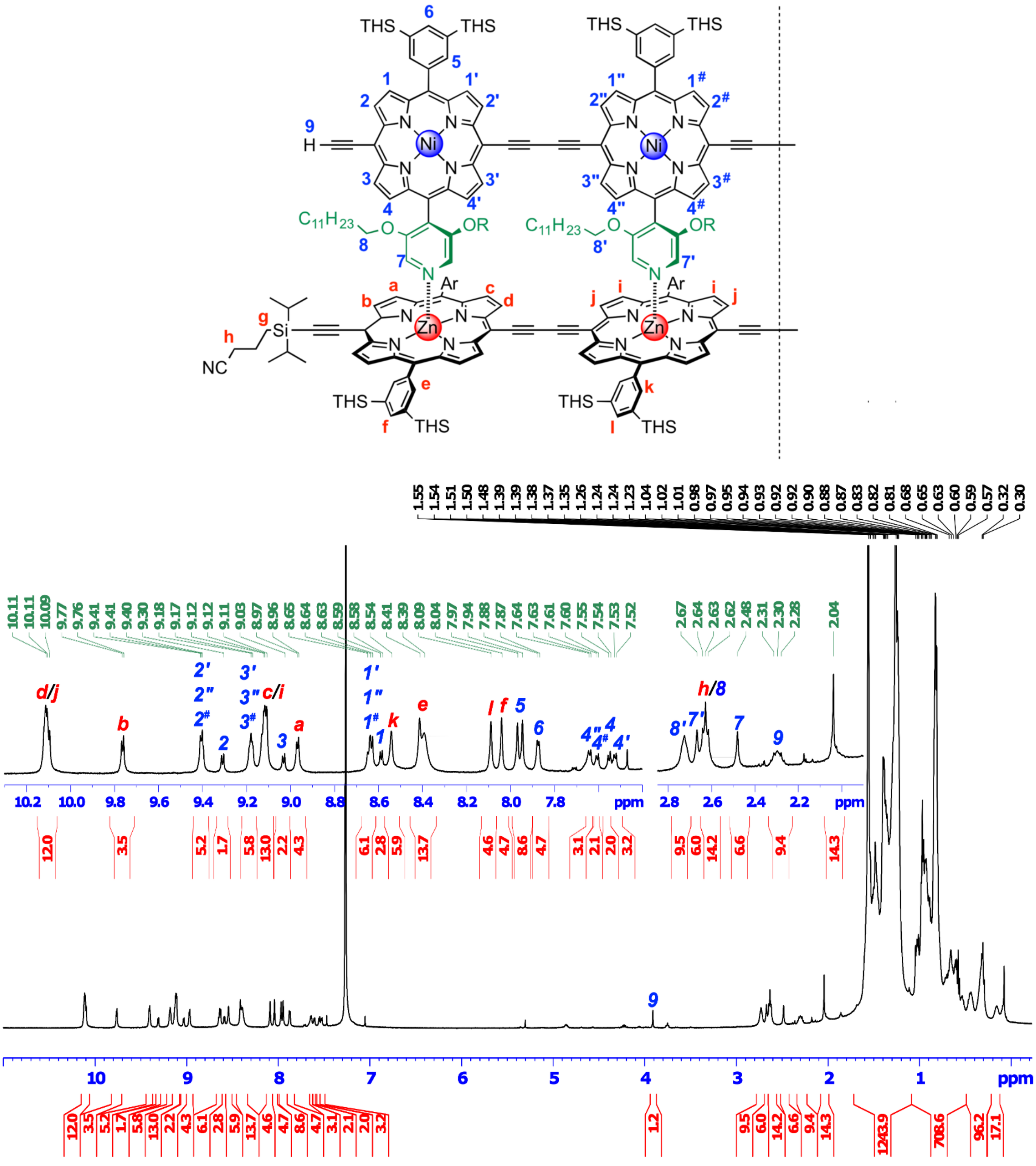

Figure S21 Representative half of the 4-rung ladder structure (top) and ^{1}H NMR spectrum of **CPDIPS-ZnP4•NiP4** (bottom, 500 MHz, $CDCl_3$, 298 K).

Our assignment of the ^{1}H NMR spectrum begins with the β-protons from **CPDIPS-ZnP4**. Proton ***d*** overlaps with proton ***j*** and proton ***c*** overlaps with proton ***i*** because they are in very similar environments. Protons ***b***, ***d*** and ***j***, which are adjacent to the triple bond, are at a higher chemical shift than protons ***a***, ***c*** and ***i***. Protons ***d*** and ***j***, which point towards another porphyrin unit, are more deshielded by the butadiyne bridge, resulting in a higher chemical shift for protons ***d*** and ***j*** than for ***b***. There are also correlations ***b***⇔***a***, ***d***⇔***c*** and ***j***⇔***i*** in the COSY spectrum, confirming the close proximity of these β-protons (**Figure S22**).

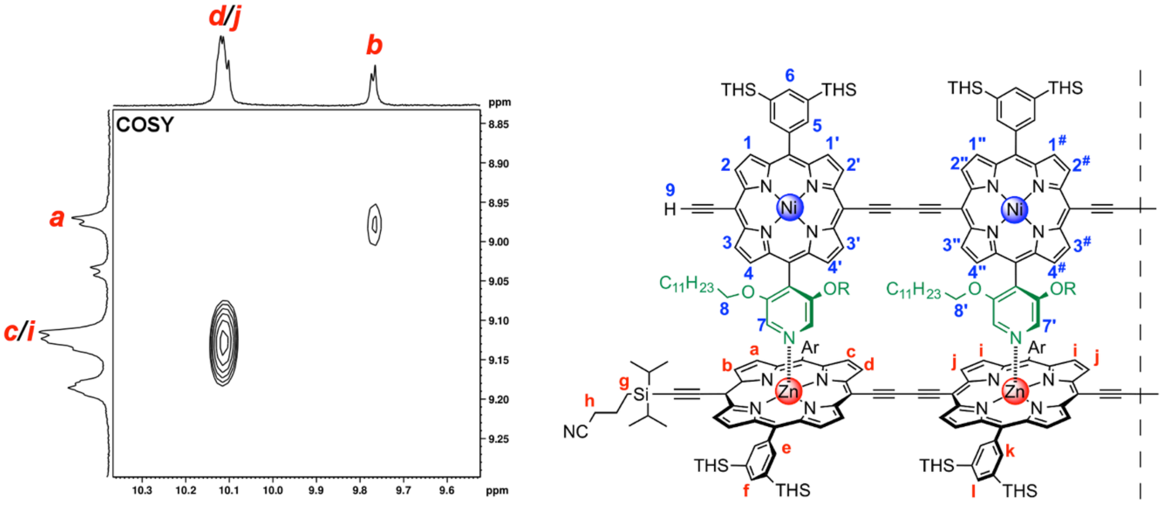

Figure S22 Region of the COSY spectrum of **CPDIPS-ZnP4•NiP4** corresponding to **CPDIPS-ZnP4** β-protons ***a***, ***b***, ***c***, ***d***, ***i*** and ***j*** (500 MHz, $CDCl_3$, 298 K).

β-protons ***a***, ***c*** and ***d*** from the outer Zn porphyrin unit also show NOEs with outer aryl side-group proton ***e*** while β-protons ***i*** and ***j*** from the inner Zn porphyrin unit show NOEs with inner aryl side-group proton ***k*** (**Figure S23**). Proton ***e*** also has coupling with aryl side-group proton ***f*** whereas proton ***k*** has coupling with proton ***l*** in COSY spectrum (**Figure S24**).

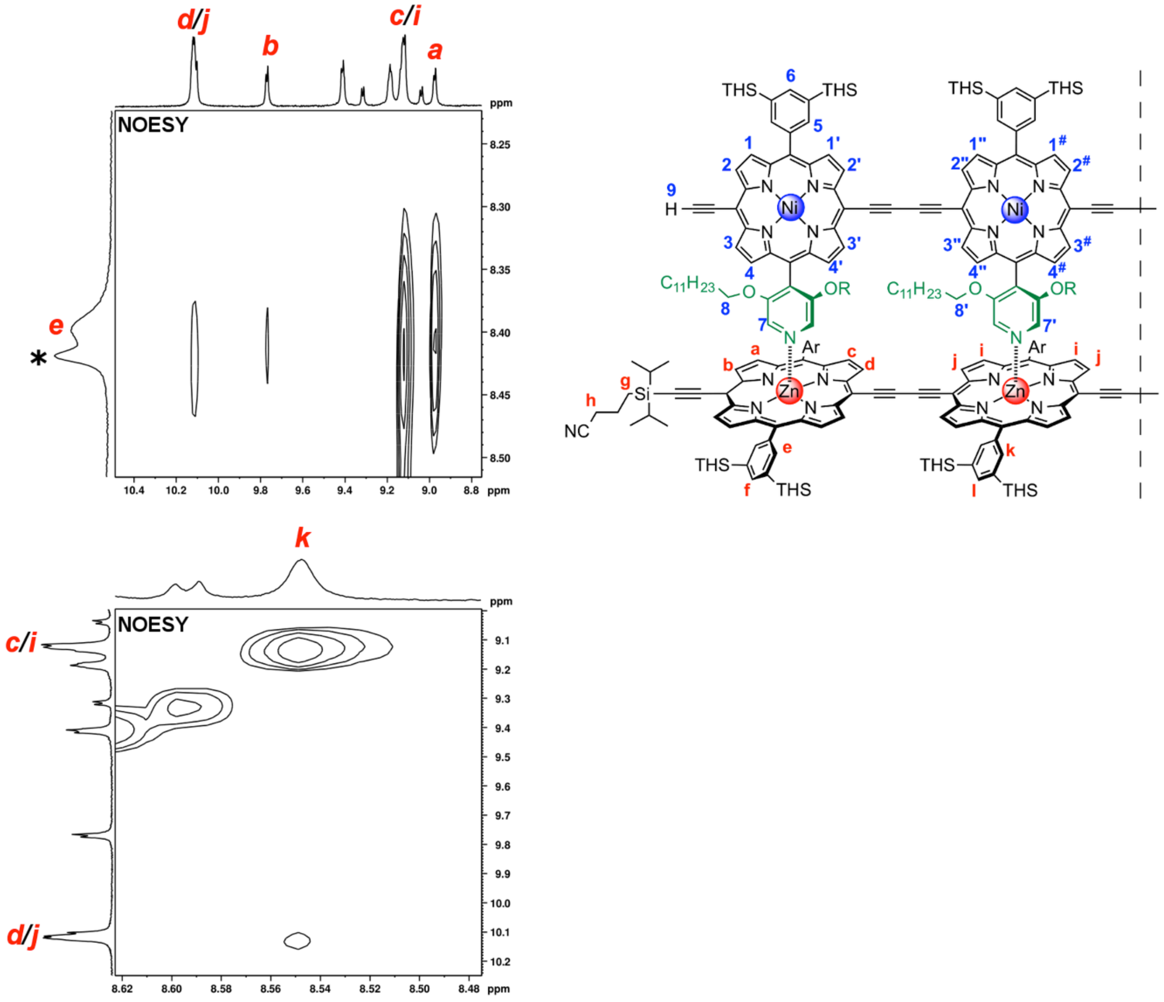

Figure S23 Region of the NOESY spectra of **CPDIPS-ZnP4•NiP4** corresponding to **CPDIPS-ZnP4** β-protons ***a***, ***b***, ***c***, ***d***, ***i*** and ***j*** and aryl side-group protons ***e*** and ***k*** (500 MHz, $CDCl_3$, 298 K). * indicates impurity.

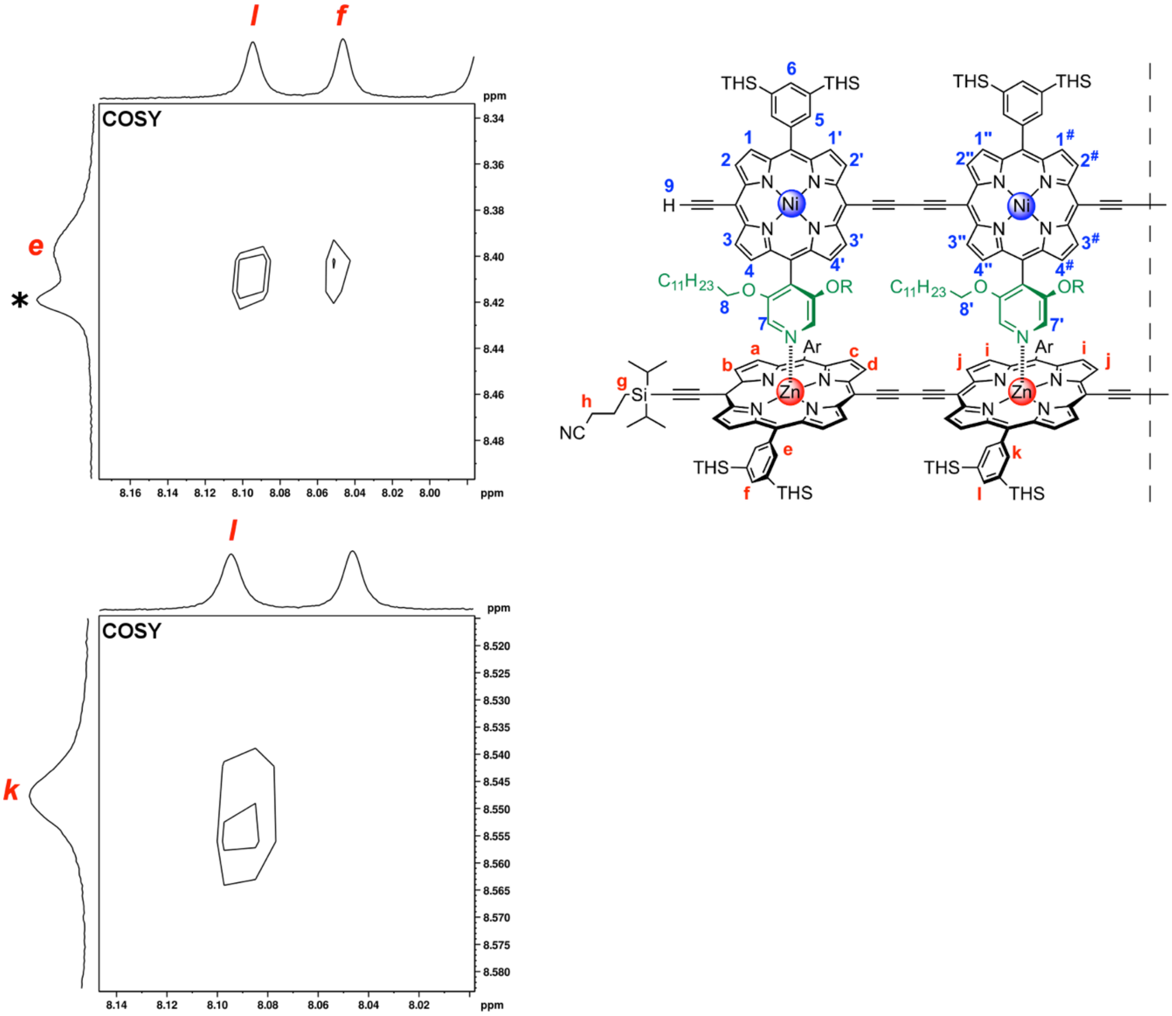

Figure S24 Region of the COSY spectra of **CPDIPS-ZnP4•NiP4** corresponding to **CPDIPS-ZnP4** aryl side-group protons ***e***, ***f***, ***l*** and ***k*** (500 MHz, $CDCl_3$, 298 K). * indicates impurity.

Protecting group protons ***g*** and ***h*** on the Zn-strand were assigned based on their correlation in the COSY spectrum and the relationship of NOEs between proton ***g*** and proton ***b*** (**Figure S25**).

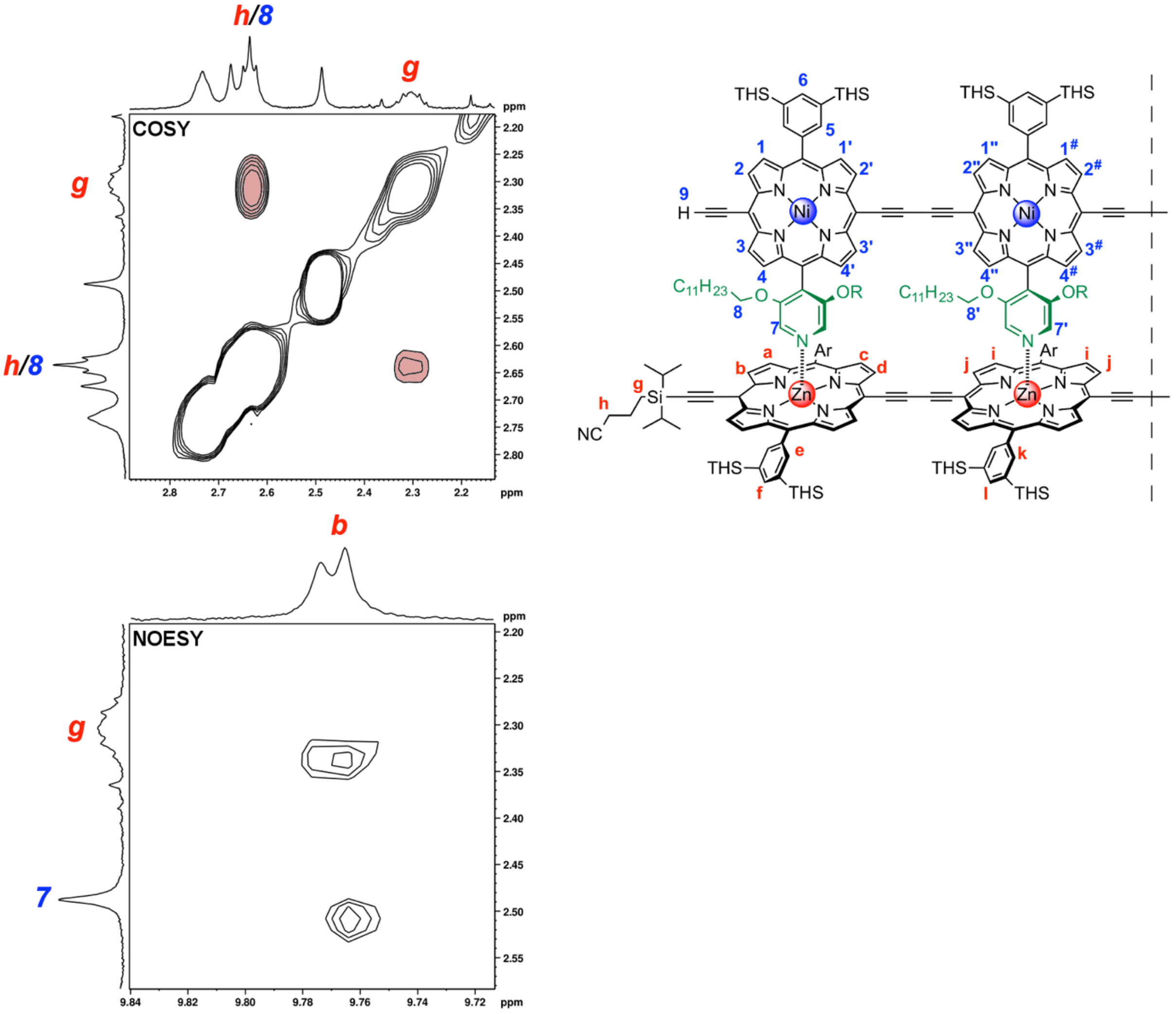

Figure S25 Region of the COSY and NOESY spectra of **CPDIPS-ZnP4•NiP4** corresponding to **CPDIPS-ZnP4** protecting group protons ***g*** and ***h*** and β-proton ***b*** (500 MHz, $CDCl_3$, 298 K). The pink color of the peaks highlights the correlation between protons ***g*** and ***h*** in the COSY spectrum.

Due to the ring current effect, α-protons *7* (outer) and *7′* (inner) on pyridyl side-groups of **NiP4** are strongly shielded and appear as two singlet signals at very low chemical shifts (2.48 and 2.67 ppm, respectively). Two triplet signals of alkyl side-chain proton ***8*** (outer) and ***8′*** (inner) on the pyridyl side-groups of **NiP4** are also shielded from the ring current effect and shifted to the up-field at 2.63 and 2.73 ppm, respectively and the outer proton ***8*** is overlapping with the proton ***h*** of **CPDIPS-ZnP4**.

Furthermore, protons ***8*** and ***8′*** show a correlation with alkyl side chain protons at low chemical shifts (0.5–0.65 ppm) while protons *7* and *7′* do not have this correlation, confirming the assignment of *7*, *7′* and ***8***, ***8′*** in the COSY spectrum (**Figure S26**).

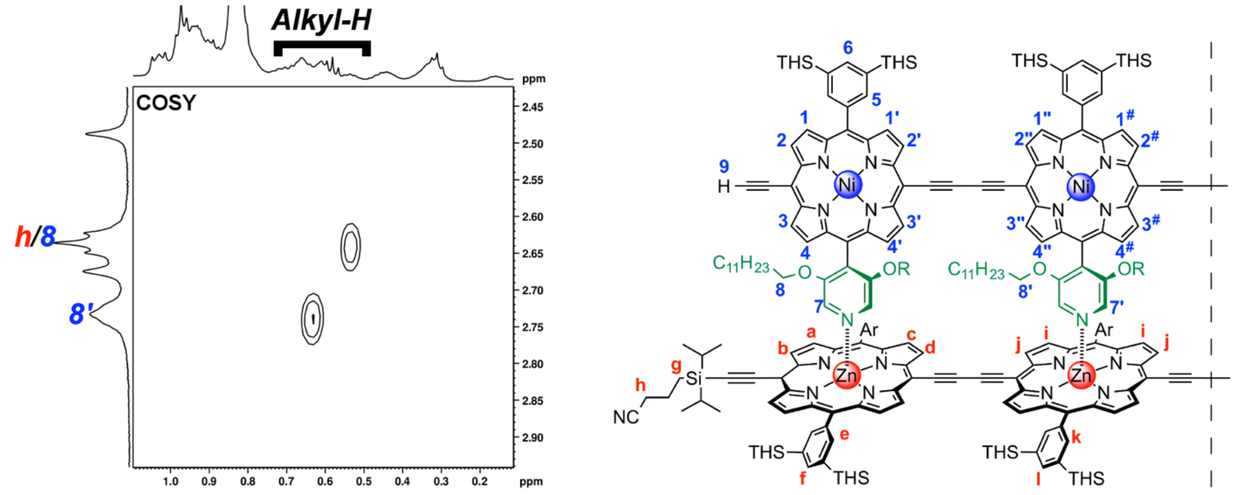

Figure S26 Region of the COSY spectrum of **CPDIPS-ZnP4•NiP4** corresponding to **NiP4** alkyl side-chain protons ***8*** and ***8′*** and alkyl side chain protons (500 MHz, $CDCl_3$, 298 K).

In the NOESY spectrum (**Figure S27**), α-pyridyl protons *7* and *7′* and alkyl side-chain proton ***8*** and ***8′*** on **NiP4** can be distinguished to be either on the outer or the inner Ni porphyrin units based on their NOEs with β-protons ***a***, ***b***, ***c***, ***d***, ***i*** and ***j***, and THS aryl side-group protons ***e*** and ***k*** on **CPDIPS-ZnP4**. The α-proton *7* on the outer Ni porphyrin unit shows a NOE with β-protons ***a***, ***b***, ***c*** and ***d*** and THS aryl side-group proton ***e*** on outer Zn porphyrin unit whereas α-proton ***7′*** on the inner Ni porphyrin unit shows a NOE with β-protons ***i*** and ***j*** and THS aryl side-group proton ***k*** on the inner Zn porphyrin unit.

Additionally, alkyl side-chain proton ***8*** on the outer Ni porphyrin unit shows a NOE with β-proton ***a*** and THS aryl side-group proton ***e*** on the outer Zn porphyrin unit, while alkyl side-chain proton ***8*** on the inner Ni porphyrin unit shows a NOE with β-protons ***i*** and ***j*** and THS aryl side-group proton ***k*** on the inner Zn porphyrin unit (**Figure S27**).

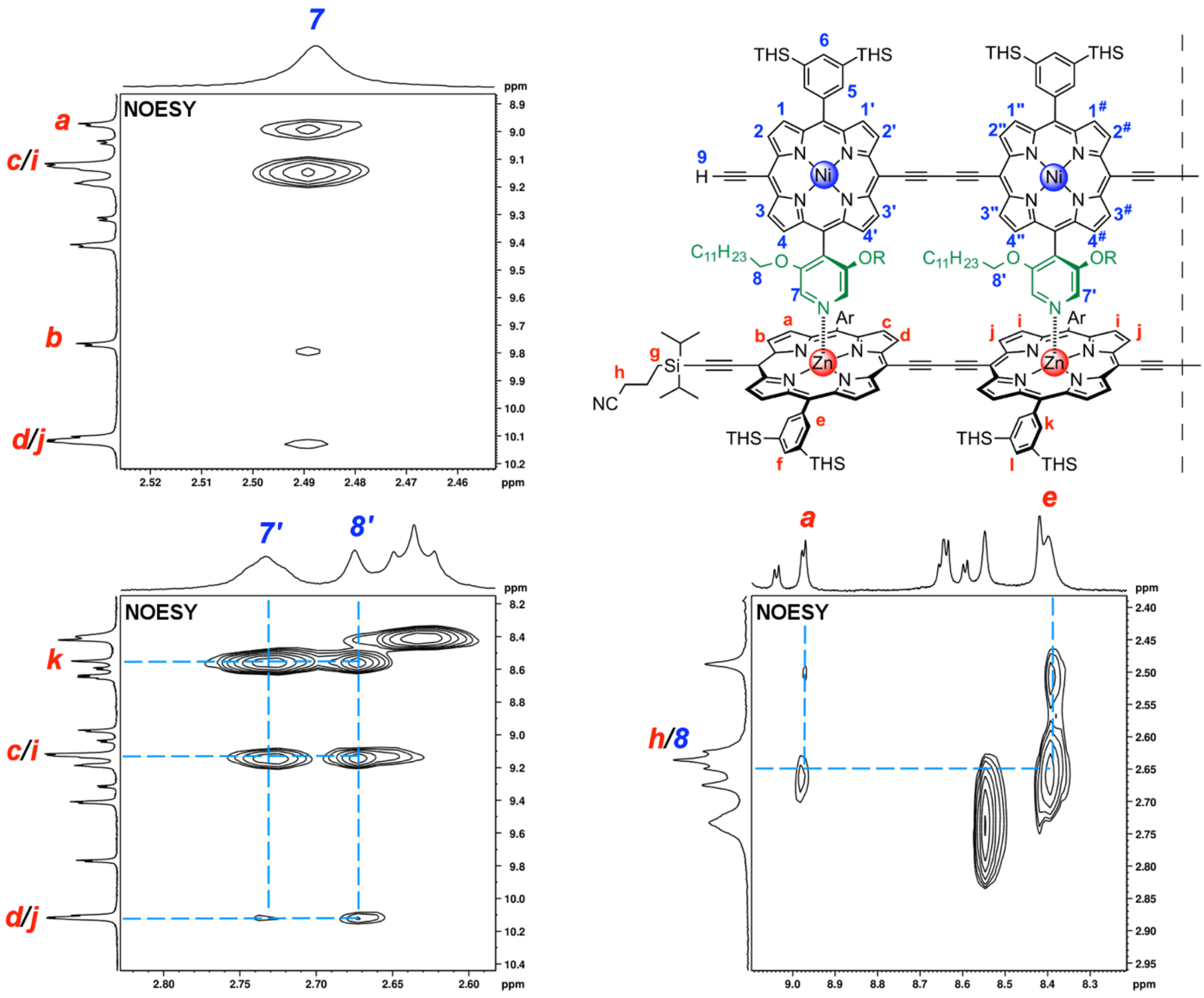

Figure S27 Region of the NOESY spectra of **CPDIPS-ZnP4•NiP4** corresponding to **NiP4** protons *7*, ***7′***, ***8*** and ***8′*** and **CPDIPS-ZnP4** protons ***a***, ***b***, ***c***, ***d***, ***e***, ***i***, ***j*** and ***k*** (500 MHz, $CDCl_3$, 298 K).

Of the β-protons of **NiP4**, protons ***2***, ***2′***, ***2′′***, ***2***$^{\#}$, ***3***, ***3′***, ***3′′*** and ***3***$^{\#}$, which are adjacent to the triple bond, are higher chemical shift than protons ***1***, ***1′***, ***1′′***, ***1***$^{\#}$, ***4***, ***4′***, ***4′′*** and ***4***$^{\#}$. Protons ***2′***, ***2′′***, ***2***$^{\#}$ and ***3′***, ***3′′***, ***3***$^{\#}$, which point towards another porphyrin unit, have a stronger deshielding effect from the butadiyne bridge, providing higher chemical shift (9.41, 9.18 ppm, respectively) than outer protons ***2*** and ***3*** respectively (9.30, 9.03 ppm, respectively).

Apart from that, β-protons ***1***, ***1′***, ***1′′*** and ***1***$^{\#}$ show NOEs with THS aryl side-group proton ***5*** and the coupling between β-protons ***1′***, ***1′′***, ***1***$^{\#}$⇔***2′***, ***2′′***, ***2***$^{\#}$ and ***1***⇔***2*** are also observed in the COSY spectrum. Based on these coupling and NOEs, ***1***, ***1′***, ***1′′***, ***1***$^{\#}$, ***2***, ***2′***, ***2′′***, and ***2***$^{\#}$ are readily assigned on the upper part of the Ni-strand (**Figure S28**).

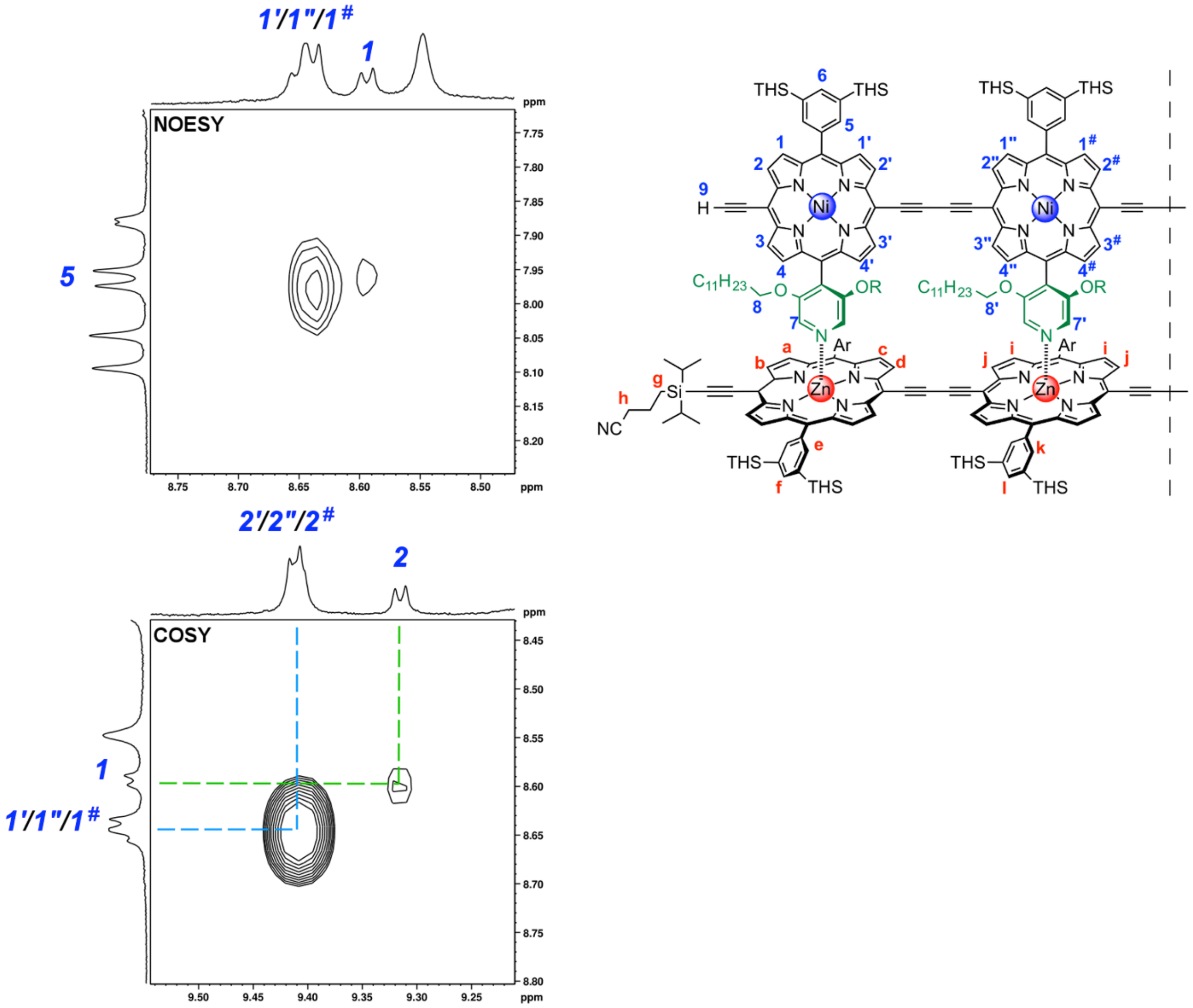

Figure S28 Region of the NOESY and COSY spectra of **CPDIPS-ZnP4•NiP4** corresponding to **NiP4** β-protons ***1***, ***1′***, ***1″***, ***1#***, ***2***, ***2′***, ***2″*** and ***2#*** and THS aryl side-group proton ***5*** (500 MHz, $CDCl_3$, 298 K).

In the lower part of the Ni-strand, β-protons ***4***, ***4′***, ***4″*** and ***4#*** are more shielded due to the short distance to the ring current of the Zn-strand than the other β-protons ***1***, ***1′***, ***1″***, ***1#***, ***2***, ***2′***, ***2″***, ***2#***, ***3***, ***3′***, ***3″*** and ***3#***, resulting in a downfield shift to 7.51–7.64 ppm.

As shown in **Figure S29**, β-protons ***4″*** and ***4#*** on the inner Ni porphyrin unit show NOE with α-pyridyl proton ***7′*** and alkyl side-chain proton ***8′*** from the same unit whereas β-protons ***4*** and ***4′*** on the outer Ni porphyrin unit show NOE with α-pyridyl proton ***7*** and alkyl side-chain proton ***8*** from the same unit. The coupling between protons ***3′***, ***3″***, ***3#***⇔***4′***, ***4″***, ***4#*** and ***3***⇔***4*** is observed in the COSY spectrum. This indicates that protons ***3***, ***3′***, ***3″*** and ***3#*** are also on the lower part of the Ni-strand. The assignment of β-protons ***4***, ***4′***, ***4″*** and ***4#*** can be confirmed, based on their NOEs between protons *7*, *7′*, ***8*** and ***8′*** and their coupling with protons ***3***, ***3′***, ***3″*** and ***3#***.

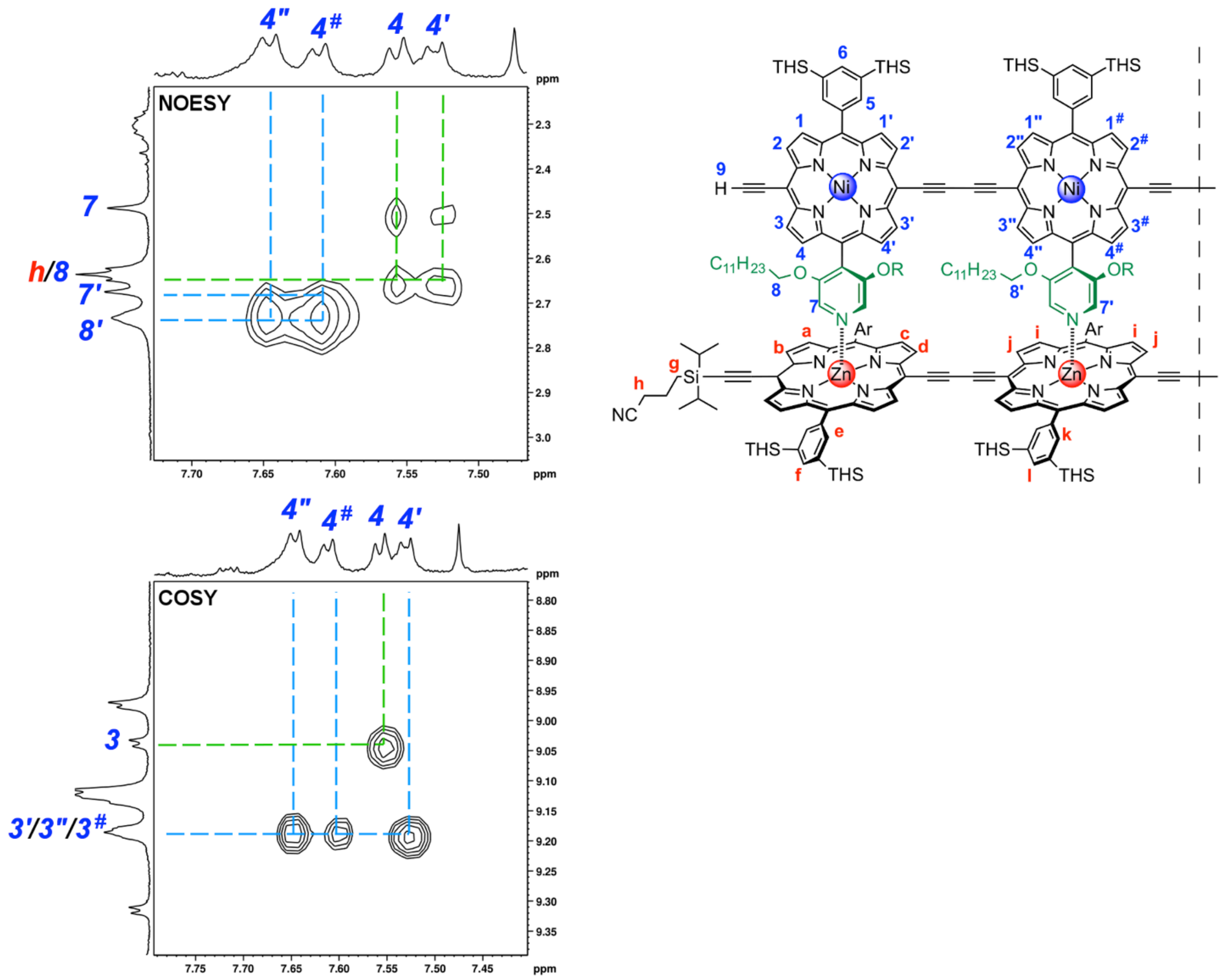

Figure S29 Region of the NOESY and COSY spectra of **CPDIPS-ZnP4•NiP4** corresponding to **NiP4** β-protons ***3***, ***3′***, ***3″***, ***3#***, ***4***, ***4′***, ***4″*** and ***4#*** and protons *7*, *7′*, ***8*** and ***8′*** (500 MHz, $CDCl_3$, 298 K).

The acetylene proton ***9*** is slightly shifted to the up-field since the small effect of ring currents from the Zn-strand.

Apart from the assignment, key signals in the NOESY spectrum reveal the connection between two strands of **NiP4** and **CPDIPS-ZnP4** (**Figure S30**). Observation of NOEs shows the exchange correlation for both the protons of dodecyloxy group and the α-protons on the pyridyl side groups in the Ni-strand with the *ortho*-protons of aryl side groups and the β-pyrrole protons of the porphyrin units in the Zn-strand, confirming the close proximity of both strands.

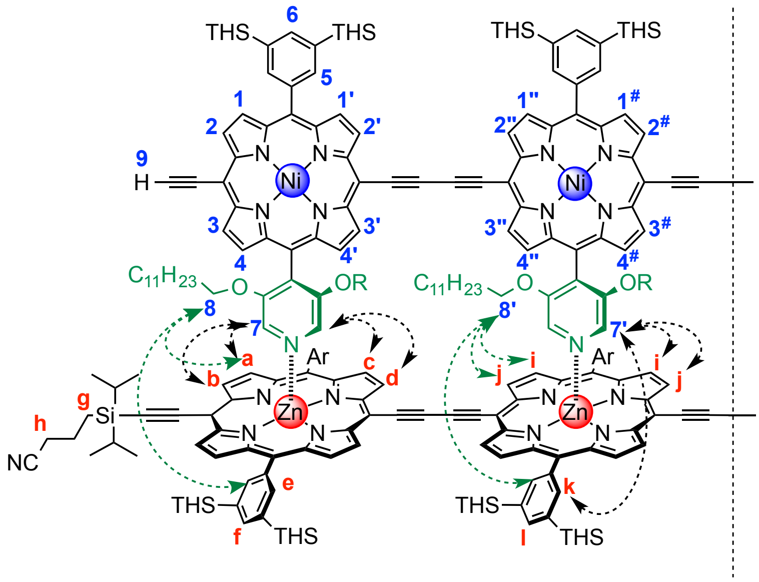

Figure S30 Key NOEs observed between the Ni porphyrin strand and the Zn porphyrin strand. NOEs are observed between both –OCH_2 of dodecyloxy side groups (green dotted line) and the α-protons on the pyridyl side groups (black dotted line) in the Ni-strand with the *ortho*-protons of aryl side groups and the β-pyrrole protons of the porphyrin units in the Zn-strand.

E1.3 Changes in chemical shifts for protons in **CPDIPS-ZnP4•NiP4**

The ^{1}H NMR spectrum of the 4-rung ladder complex was compared to that of the individual corresponding components: **NiP4** and **CPDIPS-ZnP4** (**Figure S31**), revealing significant differences in the aromatic region.

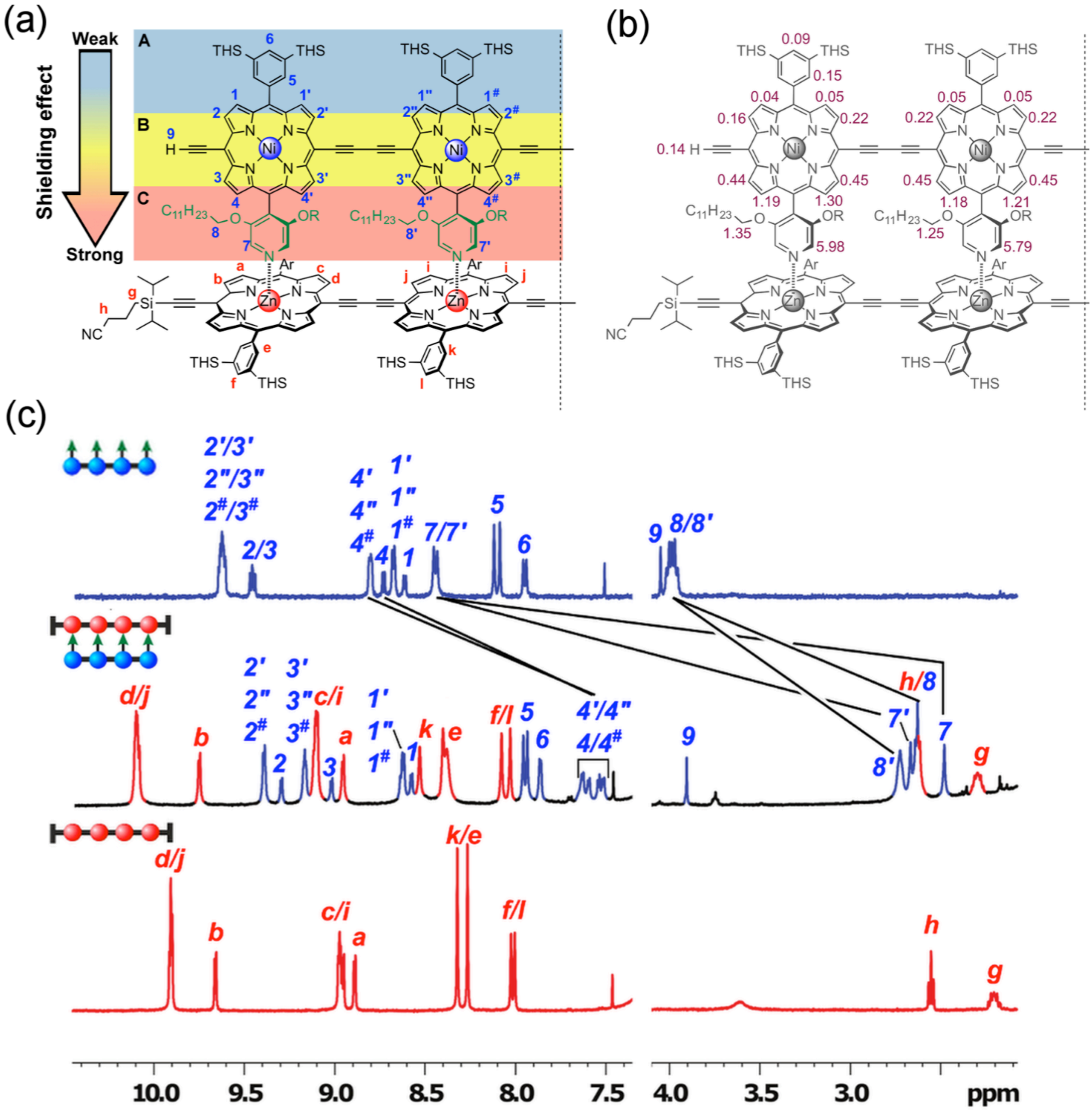

Figure S31 (a) The chemical structure of the 4-rung ladder complex **NiP4•CPDIPS-ZnP4** ($CDCl_3$, 500 MHz, 298 K) and signal assignment for the complex (red letters, protons on **CPDIPS-ZnP4**; blue letters, protons on **NiP4**). Three regions, A, B and C indicate the shielding effect from weak to strong based on the distance from the Zn porphyrin plane. (b) The chemical shift changes ($\Delta\delta$) induced by the shielding effects of the Zn porphyrin plane. (c) Comparison of ^{1}H NMR spectra of the 4-rung ladder complex **NiP4•CPDIPS-ZnP4** and the corresponding individual components (500 MHz, $CDCl_3$, 298 K); (bottom) red signals, ^{1}H NMR of **CPDIPS-ZnP4**; (middle) mixed-color signals, ^{1}H NMR of **NiP4•CPDIPS-ZnP4**; (top) blue signals, ^{1}H NMR of **NiP4**. The black lines indicate the large change in chemical shift ($\Delta\boldsymbol{\delta}$) between the ladder complex and free Ni-strand as a consequence of the strong shielding effect of the ring-current of the Zn-strand.

According to the proposed structure of the complex (**Figure S31a**), the Ni-strand lies within the shielding region of the aromatic ring current of the Zn-strand, causing protons of the Ni-strand at varying distances to experience different degrees of shielding. We roughly divide the degrees of the shielding effect depending on the distances of the protons of the Ni-strands into three regions, A, B and C from weak to strong effect (**Figure S31a**). The summary of the shielding effect from the Zn porphyrin plane is described by the change of chemical shifts ($\Delta\delta$) of **NiP4** protons between free form and bound form (to **CPDIPS-ZnP4**) as shown in **Figure S31b**. The magnitude of the shielding effect increases with decreasing distances from the Zn plane (from region A to C). Furthermore, this effect makes the overlapping signals of all protons in region B distinct from those of the free Ni-strand, splitting up overlapping signals corresponding to protons ***2′***, ***2″***, ***2***$^{\#}$, ***3′***, ***3″***, ***3***$^{\#}$ to two groups of signals corresponding to protons ***2′***, ***2″***, ***2***$^{\#}$ and ***3′***, ***3″***, ***3***$^{\#}$**,** and overlapping signals corresponding to protons ***2*** and ***3*** to two signals corresponding to protons ***2*** and ***3*** (**Figure S31c**). The strongest shielding effect is experienced by all protons in region C as a consequence of the closest distance to the Zn plane, providing the large change in chemical shift ($\Delta\delta$) for the β-pyrrole protons, ***4***, ***4′***, ***4″*** and ***4***$^{\#}$ at 1.17, 1.16, 1.19 and 1.27 ppm, respectively, for the α-protons on the pyridyl side groups, ***7*** and ***7′*** at 5.95 and 5.76 ppm, respectively, and for the protons of dodecyloxy group on the pyridyl side groups, ***8*** and ***8′*** at 1.34 and 1.24 ppm, respectively relative to the corresponding protons on free Ni-strand (**Figure S31c**).

Every single proton of **NiP4** is influenced by the typical shielding effects from Zn porphyrin ring structure. The change of chemical shifts $\Delta\delta$ reflects the distances between protons of **NiP4** and the Zn porphyrin units.

E2. MALDI-ToF Mass analysis of CPDIPS-ZnP4•NiP4

Additional support for the proposed structure of the 4-rung ladder complex was supplied by MALDI-ToF MS technique that proved to be a valuable tool for analyzing non-covalent complexes. As shown in **Figure S32**, the mass spectrum (DTCB matrix) of the complex revealed, besides the two peaks belonging to the individual components **NiP4** (*m*/*z* = 6004.6, expected 6002.3) and **CPDIPS-ZnP4** (*m*/*z* = 7174.9, expected 7175.9), the expected molecular peak of the corresponding supramolecular complex (*m*/*z* = 13,170, expected 13,178). This confirms the identity of the desired complex **CPDIPS-ZnP4•NiP4**.

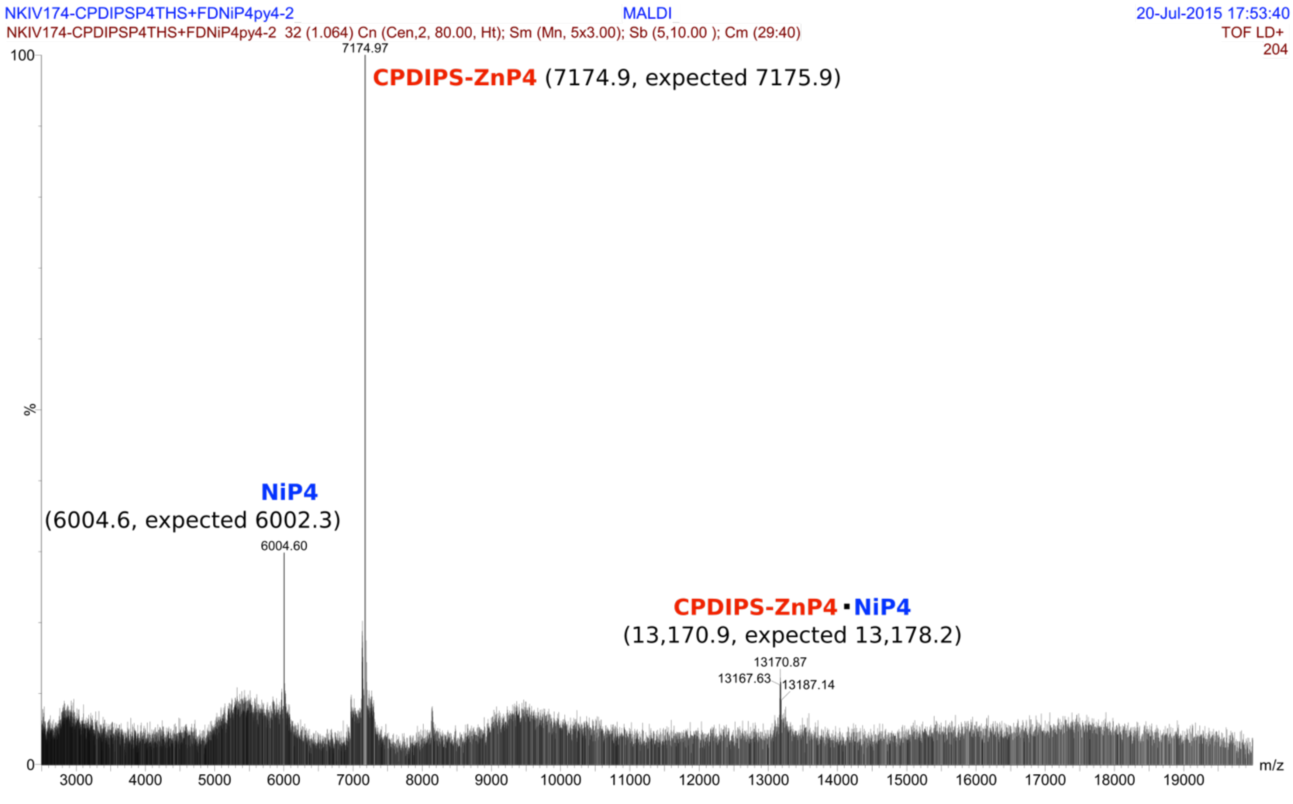

Figure S32 MALDI-ToF mass analysis of the **CPDIPS-ZnP4•NiP4** complex (DTCB as matrix).

F. Comparison of GPC yield ratios of Vernier templating reaction between 479 and 779 nm

The comparison of GPC yields of product-to-substrate ratios of **ZnP12**/**ZnP4** and **ZnP8**/**ZnP4** between 779 nm, where only Zn porphyrins absorb, and 479 nm were investigated, resulting in a good agreement of the values in both wavelengths as shown in **Figure S33**.

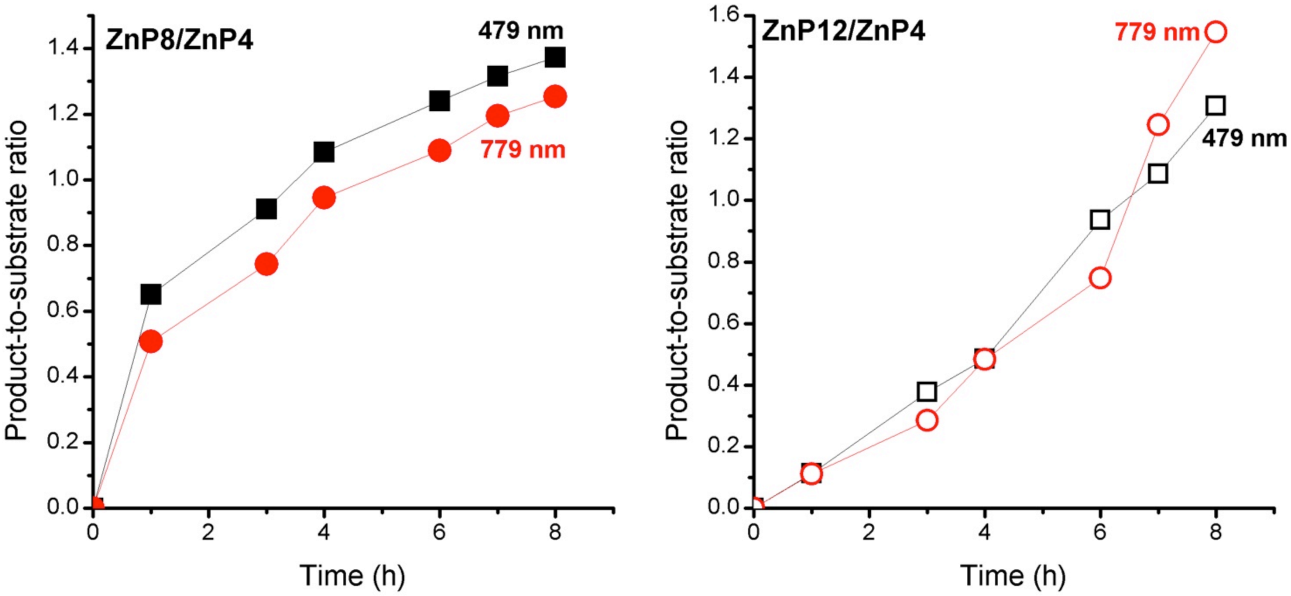

Figure S33 Comparison of the product-to-substrate ratios from the Vernier templating reaction, calculated at different wavelengths. (Left) The product-to-substrate ratios of **ZnP8**/**ZnP4** calculated from the GPC traces at 479 (black solid squares) and 779 nm (red solid circles). (Right) The product-to-substrate ratios of **ZnP12**/**ZnP4** calculated from the GPC traces at 479 (black open squares) and 779 nm (red open circles). The solid lines are a guide to the eye.

G. MALDI-ToF mass analysis of mutual Vernier templating reaction

To support the analyses by the calibrated GPC retention time, the product species in the mutual Vernier templating and the control reactions were also analyzed by the MALDI-ToF mass spectrometry (**Figure S34**). Mass peaks are quite broad due to the low resolution of the detector for high-molecular weight molecules, but it is still sufficient to identify the peaks since all predicted products have a mass difference of at least 2000 Da.

The summary of mass analysis is shown in **Table S1**. In the mutual Vernier templating reaction, the mass spectrum reveals the presence of all substrates **ZnP4** and **NiP6**, the intermediate **ZnP8** and desired products **ZnP12** and **NiP12**. No cross-coupling product **ZnNiP10** was detected from the mutual Vernier templating, confirming the strong template effects. Statistical homocoupling species were observed as expected in both Zn and Ni controls, whereas the pyridine control provided not only both homocoupling products from Zn and Ni species but also heterocoupling products such as **ZnNiP10** as expected.

The mass data are also in good agreement with the assignment of all species in GPC traces as discussed above.

Table S1 The summary of mass data in the mutual Vernier templating and three control reactions

Compound	Exact mass (g/mol) (theoretical)	*m*/*z* (g/mol) (observed)			
		mutual Vernier	Zn control	Ni control	Pyridine control
**ZnP4**	6811.2	7026.5	7134.6		7176.1
**ZnP8**	13620.4	14174.7	14308.7		14379.4
**ZnP12**	20429.6	21302.4	21364.2		21406.5
**ZnP16**	27238.8		28519.4		
**NiP6**	9002.4	9689.3		9684.1	9820.2
**NiP12**	18002.9	19086.8		19238.3	
**NiP18**	27003.3			28780.9	
**ZnNiP10**	15813.6				16888.1
**ZnNiP14**	22622.8				24124.6

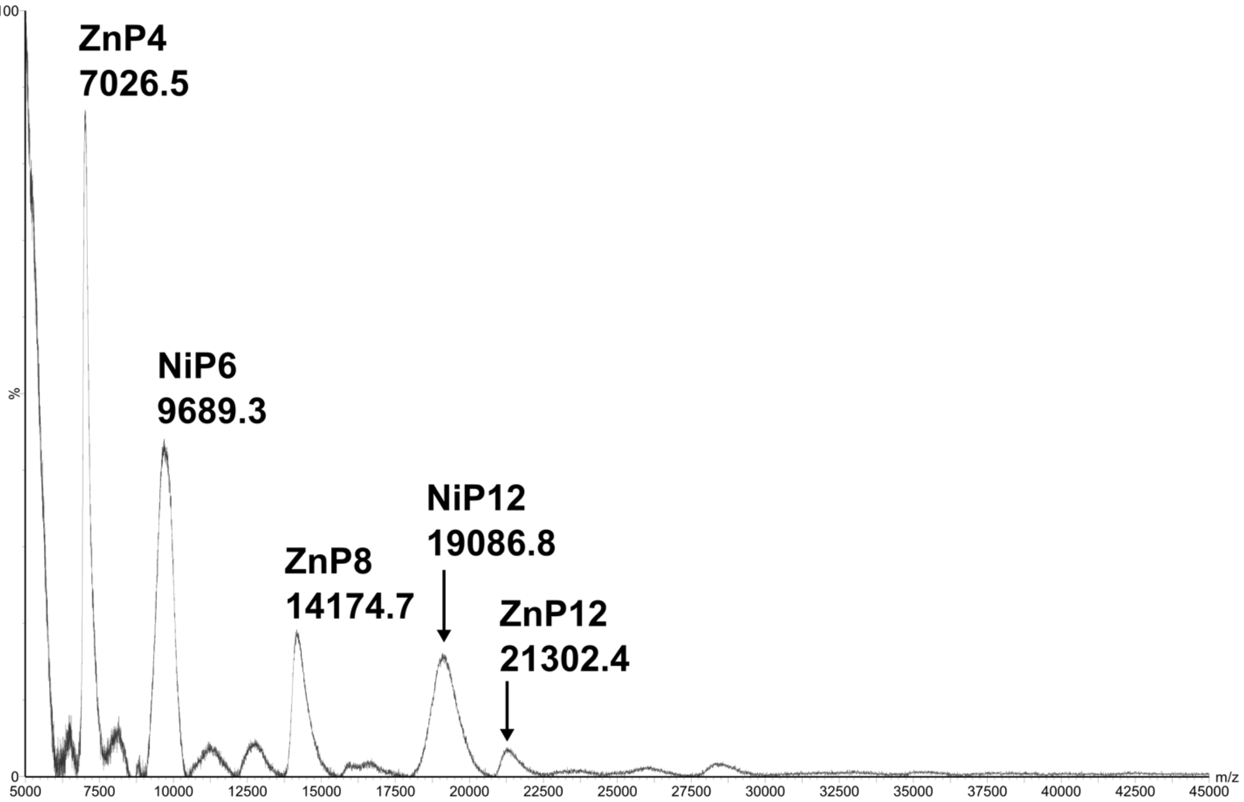

Figure S34 MALDI-ToF mass analysis of the product mixture of the mutual Vernier templating reaction (flight mode: linear, matrix: DTCB).

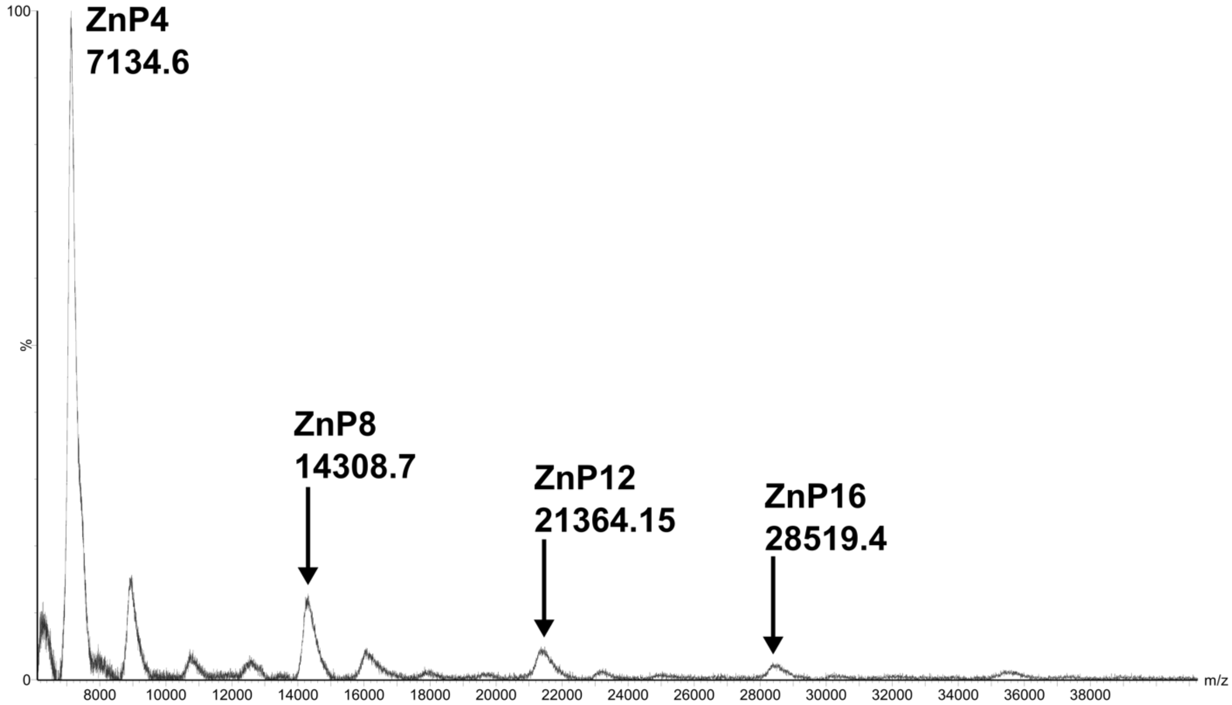

Figure S35 MALDI-ToF mass analysis of the product mixture of the Zn control reaction (flight mode: linear, matrix: DTCB).

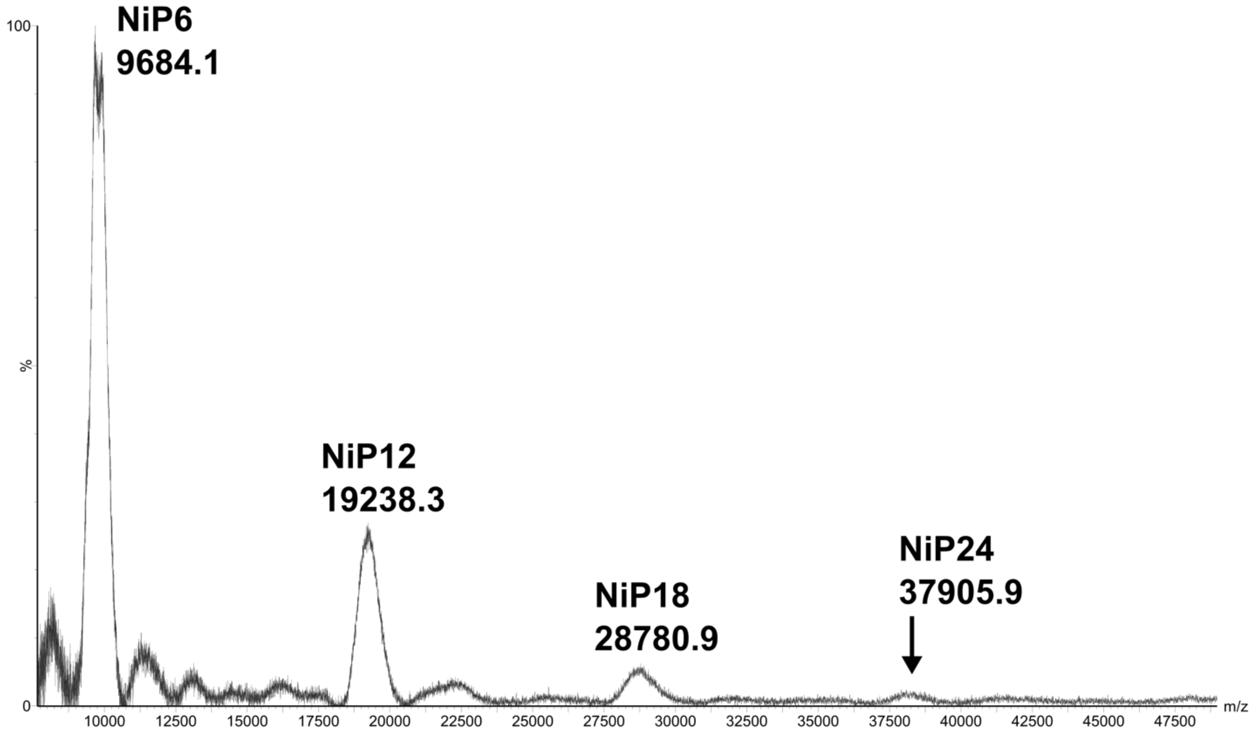

Figure S36 MALDI-ToF mass analysis of the product mixture of the Ni control reaction (flight mode: linear, matrix: DTCB).

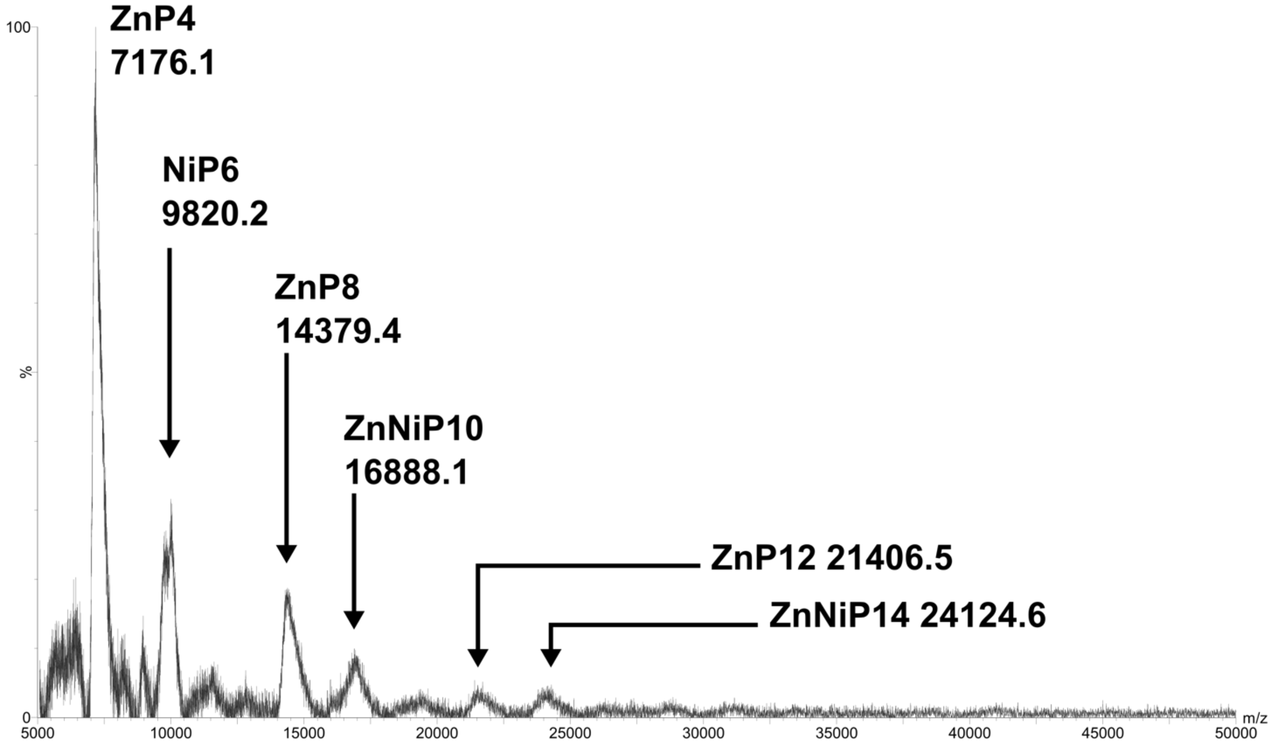

Figure S37 MALDI-ToF mass analysis of the product mixture of the pyridine control reaction (flight mode: linear, matrix: DTCB).

H. Analytical GPC calibration curve

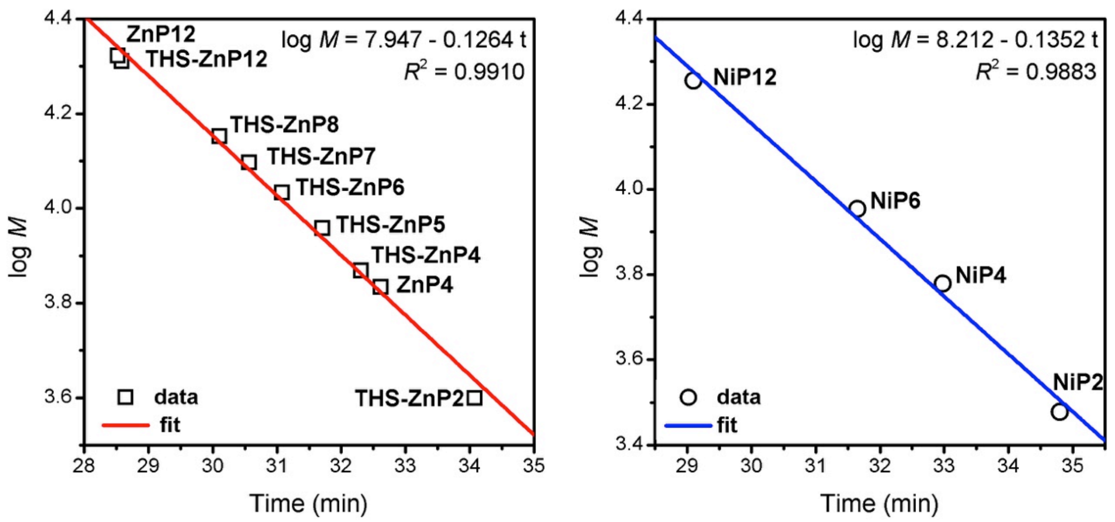

Figure S38 GPC retention times of linear Zn (left) and Ni (right) porphyrin oligomers plotted against log molecular weight. Squares and circles indicate Zn and Ni oligomers, respectively. All data were recorded in THF containing 1% pyridine. The points are fitted to solid lines (red, Zn oligomers; blue, Ni oligomers), according to linear equation (log $M = a - bt$).[6]

Analytical GPC was carried out on a series of linear Zn porphyrin oligomers and a series of linear Ni porphyrin oligomers to confirm the purities of these compounds for analytical GPC calibration (**Figure S38**). Analytical GPC traces of all Zn and Ni species were measured at 591 and 450 nm, respectively eluting with THF/1% pyridine. The intensities of all GPC peaks were normalized.

I. Extinction coefficients of Zn and Ni oligomers per porphyrin unit

Figure S39 shows the overlay of extinction coefficients per porphyrin unit of **NiP4**, **NiP6**, **THS-ZnP4** and **THS-ZnP6**. The green vertical line points out the absorption at 479 nm where all species have the same extinction coefficient per porphyrin unit. Thus, we can calculate analytical yield of all species directly from the absorption at this wavelength in the GPC traces. The blue vertical line indicates the absorption at 779 nm where only Zn porphyrin species absorb dominantly. This is the alternative wavelength to observe yields of Zn porphyrin oligomer products.

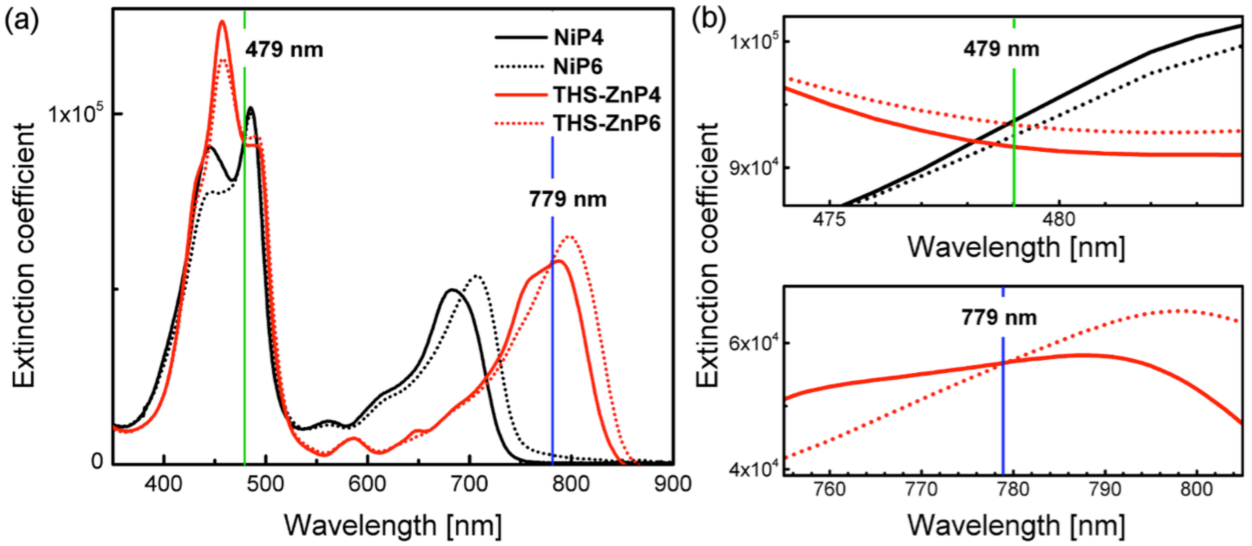

Figure S39 Extinction coefficients per porphyrin unit of **NiP4** (black solid line), **NiP6** (black dot line), **THS-ZnP4** (red solid line) and **THS-ZnP6** (red dot line). (a) Full range spectra. (b) Zoom in at 479 nm (top) and 779 nm (bottom).

J. References

1 R. N. Keller and H. D. Wycoff, *Inorg. Synth.*, 1946, **2**, 1–4.

2 P. Parkinson, C. E. I. Knappke, N. Kamonsutthipaijit, K. Sirithip, J. D. Matichak, H. L. Anderson and L. M. Herz, *J. Am. Chem. Soc.*, 2014, **136**, 8217–8220.

3 C. E. Tait, P. Neuhaus, M. D. Peeks, H. L. Anderson and C. R. Timmel, *J. Am. Chem. Soc.*, 2015, **137**, 8284–8293.

4 H. L. Anderson, *Inorg. Chem.*, 1994, **33**, 972–981.

5 M. D. Peeks, P. Neuhaus and H. L. Anderson, *Phys. Chem. Chem. Phys.*, 2016, **18**, 5264–5274.

6 D. V Kondratuk, L. M. A. Perdigão, A. M. S. Esmail, J. N. O'Shea, P. H. Beton and H. L. Anderson, *Nat. Chem.*, 2015, **7**, 317–322.

7 A. S. Hay, *J. Org. Chem.*, 1962, **27**, 3320–3321.

8 S. Anderson, H. L. Anderson and J. K. M. Sanders, *J. Chem. Soc. Perkin Trans. 1*, 1995, 2255–2267.

9 S. Anderson, H. L. Anderson and J. K. M. Sanders, *J. Chem. Soc. Perkin Trans. 1*, 1995, 2247–2254.

10 G. S. Wilson and H. L. Anderson, *Chem. Commun.*, 1999, 1539–1540.

11 P. N. Taylor and H. L. Anderson, *J. Am. Chem. Soc.*, 1999, **121**, 11538–11545.

12 J. K. Sprafke, B. Odell, T. D. W. Claridge and H. L. Anderson, *Angew. Chem. Int. Ed.*, 2011, **50**, 5572–5575.

13 G. Ercolani and L. Schiaffino, *Angew. Chem. Int. Ed.*, 2011, **50**, 1762–1768.

14 C. A. Hunter and H. L. Anderson, *Angew. Chem. Int. Ed.*, 2009, **48**, 7488–7499.

15 H. J. Hogben, J. K. Sprafke, M. Hoffmann, M. Pawlicki and H. L. Anderson, *J. Am. Chem. Soc.*, 2011, **133**, 20962–20969.
